# Effectiveness and Implementation of Digital Health Interventions on Physiological, Psychological, and Functional Outcomes in Adults With Multimorbidity: Systematic Review and Meta-Analysis of Randomized Controlled Trials

**DOI:** 10.2196/90458

**Published:** 2026-07-28

**Authors:** Ruxia Qiu, Hui Feng, Xiaoyang Li, Hongying Zhou

**Affiliations:** 1 Xiangya School of Nursing, Central South University, No 932, Lushan South Road, Yuelu District, Changsha, 410083, China, 86 15173121969

**Keywords:** multimorbidity, digital health, telemonitoring, self-management, implementation, systematic review, meta-analysis

## Abstract

**Background:**

Multimorbidity involves heterogeneous disease combinations, treatment burden, competing priorities, and complex care pathways. Digital health interventions (DHIs) may support monitoring, self-management, and care coordination, but their effects on health-related outcomes remain uncertain.

**Objective:**

This systematic review and meta-analysis evaluated the effectiveness of DHIs on physiological, psychological, and functional outcomes in adults with multimorbidity, summarized implementation outcomes, and explored intervention-multimorbidity matching patterns.

**Methods:**

PubMed, Web of Science Core Collection, Embase, Cochrane Library, CINAHL with Full Text, Scopus, gray literature sources, trial registries, reference lists, and forward citations were searched through April 7, 2026. English-language randomized or cluster-randomized trials enrolled adults with 2 or more chronic conditions and compared a DHI with a comparator lacking the same digital component. Two reviewers independently screened studies, extracted data, and assessed risk of bias using the Cochrane Risk of Bias 2 tool. Random-effects meta-analyses used restricted maximum likelihood estimation, Hartung-Knapp adjustment, and Nagashima-Noma-Furukawa prediction intervals. Certainty of evidence was assessed using the GRADE (Grading of Recommendations, Assessment, Development and Evaluation) approach.

**Results:**

In total, 36 reports (37 trials) were included. A total of 7 were rated as high risk of bias and 29 as having some concerns; none was rated as low risk overall. No statistically significant pooled effects were observed for glycated hemoglobin, blood pressure, mortality, hospitalization, or readmission, depression response, health-related quality of life, or pain-related functional impact or disability. For glycated hemoglobin, 6 studies (3992 participants) yielded a mean difference of −0.12 percentage points (95% CI −0.36 to 0.11; 95% prediction interval −0.70 to 0.43). For systolic blood pressure, 6 studies including 5596 participants yielded a mean difference of −3.40 mm Hg (95% CI −8.94 to 2.14; 95% prediction interval −19.15 to 13.03). Depression severity was the only outcome whose pooled 95% CI favored the intervention (8 studies; 1861 participants; standardized mean difference −0.49, 95% CI −0.82 to −0.16), but its prediction interval spanned benefit to harm (−1.53 to 0.51). Certainty was low or very low for all 7 GRADE-assessed outcomes. Implementation findings suggested feasibility, especially with monitoring, coaching, or clinician contact, but reporting was heterogeneous.

**Conclusions:**

Current evidence does not support consistent, transferable benefits of DHIs across most outcomes in adults with multimorbidity. Their real-world value may depend less on technology type than on alignment among intervention mechanisms, patient complexity, outcomes, and delivery context. Future DHIs should be adaptive, burden-sensitive, and workflow-integrated, linking digital data to patient priorities, clinician responses, and care coordination.

## Introduction

### Rationale

Multimorbidity, commonly defined as the coexistence of 2 or more chronic conditions, has become a defining feature of aging populations worldwide [1]. It is more than a simple accumulation of separate diseases; it reflects the convergence of biological aging, functional decline, and lifelong exposure to behavioral, environmental, and social risks [1-4]. Multimorbidity affects a substantial proportion of adults globally and becomes increasingly common with age [5]. It is associated with frailty, disability, treatment burden, dependency, and increasing pressure on families, communities, and health systems [1,3,6,7]. Improving the management of multimorbidity has therefore become a major priority for healthy aging and health system redesign [1,6].

Digital health interventions (DHIs) have attracted growing interest as tools to support chronic disease monitoring, self-management, patient-provider communication, and care coordination. By combining remote monitoring, feedback, education, reminders, mobile apps, web-based platforms, and teleconsultation, DHIs may extend care beyond episodic clinical encounters and support more continuous management [8,9]. In multimorbidity, however, digital support is delivered into a more demanding context. Adults with multimorbidity often face polypharmacy, competing self-management demands, functional limitations, cognitive or psychosocial burdens, and treatment goals that may not align neatly across individual conditions [1,7].

Existing reviews provide only a partial basis for judging the role of DHIs in multimorbidity. A prior systematic review and meta-analysis of digital telemedicine interventions identified only 6 studies involving 699 participants and was restricted to interventions with a telemedicine component [10]. More recent reviews have broadened the scope to digital technologies for multimorbidity management, self-management support, and care for older adults with multimorbidity [11-13]. However, most have been scoping or mapping reviews, or have focused on technology types, mechanisms, barriers, facilitators, and use contexts rather than updated pooled effects from randomized evidence. Consequently, uncertainty remains about the effects of DHIs across clinically relevant outcomes and about their implementation in routine multimorbidity care.

Disease-specific evidence suggests that DHIs can improve selected outcomes, including blood pressure and glycated hemoglobin (HbA_1c_), particularly through telemonitoring, mobile health, and web-based platforms [8,9]. However, because these findings arise largely from populations defined by a single index condition, they cannot be assumed to generalize to multimorbidity, where intervention targets, outcome priorities, and care pathways may differ substantially.

Thus, an updated review focused specifically on adults with multimorbidity is needed. This review adopts a broader DHI lens than telemedicine alone and focuses on randomized and cluster-randomized trials. It also considers implementation-related evidence alongside intervention effects, because digital care is unlikely to improve multimorbidity management unless it can reach, engage, and be sustained among people with complex care needs [14]. By linking intervention modality, outcome domain, and patient complexity, this review seeks to clarify not only whether DHIs work but also under what conditions their effects may be more or less plausible.

### Objectives

This systematic review and meta-analysis aimed to evaluate the effects of DHIs in adults with multimorbidity on clinical, psychological, patient-reported, functional, health care use, and survival outcomes; summarize implementation-related evidence, including reach, engagement, or adherence, and feasibility; and descriptively explore patterns between intervention modality, outcome type, and patient complexity.

## Methods

### Registration and Protocol

The review protocol was prospectively registered in PROSPERO (CRD420251067068). The review was reported in accordance with the Preferred Reporting Items for Systematic Reviews and Meta-Analyses (PRISMA) 2020 expanded checklist [15] and the PRISMA-S (PRISMA Extension for Reporting Literature Searches in Systematic Reviews) [16]. The completed PRISMA 2020 and PRISMA-S checklists are provided in Checklist 1 and Checklist 2, respectively. Protocol amendments and deviations are summarized in Multimedia Appendix 1. The main amendments were replacement of the planned network meta-analysis with pairwise meta-analysis because of sparse and disconnected trial networks, addition of implementation-related outcomes, use of GRADE (Grading of Recommendations Assessment, Development and Evaluation) for the summary of findings outcomes, and a supplementary protocol and registry search conducted on March 15, 2026, for contextual interpretation. Primary bibliographic and gray literature searches were updated on April 6 and 7, 2026. No author-contact round was undertaken for missing or unclear data; the review relied on published reports, supplementary materials, trial registrations, and protocols.

### Eligibility Criteria

We included randomized controlled trials (RCTs) and cluster RCTs published in English that enrolled adults aged 18 years or older with multimorbidity and evaluated any form of DHI against usual care, enhanced usual care, nondigital interventions, or other active comparators without the same digital intervention component. Multimorbidity was defined as the coexistence of 2 or more chronic health conditions [1].

Eligible studies were required to report at least 1 relevant outcome related to multimorbidity care, including biomedical outcomes (eg, HbA_1c_, systolic blood pressure [SBP], or diastolic blood pressure [DBP]), patient-reported outcomes (eg, depressive symptoms or quality of life), behavioral outcomes (eg, medication adherence or self-management), or health care use outcomes (eg, hospital readmission, urgent visits, or mortality). Implementation outcomes, such as reach, engagement or adherence, and feasibility, were extracted when reported.

Studies were excluded if they enrolled only single-disease populations, did not include an active digital intervention component, enrolled participants with multiple conditions but did not explicitly target multimorbidity care, were not primary reports of randomized trials, or duplicated outcome data from another included report. When multiple reports referred to the same study, these were collated and treated as a single study. Full eligibility criteria are provided in Multimedia Appendix 2.

### Information Sources

We searched PubMed (National Library of Medicine), Web of Science Core Collection (Clarivate Analytics), Embase (Elsevier), Cochrane Library (Wiley), CINAHL with Full Text (EBSCOhost), and Scopus (Elsevier). Databases were searched individually rather than simultaneously on a single multidatabase platform. Gray literature sources included the US Food and Drug Administration, GreyNet International, OpenGrey, NTIS, and APA PsycExtra, searched using the terms “multimorbidity” and “digital health”; titles and summaries of the first 50 results per site were screened when available. Trial registries, including ClinicalTrials.gov, the WHO International Clinical Trials Registry Platform, and the EU Clinical Trials Register, were searched during revision to identify ongoing or unpublished studies for contextual interpretation. Reference lists of included studies and relevant systematic reviews were screened, and forward citation tracking was conducted in Scopus and Web of Science. Records from supplementary registry searches were included only if full trial results met the eligibility criteria. No study authors, experts, manufacturers, or organizations were contacted for additional studies or data. No other information sources or search methods were used beyond those described earlier.

### Search Strategy

Searches combined controlled vocabulary terms (eg, MeSH, Emtree, and CINAHL Subject Headings) and free-text terms related to multimorbidity and DHIs. The search structure combined 2 topical concepts (multimorbidity and DHIs) with randomized or controlled trial design terms where appropriate. Search strategies were developed by the review authors (RQ and XL) and adapted to the syntax and indexing of each database interface. Eligibility was restricted to English-language publications. Full line-by-line search strategies for all databases, trial registries, and websites, including search dates, platforms, records retrieved, and all limits applied, are provided in Multimedia Appendix 3. No published search filters were used, either as originally designed or in modified form. The search strategies were not substantively adapted from prior reviews and were not formally peer reviewed using the PRESS (Peer Review of Electronic Search Strategies) guideline.

### Selection Process

All records were imported into EndNote (version 21; Clarivate). Duplicates were removed using EndNote’s automated duplicate detection, followed by manual review to identify residual duplicates. Two reviewers (RQ and XL) independently screened titles and abstracts and then assessed full-text reports for eligibility. Disagreements at either stage were resolved through discussion, with arbitration by a third reviewer (HF) when necessary. No automation tools were used in the selection process.

### Data Collection Process

Data were extracted using a standardized form adapted from the Cochrane Consumers and Communication Group’s Quantitative Data Extraction Template [17]. The form was piloted and refined before full extraction. Two reviewers (RQ and XL) independently extracted data, with discrepancies resolved through discussion or consultation with a third reviewer (HF). Outcome data were cross-checked against trial registrations, published protocols, or supplementary materials where available. No automation tools were used for data extraction.

To characterize DHI heterogeneity, 2 reviewers (RQ and HZ) conducted an inductive thematic analysis of intervention descriptions [18]. Intervention components, delivery modes, and intended therapeutic functions were coded and grouped into higher-order descriptive categories. Interrater agreement for the final primary classification was assessed using percentage agreement and Cohen κ [19].

### Data Items

We extracted all outcomes relevant to the review question. These included metabolic biomarkers (eg, HbA_1c_, fasting glucose, triglycerides, and low-density lipoprotein cholesterol), cardiovascular outcomes (eg, SBP and DBP), renal and hepatic markers (eg, creatinine and estimated glomerular filtration rate), patient-reported outcomes (eg, depressive symptoms, self-management, and quality of life), functional outcomes, and health care use or survival outcomes (eg, hospital readmission, urgent visits, and mortality). Other extracted variables included study identifiers, country and setting, trial design, sample size, participant characteristics, multimorbidity profile, details of the intervention and comparator, reported outcomes and time points, and numerical data required for synthesis. We also extracted information relevant to risk of bias assessment, such as prespecified outcomes, primary time points, and planned analysis methods, when these were available from trial registrations, protocols, or supplementary materials.

We additionally extracted implementation-related data on mode of delivery, reach, engagement or adherence, and feasibility from trials that reported relevant information. Reach was defined as the proportion of the intended population that came into contact with, or was recruited into, the intervention, consistent with process evaluation guidance [20]. Engagement or adherence was captured using study-reported indicators of intervention use, initiation, completion, repeated use, or sustained participation. Feasibility was defined pragmatically using reported indicators such as acceptability, retention, implementation burden, provider capacity, safety, or authors’ explicit judgments that the intervention was feasible or acceptable [21]. Because terminology and measurement varied across studies, we accepted conceptually comparable indicators and relied on study authors’ reporting where specific implementation components were not uniformly defined.

When multiple time points were reported for the same outcome, we selected 1 effect estimate per study for the main meta-analysis according to prespecified decision rules. We first extracted the result at the trial’s prespecified primary analysis time point, as stated in the trial report, protocol, or registration. If no primary analysis time point was explicitly specified, we selected the assessment closest to the end of the active intervention; if the intervention duration was not clearly defined, we selected the longest follow-up identified by the trial authors as the main follow-up assessment. Additional time points were summarized narratively or explored in subgroup analyses where appropriate.

### Study Risk of Bias Assessment

Risk of bias was assessed using the revised Cochrane Risk of Bias tool for randomized trials, version 2 (RoB 2) [22]. Outcome-specific RoB 2 assessments were conducted for the main outcome of each included study. When a study reported multiple primary or coprimary outcomes, the outcome specified by trial authors as the main outcome was used where available; otherwise, the most clinically relevant outcome was selected. For cluster RCTs, assessments followed RoB 2 guidance for cluster-randomized designs. Two reviewers (RQ and XL) independently assessed each study across the standard RoB 2 domains. Domain-level judgments and overall judgments were classified as low risk of bias, some concerns, or high risk of bias. Disagreements were resolved through discussion or, when necessary, adjudication by a third reviewer (HF).

### Effect Measures

For continuous outcomes measured on the same scale, pooled effects were summarized as mean differences (MDs) with 95% CIs. For conceptually comparable continuous outcomes measured using different instruments, pooled effects were summarized as standardized mean differences (SMDs; Hedges *g*) with 95% CIs. For dichotomous outcomes, pooled effects were summarized as risk ratios (RRs) with 95% CIs.

### Synthesis Methods

Outcomes were considered eligible for meta-analysis when at least 2 studies or independent study comparisons reported sufficiently comparable outcome definitions, effect metrics, and summary statistics. Outcomes that could not be pooled because of sparse reporting, incompatible metrics, nonharmonizable scoring directions, or substantial clinical or methodological heterogeneity were synthesized narratively.

All meta-analyses were conducted in R (version 4.5.0; R Foundation for Statistical Computing; *meta* package). When SEs of group means or change scores were reported, these were converted to SDs using the formula SD=SE×√n. Studies without usable variance estimates and for which the required data could not be obtained from published reports, supplementary materials, trial registrations, or protocols were excluded from quantitative synthesis.

Random-effects models were used for all meta-analyses, as true intervention effects were expected to vary across populations, care contexts, and intervention configurations [23]. Between-study variance (τ^2^) was estimated using restricted maximum likelihood, and 95% CIs around pooled effects were calculated using the Hartung-Knapp adjustment [24].

Heterogeneity was characterized using Cochran *Q*, τ^2^, *I*^2^, and prediction intervals. *I*^2^ was interpreted as a descriptive index of the proportion of observed variation attributable to between-study heterogeneity rather than within-study sampling error [25]. Prediction intervals were estimated using the Nagashima-Noma-Furukawa confidence distribution approach and interpreted cautiously when few studies contributed to a synthesis [26].

To avoid double counting [27], each trial contributed at most 1 effect estimate per outcome to a given meta-analysis. Independent randomized comparisons based on nonoverlapping participant samples were treated separately when appropriate. For multiarm trials with shared control groups, eligible intervention groups were combined where clinically appropriate; otherwise, only 1 comparison was included for a given outcome in the same meta-analysis.

Exploratory subgroup analyses were undertaken only when outcome definitions were sufficiently comparable and enough studies were available. Follow-up duration was categorized as short-term (≤6 months) or 12 months. Intervention orientation was classified by the dominant intended mechanism of action as patient-facing or clinician-facing. Patient-facing interventions primarily targeted self-management, education, motivation, or behavior change, whereas clinician-facing interventions targeted health care professionals, decision support, workflows, or care delivery processes.

Sensitivity analyses used leave-one-out procedures under the same random-effects framework. These analyses were interpreted primarily for outcomes with at least 3 contributing studies; for 2-study syntheses, leave-one-out analyses reduced the evidence to single-study estimates and were not considered robust tests of pooled stability.

### Reporting Bias Assessment

Small-study effects were planned to be assessed using funnel plots and Egger regression when at least 10 studies were available for a meta-analysis [28]. Formal tests were not conducted for syntheses with fewer than 10 studies.

### Certainty Assessment

We assessed the certainty of evidence using the GRADE approach for 7 key pooled outcomes selected from the quantitative syntheses [29]. Because all included studies were randomized trials, certainty started at high and was downgraded, where appropriate, for risk of bias, inconsistency, indirectness, imprecision, and publication bias, including suspected small-study effects when assessable. No upgrading domains were applied. Publication bias was not downgraded solely because formal tests for small-study effects were not possible. Final ratings were classified as high, moderate, low, or very low certainty [30]. GRADE assessments were conducted independently by 2 reviewers (XL and HZ), with disagreements resolved through discussion.

## Results

### Study Selection

**Figure 1.**
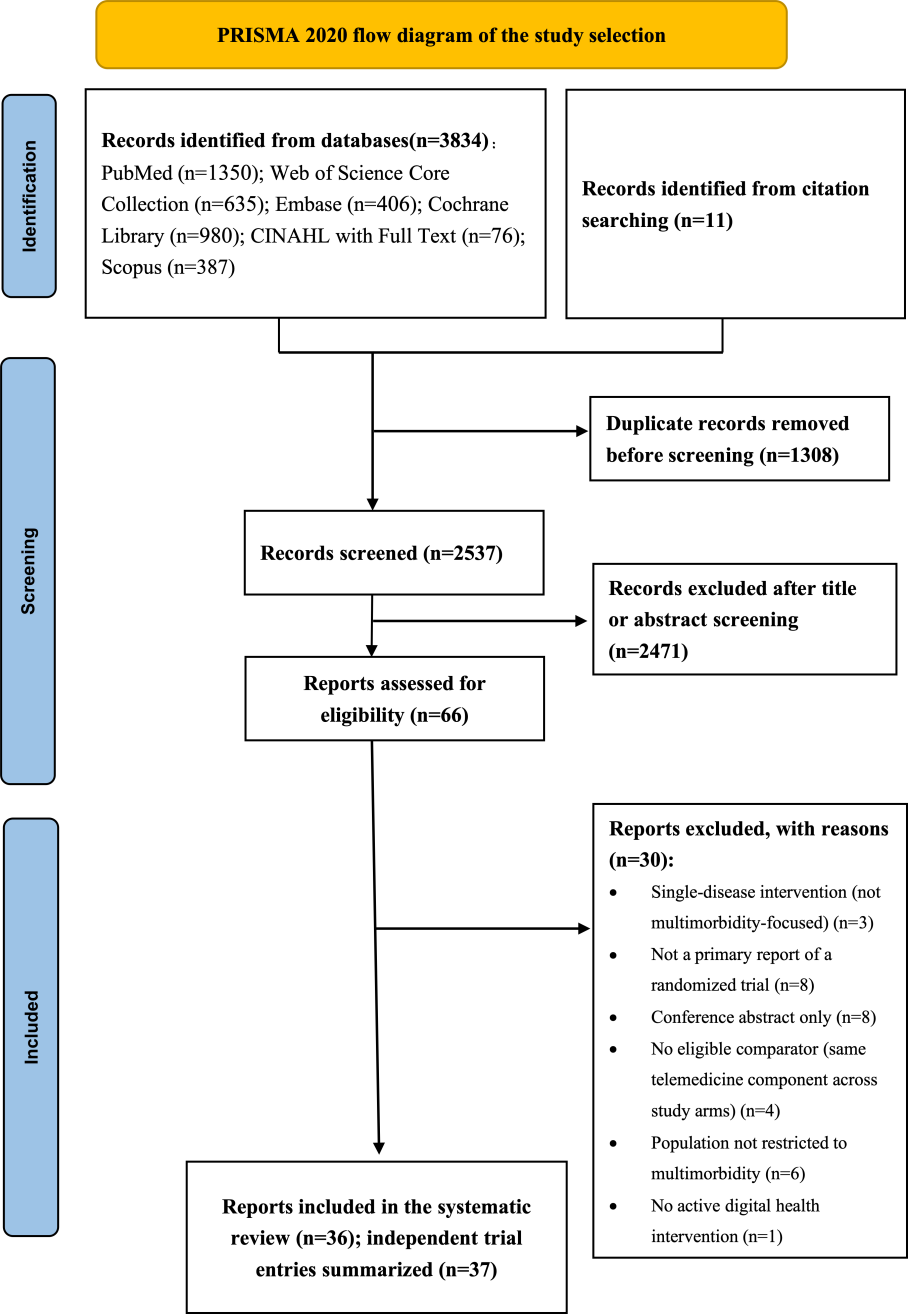
PRISMA (Preferred Reporting Items for Systematic Reviews and Meta-Analyses) 2020 flow diagram of the study selection.

Database searching identified 3834 records, and citation searching identified 11 additional records. After removal of 1308 duplicates, 2537 records underwent title and abstract screening, of which 2471 were excluded. In total, 66 reports were assessed in full text. A total of 30 reports were excluded for the following reasons: the intervention was not multimorbidity focused (n=3), the report was not a primary report of a randomized trial (n=8), the record was a conference abstract only (n=8), no eligible comparator was available because the same telemedicine component was used across study arms (n=4), the population was not restricted to multimorbidity (n=6), or no active DHI was evaluated (n=1). In total, 36 reports, comprising 37 independent trial entries, met the inclusion criteria and were included in the systematic review (Figure 1).

### Study Characteristics

The 36 included reports (37 trials) represented trials conducted across diverse high-, upper-middle-, and lower-middle-income settings in Asia, Europe, North America, South America, and Australia (Multimedia Appendix 4) [31-66]. At the report level, 30 were conducted exclusively in high-income settings, 5 were conducted exclusively in middle-income settings [31-35], and 1 multinational trial spanned settings with different income levels in Asia [36]. In total, 36 studies were published between 2020 and 2025. A total of 6 reports used cluster-randomized designs [31-33,37-39], and Araya et al [35] reported 2 parallel trials, one of which also used cluster randomization in Brazil. A total of 6 studies used a 3-arm design [36,40-44]. Sample size varied widely, from 43 participants [45] to 3698 [32], with a median of 212 (IQR 122‐558) participants.

Participants were generally middle-aged to older adults. Most trials reported a mean or median participant age of 60 years or older, particularly those conducted in primary care, postdischarge, or geriatric settings, whereas several internet-delivered psychological intervention trials focused on younger or midlife samples [46-49]. Where overall sex distribution was reported, female participants comprised 2% to 85.7%, ranging from predominantly male veteran cohorts [41,45] to female-predominant digital mental health and self-management studies [49-52].

Multimorbidity profiles were heterogeneous but were dominated by cardiometabolic and cardiopulmonary combinations. The most commonly targeted dyad was coexisting type 2 diabetes and hypertension [32,34,41,53-55]. Other common profiles included cardiovascular or metabolic disease with renal comorbidity [36,45], chronic obstructive pulmonary disease with heart failure [56], and physical conditions co-occurring with depression or other psychological distress, such as chronic back pain, knee osteoarthritis, insomnia, or generalized distress [35,42,46,48-50]. Several studies also focused on clinically complex or vulnerable populations, including patients with polypharmacy, frailty, high readmission risk, or older adults living alone [38,39,51,57-60].

### Intervention Characteristics

Intervention content, delivery mode, and intensity varied substantially across studies (Multimedia Appendix 4). Broadly, the included DHIs comprised remote physiological monitoring programs, web- or app-based self-management platforms, telephone-delivered coaching or collaborative care models, and digital decision-support or blended psychosocial interventions. Telemonitoring approaches typically combined biometric devices with smartphone, tablet, or web-based interfaces and clinician review or feedback, particularly for cardiometabolic or cardiopulmonary conditions [41,45,53,55-59,61]. Web- or app-based self-management platforms most often integrated support for physical activity, diet, symptom monitoring, medication adherence, or health education with reminders, coaching, or platform-based interaction [31,34,36,43,51,54,62-64]. Telephone-delivered coaching or collaborative care models generally provided structured counseling, psychosocial support, or case management through scheduled calls [40,65,66]. A further subset of trials evaluated digital decision-support tools, integrated care platforms, or blended psychosocial interventions, including electronic decision-support systems, shared decision-making tools, medication review platforms, and internet-delivered cognitive behavioral or behavioral activation programs [32,33,35,37-39,42,46-50,52,60].

Intervention duration ranged from 8 weeks to 24 months. Shorter interventions, often lasting 6 to 12 weeks, were common among web-based self-management and internet-delivered psychological programs [35,42,44,47,48,50-52,61]. Interventions lasting approximately 3 to 6 months were more typical of telemonitoring, telehealth education, or coaching-based models [34,41,45,54,57,59,66]. Longer interventions of 12 months or more were common in integrated care, remote monitoring, and chronic disease management platforms [31,33,36,40,55,58,62,64].

Delivery intensity also varied markedly. Some interventions required daily or near-daily biometric monitoring or symptom reporting, often with automated feedback or clinician review [41,45,53,54,57,59]. Others relied on weekly or biweekly contacts to reinforce exercise, self-management, or adherence [40,51,56,65]. Internet-delivered cognitive behavioral and blended psychosocial programs typically used sequential weekly modules, sessions, or lessons, with varying levels of therapist or e-coach support [42,46-50,52]. By contrast, integrated care and decision-support models more often combined digital tools with periodic clinical review, case conferencing, or medication review rather than high-frequency self-monitoring [32,33,36-39,60]. Overall, the included trials varied substantially in intervention dose, duration, automation, and degree of clinician involvement.

### Risk of Bias in Studies

**Figure 2.**
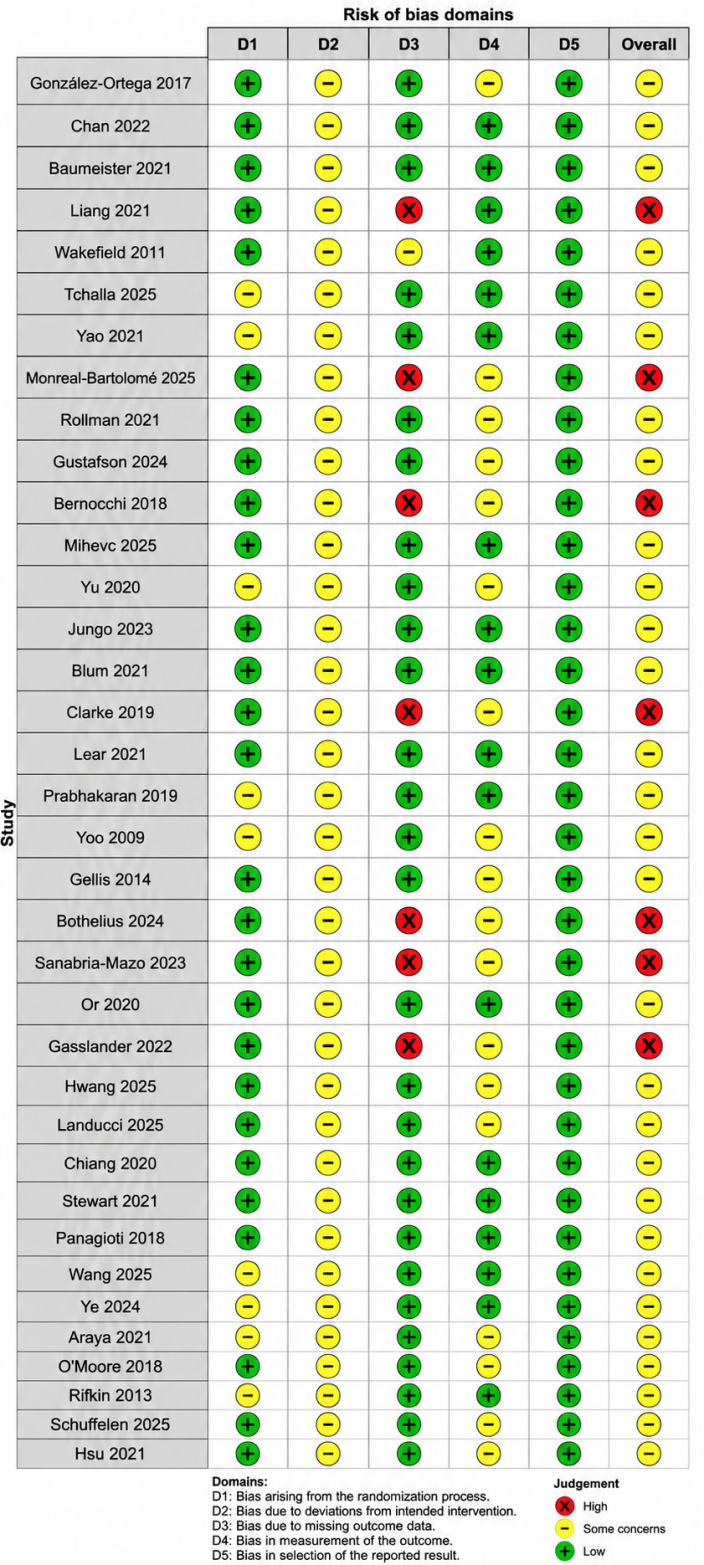
Risk-of-bias assessment of included randomized and cluster-randomized trials using RoB 2 [31-66]. RoB 2: Risk of Bias tool for randomized trials, version 2.

Based on the main outcome used for the RoB 2 judgment, 7 of 36 (19.4%) studies were judged to be at high risk of bias, and 29 (80.6%) were judged as having some concerns; no study was rated as having low risk of bias overall (Figure 2). All high-risk judgments were attributable to bias due to missing outcome data, which was identified in 7 studies. Across domains, some concerns were most frequent for deviations from intended interventions, which applied to all 36 studies, followed by measurement of the outcome in 18 (50%) studies and the randomization process in 9 (25%) studies. Concerns about deviations from intended interventions mainly reflected open-label delivery, limited feasibility of blinding, and potential cointerventions, differential adherence, or behavior change in digital and behavioral intervention trials. Concerns about outcome measurement were more common in open-label studies relying on self-reported outcomes. All studies were judged to be at low risk of bias in selection of the reported result. Detailed domain-level judgments are provided in Multimedia Appendix 5 [31-66].

### Results of Individual Studies

**Figure 3.**
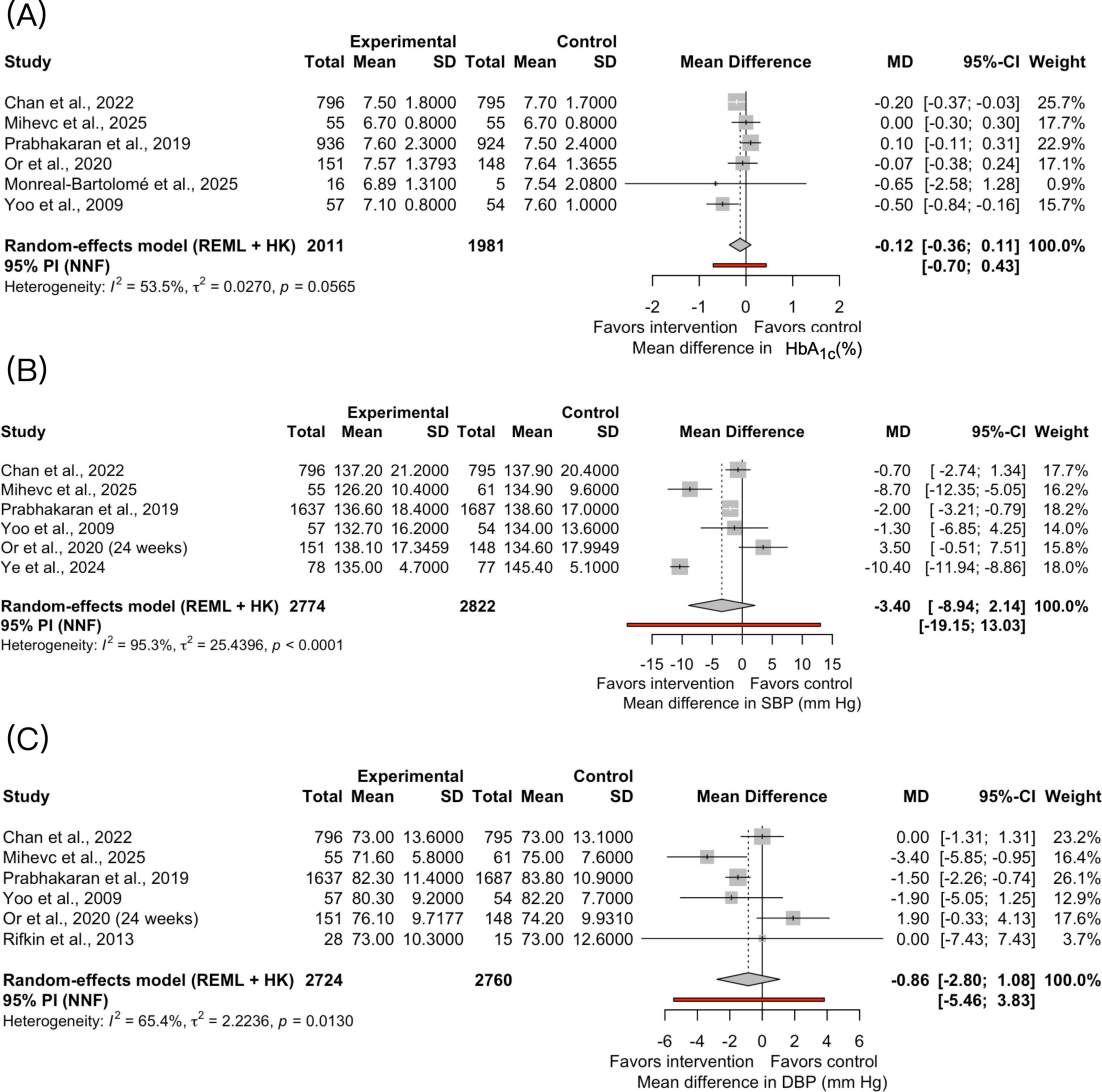
Forest plots of pooled effects of digital health interventions on physiological outcomes in adults with multimorbidity. Panels show pooled effects for (A) HbA_1c_, (B) SBP, and (C) DBP. Negative MDs favor the intervention. Pooled estimates were calculated using random-effects REML models with HK 95% CIs. Red bars indicate 95% PIs [32,34,36,45,52-55]. DBP: diastolic blood pressure; HbA_1c_: glycated hemoglobin; HK: Hartung-Knapp; MD: mean difference; NNF: Nagashima-Noma-Furukawa; PI: prediction interval; REML: restricted maximum likelihood; SBP: systolic blood pressure.

**Figure 4.**
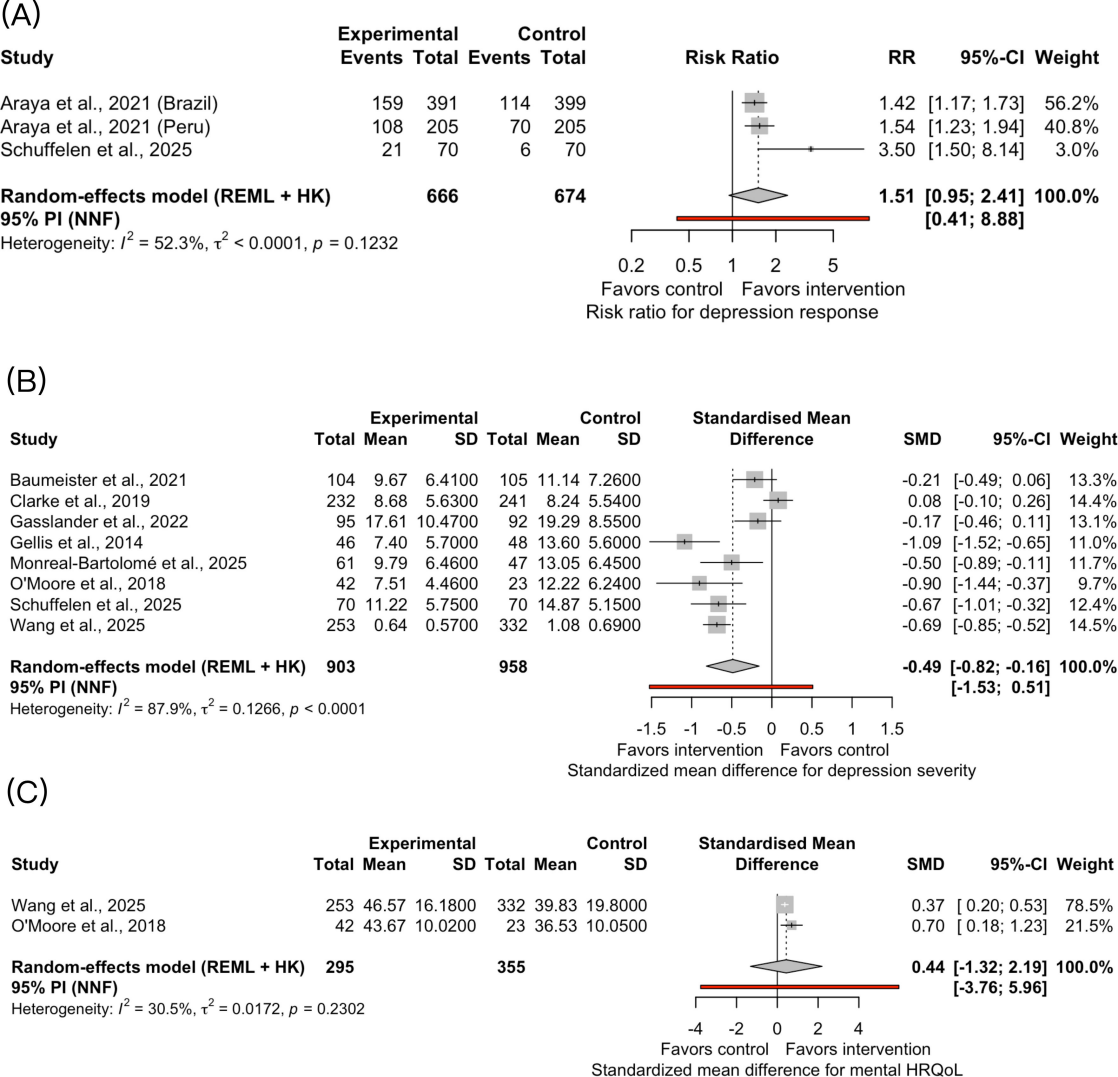
Forest plots of pooled effects of digital health interventions on psychological outcomes in adults with multimorbidity. Panels show pooled effects for (A) depression response, (B) depression severity, and (C) mental HRQoL. For depression response, RRs above 1.0 favor the intervention. For depression severity, negative SMDs favor the intervention. For mental HRQoL, positive SMDs favor the intervention. Pooled estimates were calculated using random-effects REML models with HK 95% CIs. Red bars indicate 95% PIs [33,35,46,48-50,52,59,63]. HK: Hartung-Knapp; HRQoL: health-related quality of life; NNF: Nagashima-Noma-Furukawa; PI: prediction interval; REML: restricted maximum likelihood; RR: risk ratio; SMD: standardized mean difference.

**Figure 5.**
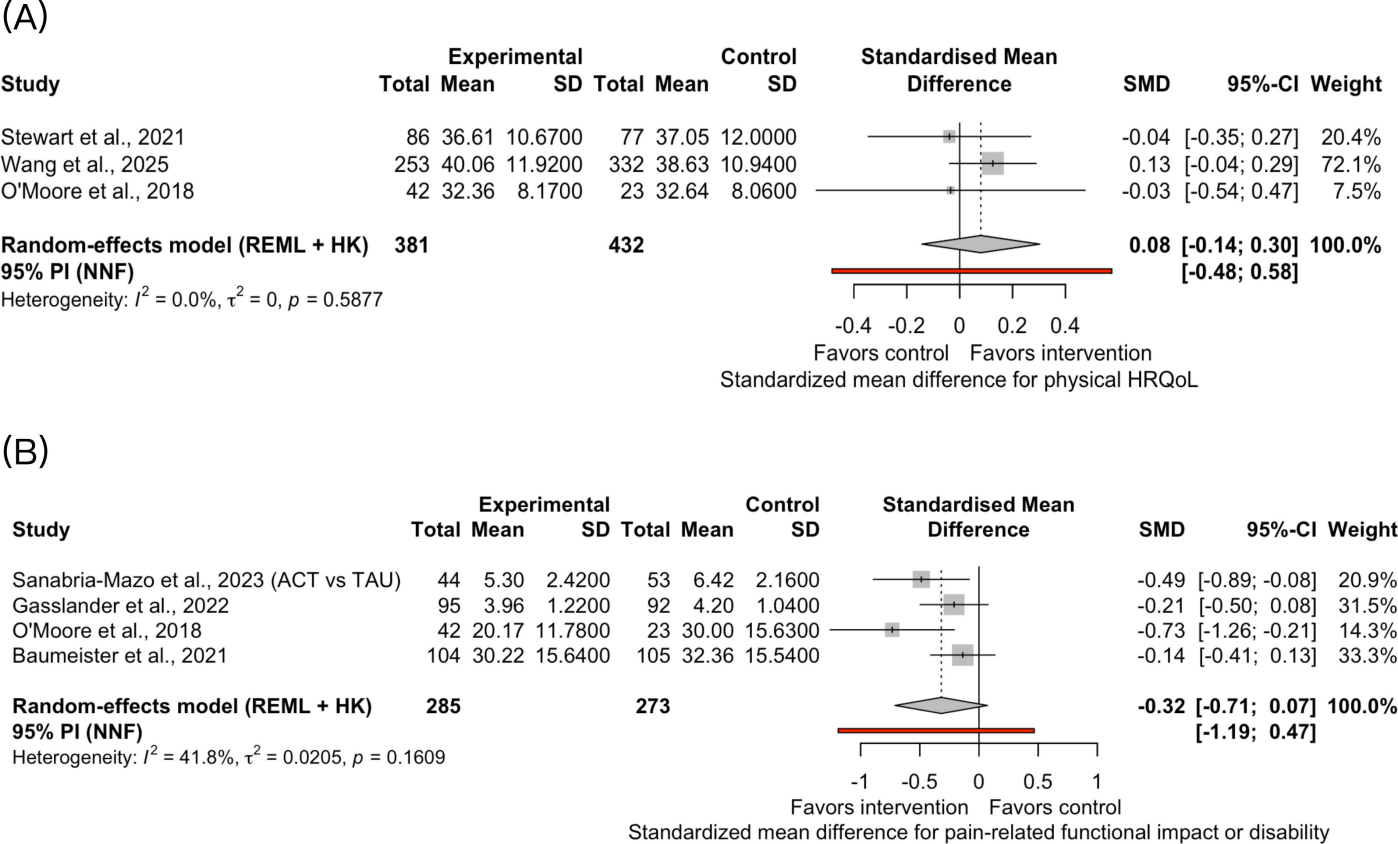
Forest plots of pooled effects of digital health interventions on functional outcomes in adults with multimorbidity. Panels show pooled effects for (A) physical HRQoL and (B) pain-related functional impact or disability. Positive SMDs favor the intervention for physical HRQoL, whereas negative SMDs favor the intervention for pain-related functional impact or disability. Pooled estimates were calculated using random-effects REML models with HK 95% CIs. Red bars indicate 95% PIs [33,42,46,48,50,60]. HK: Hartung-Knapp; HRQoL: health-related quality of life; NNF: Nagashima-Noma-Furukawa; PI: prediction interval; REML: restricted maximum likelihood; SMD: standardized mean difference.

**Figure 6.**
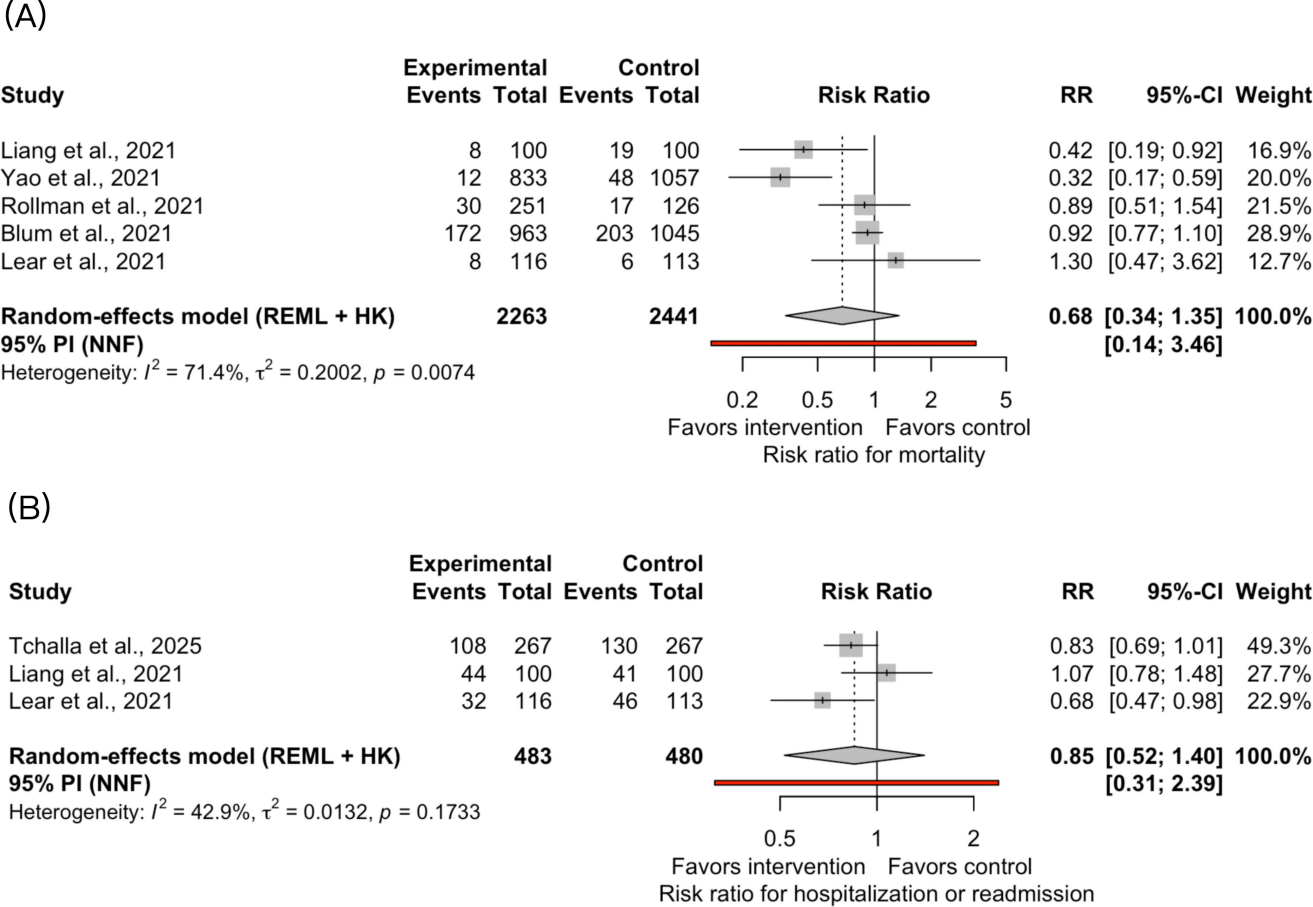
Forest plots of pooled effects of digital health interventions on survival and health service use outcomes in adults with multimorbidity. Panels show pooled effects for (A) mortality and (B) hospitalization or readmission. RRs below 1.0 favor the intervention. Pooled estimates were calculated using random-effects REML models with HK 95% CIs. Red bars indicate 95% PIs [31,39,40,57,58,64]. HK: Hartung-Knapp; NNF: Nagashima-Noma-Furukawa; PI: prediction interval; REML: restricted maximum likelihood; RR: risk ratio.

Study and intervention characteristics are summarized in Multimedia Appendix 4. Individual results for pooled outcomes are presented in the forest plots (Figures 3-6). Outcomes, alternative time points, trial-reported analyses, and study-level findings not included in the main pooled estimates are summarized in Multimedia Appendix 6 [31-66].

### Results of Syntheses

#### Characteristics of Contributing Studies

The number of studies or independent study comparisons contributing to each meta-analysis ranged from 2 to 8. Syntheses of HbA_1c_, SBP, and DBP were derived primarily from trials in cardiometabolic multimorbidity populations and mainly evaluated telemonitoring or digital self-management interventions. Depression severity and depression response syntheses were drawn largely from studies enrolling populations with depression-related multimorbidity or mental-physical comorbidity and evaluated web-based, app-based, or digitally supported psychological interventions. Mortality and hospitalization or readmission additionally included studies enrolling more clinically complex populations, including individuals with heart failure, atrial fibrillation, polypharmacy, multiple chronic diseases, or elevated postdischarge risk. Mental and physical health-related quality of life (HRQoL) syntheses were based on a small number of studies using different quality-of-life instruments, and the synthesis of pain-related functional impact or disability combined closely related measures of pain interference, pain-related disability, and physical function within musculoskeletal or chronic pain populations. Risk of bias among contributing studies was variable, with the most common concerns relating to deviations from intended interventions and outcome measurement, and less frequent concerns relating to the randomization process.

#### Results of Statistical Syntheses

Meta-analysis was undertaken for physiological outcomes, including HbA_1c_, SBP, and DBP (Figure 3); psychological outcomes, including depression response, depression severity, and mental HRQoL (Figure 4); functional outcomes, including physical HRQoL and pain-related functional impact or disability (Figure 5); and survival and health service use outcomes, including mortality and hospitalization or readmission (Figure 6). For mean-difference outcomes, negative values favored the intervention. For SMD outcomes, negative values favored the intervention for depression severity and pain-related functional impact or disability, whereas positive values favored the intervention for mental and physical HRQoL. For RRs, values below 1.0 favored the intervention for mortality and hospitalization or readmission, whereas values above 1.0 favored the intervention for depression response.

For physiological outcomes, the pooled estimates for HbA_1c_, SBP, and DBP were compatible with little or no effect (Figure 3). For HbA_1c_, 6 studies including 3992 participants yielded a pooled MD of −0.12 percentage points (95% CI −0.36 to 0.11; *I*^2^=53.5%; τ^2^=0.0270), with a 95% prediction interval from −0.70 to 0.43. For SBP, 6 studies including 5596 participants yielded a pooled MD of −3.40 mm Hg (95% CI −8.94 to 2.14; *I*^2^=95.3%; τ^2^=25.4396), with a 95% prediction interval from −19.15 to 13.03 mm Hg. For DBP, 6 studies including 5484 participants yielded a pooled MD of −0.86 mm Hg (95% CI −2.80 to 1.08; *I*^2^=65.4%; τ^2^=2.2236), with a 95% prediction interval from −5.46 to 3.83 mm Hg.

For psychological outcomes, the point estimate for depression response favored the intervention, but the 95% CI crossed the null (Figure 4). Across 3 independent comparisons including 1340 participants, the pooled RR was 1.51 (95% CI 0.95-2.41; *I*^2^=52.3%; τ^2^<0.0001), with a 95% prediction interval from 0.41 to 8.88. For depression severity, the pooled estimate favored the intervention. Across 8 studies including 1861 participants, the pooled SMD was −0.49 (95% CI −0.82 to −0.16; *I*^2^=87.9%; τ^2^=0.1266), with a 95% prediction interval from −1.53 to 0.51. This indicates that, although the average pooled effect favored intervention, the effect in a future comparable setting could range from benefit to harm. For mental HRQoL, 2 studies including 650 participants yielded a pooled SMD of 0.44 (95% CI −1.32 to 2.19; *I*^2^=30.5%; τ^2^=0.0172), with a wide 95% prediction interval from −3.76 to 5.96.

For functional outcomes, no statistically significant pooled effects were observed for physical HRQoL or pain-related functional impact or disability (Figure 5). For physical HRQoL, 3 studies including 813 participants yielded a pooled SMD of 0.08 (95% CI −0.14 to 0.30; *I*^2^=0.0%; τ^2^=0), with a 95% prediction interval from −0.48 to 0.58. For pain-related functional impact or disability, 4 studies including 558 participants yielded a pooled SMD of −0.32 (95% CI −0.71 to 0.07; *I*^2^=41.8%; τ^2^=0.0205), with a 95% prediction interval from −1.19 to 0.47.

For survival and health service use outcomes, no statistically significant pooled effects were observed for mortality or hospitalization or readmission (Figure 6). For mortality, 5 studies including 4704 participants and 523 events yielded a pooled RR of 0.68 (95% CI 0.34-1.35; *I*^2^=71.4%; τ^2^=0.2002), with a 95% prediction interval from 0.14 to 3.46. For hospitalization or readmission, 3 studies including 963 participants yielded a pooled RR of 0.85 (95% CI 0.52-1.40; *I*^2^=42.9%; τ^2^=0.0132), with a 95% prediction interval from 0.31 to 2.39.

#### Results of Investigations of Heterogeneity

Exploratory subgroup analyses by follow-up duration were feasible only for HbA_1c_, SBP, and DBP. Detailed subgroup forest plots are provided in Multimedia Appendix 7. No statistically significant subgroup differences were observed between shorter follow-up (≤6 months) and longer follow-up (12 months) studies for HbA_1c_ (*P*=.18), SBP (*P*=.90), or DBP (*P*=.30). For HbA_1c_, the pooled MD was −0.29 percentage points (95% CI −0.97 to 0.38) in the shorter follow-up subgroup (3 studies) and −0.05 percentage points (95% CI −0.45 to 0.36) in the longer follow-up subgroup (3 studies). For SBP, the corresponding pooled MDs were −2.95 mm Hg (95% CI −20.81 to 14.92) and −3.55 mm Hg (95% CI −13.89 to 6.79). For DBP, they were 0.21 mm Hg (95% CI −5.16 to 5.57) and −1.40 mm Hg (95% CI −5.26 to 2.46).

These subgroup findings were interpreted cautiously because follow-up duration was closely aligned with intervention orientation in several comparisons, and the small number of studies within each subgroup limited the ability to disentangle follow-up duration from intervention type or delivery orientation. For the remaining pooled outcomes, the number and composition of contributing studies did not support reliable formal subgroup comparisons.

#### Results of Sensitivity Analyses

Leave-one-out analyses showed that the interpretation of the pooled results was generally stable for HbA_1c_, SBP, DBP, mortality, hospitalization or readmission, pain-related functional impact or disability, and depression response. For these outcomes, exclusion of any single study did not materially change the direction or statistical interpretation of the pooled estimates, although effect sizes and between-study heterogeneity varied across reanalyses.

For depression severity, the pooled effect remained in favor of the intervention after omission of each individual study, with SMDs ranging from −0.41 to −0.57; however, substantial between-study heterogeneity persisted. Sensitivity findings were less stable for HRQoL outcomes because of sparse evidence. Mental HRQoL was based on only 2 studies, so leave-one-out analyses reduced the synthesis to single-study estimates. For physical HRQoL, omission of one study shifted the pooled estimate to a small effect favoring control, whereas other reanalyses remained compatible with little or no effect. Overall, sensitivity analyses supported the robustness of the main interpretation for most pooled outcomes, but stability was limited for outcomes supported by very few studies. Detailed leave-one-out results are provided in Multimedia Appendix 8.

#### Narrative Synthesis of Additional Outcomes and Study-Level Findings Not Used as Main Pooled Estimates

Additional outcomes, alternative time points, trial-reported analyses, and study-level findings not included in the main pooled estimates are summarized in Multimedia Appendix 6. Overall, these findings were mixed and fragmented across outcome domains.

In the cardiometabolic domain, some individual trials reported favorable findings. For example, in Joint Asia Diabetes Evaluation (JADE) [36], the team-based empowered care group achieved at least 3 treatment targets more often than usual care and empowered care alone (44.6% vs 38.2% and 35.7%; RR 1.17 and 1.25, respectively), with favorable trial-reported changes in HbA_1c_ and low-density lipoprotein cholesterol. Other trials, however, reported no clear incremental benefit for related glycemic, blood pressure, lipid, or broader cardiometabolic risk markers.

Patient-reported, symptom-related, and functional outcomes were similarly heterogeneous. Bernocchi et al [56] reported improved exercise tolerance after telerehabilitation, with a 6-minute walk distance change of +60 m in the intervention group versus −15 m in controls (*P*=.004), alongside improvements in dyspnea, physical activity, disability, and disease-specific quality of life. Rollman et al [40] found that blended collaborative care improved mental HRQoL compared with usual care (adjusted Mental Component Summary of the 12-Item Short Form Health Survey difference 4.47, 95% CI 1.65-7.28; *P*=.002), but not compared with heart failure–only enhanced usual care (difference 1.12, 95% CI −1.15 to 3.40; *P*=.33). Yu et al [37] reported improved chronic illness care experience (Patient Assessment of Chronic Illness Care difference 0.71, 95% CI 0.38-1.04), without clear effects on decisional conflict, diabetes distress, or overall quality of life. Other favorable findings for symptom, functional, self-management, and care-process outcomes were generally isolated and not consistent across studies or related outcomes.

Event-based and use outcomes also showed inconsistent findings. Lear et al [64] reported a lower composite of hospitalization or death (31.9% vs 45.1%; odds ratio 0.57, 95% CI 0.33-0.98) and delayed time to first hospitalization (hazard ratio [HR] 0.62, 95% CI 0.39-0.97). Yao et al [31] reported lower risks of the composite adverse outcome (HR 0.37, 95% CI 0.26-0.53), rehospitalization (HR 0.42, 95% CI 0.27-0.64), and thromboembolic events (HR 0.17, 95% CI 0.05-0.51). However, other event-based or use outcomes, including drug-related hospital admissions, falls, emergency department visits, readmission-related end points, and broader use outcomes, did not show clear reductions.

Taken together, nonpooled findings suggested possible benefits in selected clinical, patient-reported, functional, self-management, care process, and use domains, but these signals were usually based on single studies, subgroup findings, short-term follow-up, or incompletely harmonized outcome reporting. The narrative evidence therefore remained insufficiently consistent to support firm cross-study conclusions.

#### Implementation Outcomes

Implementation-related outcomes were synthesized descriptively across reach, engagement or adherence, and feasibility, with attention to mode of delivery (Multimedia Appendix 9) [31-66]. Reporting was heterogeneous and often incomplete, and the measures and denominators used were frequently not directly comparable.

Explicit reach denominators were available only in a subset of trials. Where reported, recruitment or participation yield varied substantially. For example, 161 of 297 (54.2%) invited participants enrolled in one telephone-coaching trial [65], 49 of 107 (45.8%) assessed participants were randomized in a digital health coaching study [51], 85 of 1359 (6.3%) screened individuals were randomized in an internet-based cognitive behavioral therapy (CBT) for insomnia trial [47], and 230 of 3438 (6.7%) individuals contacted by invitation letter were randomized in an internet-based chronic disease management trial [64]. In many other studies, only the randomized sample was reported, such as 2393 participants in a multicenter cardiometabolic trial [36], 3698 in a large mobile health trial [32], and 2008 in an inpatient medication-optimization trial [39]; these figures reflected participation volume rather than directly comparable estimates of reach.

Relatively high engagement or adherence was reported in several telemonitoring, mobile health, and blended interventions that incorporated ongoing monitoring, coaching, or clinician contact. Remote-monitoring indicators included use on 69% to 70% of study days [41], mean use of 2.7 blood pressure checks and 1.2 blood glucose checks per week [55], and 8111 alerts generated during tele-homecare, of which 93.2% were vital-sign alerts [57]. In an internet-based chronic disease management platform, participants had a median of 2.5 log-ins per week, 84.5% logged in at least weekly, and 32,095 alerts were generated [64]. Engagement or adherence was also substantial in some clinician-supported or telerehabilitation interventions, including rates of 75.9% and 80.2% in the 2 active JADE arms [36] and exercise participation of 93% in telerehabilitation [56].

By contrast, module completion was more variable in self-guided, low-touch, or module-based psychological and behavioral interventions. Reported completion ranged from 55% of 6 core modules in one guided internet intervention [48] to median completion of 4 of 7 modules in a blended low-intensity program [52], mean completion of 0.29 modules in a self-guided web-based CBT trial [63], and mean completion of 2.0 of 8 modules in a CBT for insomnia trial, in which 31% of participants completed no modules [47]. Device-specific differences were also observed: in one 3-arm eHealth trial, participants assigned to the laptop version used the intervention on more days during months 1 to 4 than those assigned to the smart-display version (mean 28.5 vs 19.0 days; *P*<.001) [43].

Feasibility appeared favorable in many trials with respect to retention, acceptability, or safety, although implementation challenges were also reported. Retention was high in several studies, including 92.1% at 12 months in a home telesurveillance trial [58], 91.4% at 12 months in a telemonitoring study for hypertension and diabetes [55], 96.9% in a WeChat-based education trial [34], 99.5% at 12 months in an inpatient prescribing-optimization trial [39], and 94% completion in a telemedicine-supported exercise trial [61]. Acceptability was also reported positively in some studies, including a mean 8-item Client Satisfaction Questionnaire score of 24.1 with 87% willingness to recommend the program [48], a mean satisfaction score of 22.3 out of 24 in telerehabilitation [56], and 95% satisfaction with 93% willingness to recommend in an internet-delivered CBT trial [50]. Reported barriers included low uptake of proactive telephone coaching, with only 41% of selected participants consenting [66]; electronic medical record export problems resulting in approximately 13% to 18% missing data [38]; drug unavailability despite high follow-up completion [32]; and poorer usability of smart-display delivery because of voice dictation errors and difficulty handling text-heavy functions [43].

Overall, DHIs for multimorbidity appeared feasible in many trial contexts, particularly when embedded within monitored or clinician-supported care pathways, but implementation reporting remained insufficiently standardized for quantitative synthesis.

#### Intervention-Multimorbidity Matching Patterns

Based on inductive thematic analysis of intervention descriptions (Multimedia Appendix 10) [31-66], 4 descriptive categories of DHIs were identified across the 36 included studies: telemonitoring (8/36, 22.2%) [41,45,53-58], web or app-based self-management (9/36, 25%) [31,34,36,43,44,51,61,62,64], telephone or collaborative care (6/36, 16.7%) [33,40,59,60,65,66], and decision-support or psychosocial approaches (13/36, 36.1%) [32,35,37-39,42,46-50,52,63]. Interrater agreement for the final classification was excellent (κ=0.91, 95% CI 0.82-1.00; Multimedia Appendix 10).

**Figure 7.**
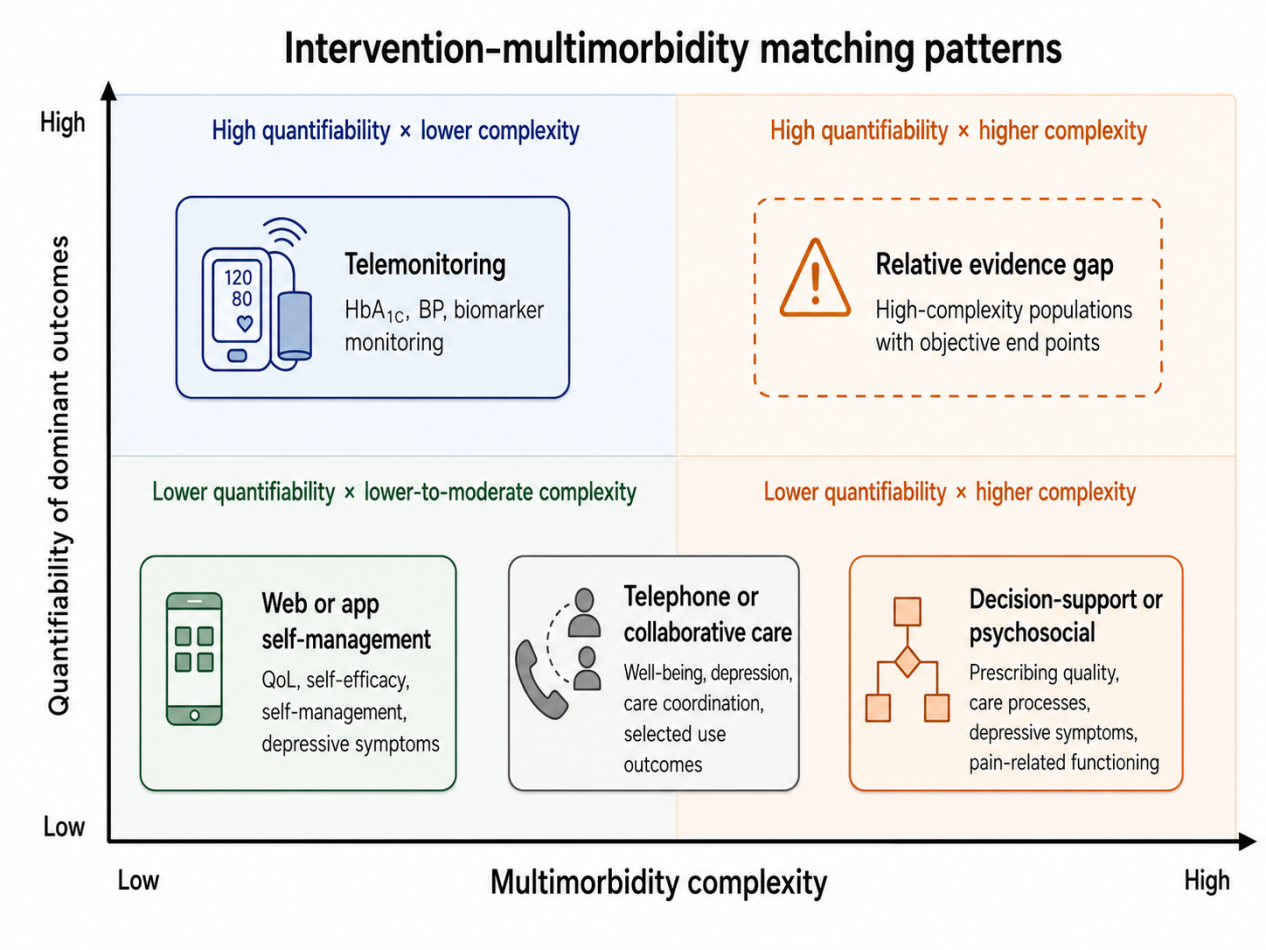
Complexity-quantifiability framework of intervention-multimorbidity matching patterns. The 4 descriptive digital health intervention categories are positioned schematically according to the quantifiability of their dominant outcomes and the complexity of the multimorbidity populations in which they were evaluated. Higher quantifiability refers to objective, repeatedly measurable, or biomarker-based outcomes, whereas lower quantifiability refers mainly to patient-reported or context-dependent constructs. Multimorbidity complexity ranges from relatively bounded cardiometabolic combinations to profiles involving multisystem disease, functional impairment, polypharmacy, psychosocial comorbidity, or care-coordination needs. The upper-right quadrant indicates a relative evidence gap in interventions for clinically complex multimorbidity populations evaluated using objective or repeatedly measurable end points. Placement is interpretive and reflects recurrent patterns across the included studies rather than quantitative thresholds. BP: blood pressure; HbA_1c_: glycated hemoglobin; QoL: quality of life.

To further characterize intervention-multimorbidity fit, we mapped intervention categories onto a 2-axis matrix defined by the quantifiability of dominant outcomes and multimorbidity complexity (Figure 7). This mapping was exploratory and descriptive rather than a formal quantitative scoring of intervention or patient complexity. Outcome quantifiability referred to whether dominant targets could be measured directly, repeatedly, and objectively, such as blood pressure, blood glucose, or weight, as opposed to more context-dependent patient-reported constructs. Multimorbidity complexity referred to the degree of clinical, functional, and care-delivery heterogeneity in the target population, ranging from relatively bounded cardiometabolic combinations to multisystem disease, mental health comorbidity, cognitive or functional impairment, polypharmacy, and care-coordination needs.

Telemonitoring interventions clustered mainly toward the higher-quantifiability and lower-to-moderate complexity region, where trials most often targeted bounded cardiometabolic profiles and objective outcomes such as HbA_1c_, blood pressure, or other biomarkers. Web- or app-based self-management interventions clustered mainly in the lower-quantifiability and lower-to-moderate complexity region, with greater emphasis on patient-reported or behavior-related outcomes, including quality of life, self-efficacy, self-management, and depressive symptoms. Telephone or collaborative care occupied an intermediate position and was more often applied to populations with moderate-to-higher complexity, with outcomes related to well-being, depression, care coordination, and selected use end points.

Decision-support or psychosocial approaches clustered predominantly in the lower-quantifiability and higher-complexity region, where interventions more commonly targeted prescribing quality, care processes, depressive symptoms, and pain-related functioning. This category was heterogeneous, including both clinical decision-support tools and structured psychological interventions, and should therefore be interpreted as a descriptive grouping rather than a fixed taxonomy. The higher-quantifiability and higher-complexity quadrant was sparsely represented, indicating a relative evidence gap in digitally enabled interventions for clinically complex multimorbidity populations evaluated using objective or repeatedly measurable end points.

### Reporting Biases

No formal assessment of reporting biases due to missing results could be undertaken at the synthesis level because no meta-analysis included at least 10 studies. Funnel plots and Egger regression had been prespecified for meta-analyses including at least 10 studies, consistent with methodological guidance that these methods are difficult to interpret when only a small number of studies are available [28]. Accordingly, funnel plots and Egger regression were not performed for any meta-analysis.

At the study level, possible selective nonreporting was considered during risk-of-bias assessment, including comparison of published outcomes with trial registrations, study protocols, or supplementary materials when available. However, because each synthesis included only a small number of studies, reporting biases due to missing results could not be formally assessed across studies.

### Certainty of Evidence

According to the GRADE approach, certainty of evidence was assessed for the 7 outcomes included in the summary of findings table: HbA_1c_, SBP, DBP, depression severity, all-cause mortality, hospitalization or readmission, and physical HRQoL (Table 1). Certainty ranged from low to very low across these outcomes. Evidence was rated as low certainty for hospitalization or readmission and physical HRQoL and very low certainty for HbA_1c_, SBP, DBP, depression severity, and all-cause mortality. Across the GRADE-assessed outcomes, downgrading was most commonly driven by study limitations, inconsistency, and imprecision. Study limitations reflected risk-of-bias concerns in several contributing trials, particularly concerns related to deviations from intended interventions and missing outcome data. Inconsistency reflected heterogeneity in the magnitude and direction of study-level effects, τ^2^ estimates, and prediction intervals. Imprecision was most evident when CIs crossed the line of no effect, when prediction intervals were wide, or when estimates were based on few studies, few participants, or sparse events. Overall, these assessments indicate limited confidence in the pooled effect estimates for the outcomes assessed using GRADE and suggest that the true effects may differ substantially from the observed summary estimates.

**Table 1.** Summary of findings and certainty of evidence assessed using GRADEpro Guideline Development Tool (GRADEpro GDT)^a^.

Certainty assessment							Patients		Effect		Certainty	Importance
Studies, n	Study design	Risk of bias	Inconsistency	Indirectness	Imprecision	Other considerations	Digital health interventions, n	Control, n	Relative (95% CI)	Absolute (95% CI)		
Glycated hemoglobin (assessed with: glycated hemoglobin (%))												
6	Randomized trials	Serious^b^	Serious^c^	Not serious	Serious^d^	None	2011	1981	—^e^	MD^f^ 0.12 lower (0.36 lower to 0.11 higher)	⨁◯◯◯ Very low^b^^,^^c^^,d^	Important
Systolic blood pressure (assessed with: systolic blood pressure (mm Hg))												
6	Randomized trials	Serious^b^	Very serious^g^	Not serious	Serious^d^	None	2774	2822	—	MD 3.4 lower (8.94 lower to 2.14 higher)	⨁◯◯◯ Very low^b^^,d,g^	Important
Diastolic blood pressure (assessed with: diastolic blood pressure (mm Hg))												
6	Randomized trials	Serious^b^	Serious^c^	Not serious	Serious^d^	None	2724	2760	—	MD 0.86 lower (2.8 lower to 1.08 higher)	⨁◯◯◯ Very low^b^^,^^c^^,d^	Important
Depression severity (assessed with: depression symptom severity scales)												
8	Randomized trials	Serious^b^	Very serious^g^	Not serious	Not serious	None	903	958	—	SMD^h^ 0.49 SD lower (0.82 lower to 0.16 lower)	⨁◯◯◯ Very low^b^^,^^g^	Critical
Mortality, all-cause (assessed with: all-cause mortality)												
5	Randomized trials	Serious^b^	Serious^c^	Not serious	Serious^d^	None	230/2263 (10.2%)^i^	293/2441 (12%)^i^	RR^j^ 0.68 (0.34-1.35)	38 fewer per 1000 (from 79 fewer to 42 more)	⨁◯◯◯ Very low^b^^,^^c^^,^^d^	Critical
Hospitalization or readmission (assessed with: hospitalization or readmission)												
3	Randomized trials	Serious^b^	Not serious	Not serious	Serious^d^	None	184/483 (38.1%)^i^	217/480 (45.2%)^i^	RR 0.85 (0.52-1.40)	68 fewer per 1000 (from 217 fewer to 181 more)	⨁⨁◯◯ Low^b^^,d^	Critical
Physical HRQoL (assessed with: physical health-related quality of life scales)												
3	Randomized trials	Not serious	Not serious	Not serious	Very serious^k^	None	381	432	—	SMD 0.08 SD higher (0.14 lower to 0.3 higher)	⨁⨁◯◯ Low^k^	Critical

a Question: Digital health interventions compared with comparators without the same digital component for adults with multimorbidity. This table was generated using GRADEpro GDT. Certainty of evidence was assessed using GRADE. Prediction intervals were considered when judging inconsistency and imprecision where applicable. This table presents 7 key outcomes selected for the paper.

b Downgraded 1 level because the contributing studies were judged as having some concerns or high risk of bias.

c Downgraded 1 level because effect estimates varied across studies and the prediction interval included both potential benefit and little or no benefit.

d Downgraded 1 level because the 95% CI crossed the line of no effect and included both potential benefit and little or no effect.

e Not applicable.

f MD: mean difference.

g Downgraded 2 levels because heterogeneity was substantial and the prediction interval was wide, including both potentially important benefit and little or no benefit.

h SMD: standardized mean difference.

i Standardized dichotomous outcomes are reported as n/N (%).

j RR: risk ratio.

k Downgraded 2 levels because the evidence was based on few studies with a limited sample size and the 95% CI crossed the line of no effect.

## Discussion

### Principal Findings

In this systematic review and meta-analysis of randomized trials, DHIs for adults with multimorbidity showed limited and uncertain evidence of consistent benefit across clinical, patient-reported, and use outcomes. Most pooled estimates were compatible with little or no effect. Depression severity was the only pooled outcome whose 95% CI favored the intervention, but the prediction interval spanned benefit to harm, and certainty was very low. Implementation findings suggested that many DHIs were feasible in trial contexts, particularly when supported by monitoring, coaching, or clinician contact, although reporting of reach, engagement or adherence, feasibility, fidelity, and maintenance was heterogeneous and often incomplete. Overall, these findings suggest that the value of DHIs in multimorbidity depends less on technology type alone than on the alignment among intervention mechanism, patient complexity, outcome architecture, and delivery context.

### Limited Transferability of Condition-Specific DHI Evidence to Multimorbidity

The generally null pooled effects in this review contrast with evidence from disease-specific digital health research. Recent systematic reviews of randomized trials in type 2 diabetes and hypertension have shown that DHIs can improve selected cardiometabolic outcomes, particularly when monitoring, feedback, and treatment adjustment are closely linked [8,9]. However, the lack of consistent benefits among adults with multimorbidity suggests that effects demonstrated in single-condition populations may not be straightforwardly extrapolated to populations managing multiple interacting conditions, treatments, symptoms, and care pathways.

One explanation is that multimorbidity changes the context in which DHIs are expected to work. In single-condition care, DHIs often operate within relatively bounded clinical targets and clearer response pathways. In multimorbidity, the same functions are introduced into a care ecology shaped by treatment burden, polypharmacy, fragmented care, and competing self-management priorities [1,7,67,68]. Digital tools that add monitoring tasks, alerts, or platform interactions without reducing existing workload may dilute engagement or increase burden [69,70]. Moreover, single-condition DHIs often build on established clinical guidelines, treatment targets, and escalation rules, whereas multimorbidity care requires balancing multiple diseases and management goals in the absence of equally mature integrated care pathways [1,71,72]. This may help explain why monitoring, education, or reminder functions that are effective in single-condition care have weaker or less consistent effects when transferred to multimorbidity.

Variations across individual trials further support this interpretation and may reflect differences in how DHIs were integrated into care delivery. The JADE trial evaluated a risk-stratified, team-based model that linked digital reporting with structured assessment and treatment adjustment and reported favorable cardiometabolic findings [36]. Similarly, the Mobile Health Technology for Improved Screening and Optimized Integrated Care in Atrial Fibrillation trial embedded a mobile health–supported ABC pathway within integrated atrial fibrillation care and reported favorable clinical end points [31]. By contrast, point-of-care digital decision support in mWellcare did not show incremental benefit over enhanced usual care [32]. These examples do not establish that integrated digital care models are consistently effective, but they suggest that digital information may be more likely to matter when it is embedded within coordinated clinical pathways and connected to actionable care [20,21].

In this review, depression severity was the only outcome whose pooled 95% CI favored the intervention. Although certainty was very low and the prediction interval spanned benefit to harm, this finding is consistent with evidence that digitally delivered psychological interventions can reduce depressive symptoms in some populations [73,74]. Within the included psychosocial DHI studies, depression was often a direct therapeutic target and may therefore be more closely aligned with intervention components such as symptom monitoring, feedback, behavioral activation, and cognitive-behavioral strategies [46,48,52]. Digital platforms may also improve access to psychological support and reinforce therapeutic skills over time [73,74]. However, any apparent benefit is likely to depend on intervention intensity, human support, participant engagement, and baseline symptom severity [46,48,52]. This finding suggests that DHIs may be more effective for outcomes closely linked to their therapeutic components than for broader outcomes influenced by multiple factors.

The weakest signals were observed for broader outcomes such as quality of life, hospitalization, and mortality. These outcomes are highly relevant to multimorbidity care, but they are generated by multiple interacting processes beyond the direct reach of many DHIs [7,21,75,76]. Quality of life may reflect function, psychological distress, social participation, and patient experience, whereas hospitalization and mortality may be shaped by frailty, disease severity, care coordination, social vulnerability, and local service organization [21,77]. These outcomes may therefore require interventions that go beyond monitoring or stand-alone self-management support to address care coordination, clinical decision-making, and system-level responsiveness.

This creates a central tension for digital health in multimorbidity. The populations with the greatest need for longitudinal, coordinated, and data-informed support may also be the least well served by simple, disease-specific, or low-touch digital models. The implication is not that DHIs are unsuitable for multimorbidity, but that their value is likely to depend on whether they are designed as integrated, burden-sensitive, and clinically actionable components of care rather than as generic digital extensions of single-disease management.

### Implementation as the Missing Link Between Digital Use and Clinical Benefit

The implementation findings provided a more nuanced picture than pooled effectiveness estimates alone. Across the included trials, many DHIs appeared feasible in trial contexts, particularly when embedded within monitored, coached, or clinician-supported care pathways. However, feasibility, retention, acceptability, and safety indicators mainly show that an intervention can be delivered; they do not necessarily show that it has changed behavior, treatment decisions, or care processes. Implementation reporting was also heterogeneous and often incomplete, with reach, adherence, feasibility, and fidelity measured using noncomparable indicators and denominators. These findings suggest that implementation is not peripheral to effectiveness but part of the pathway through which digital interventions do or do not translate into population benefit [14,20,21].

A central implementation issue is whether digital use is converted into meaningful action. Patients may access a platform, upload data, complete monitoring tasks, or remain in follow-up, but these forms of use are unlikely to produce clinical benefit unless digital information is interpreted and translated into behavioral adjustment, treatment decisions, care coordination, or problem-solving within the wider care system [20,21]. This conversion pathway may be especially fragile in multimorbidity, where care is distributed across multiple conditions, providers, self-management tasks, and decision points [7,67,68]. Trials should therefore report not only whether an intervention was used, but also whether and how use generated actionable responses by patients, caregivers, clinicians, or care teams.

Adherence should be viewed not merely as a technical metric but as an indicator of intervention exposure and mechanism activation. Telemonitoring, mobile health, and blended interventions with ongoing monitoring, feedback, coaching, or clinician contact generally reported stronger adherence-related indicators, whereas engagement was more variable in self-guided or low-touch psychological and behavioral interventions [14,20,21]. Interpretation remains challenging because log-ins, monitoring frequency, session attendance, module completion, retention, and follow-up completion capture different forms of exposure [20,21,78]. Nevertheless, inadequate or unsustained engagement may prevent intended mechanisms from operating, whether those mechanisms involve behavioral change, clinical response, or care coordination [20,21,78]. Future studies should define the intended intervention dose, report adherence trajectories, and clarify how sustained use is expected to generate benefit, particularly among populations facing high treatment and digital health burden [67,69].

Workflow integration is another key condition for implementation to influence outcomes. A DHI that remains an external add-on may generate data without changing care if those data do not enter clinical workflows, are not visible to relevant care-team members, or are not linked to accountable decisions [79-81]. This issue is particularly salient in multimorbidity, where care is often distributed across repeated contacts, multiple clinicians, and competing priorities [77,82]. The same reporting gaps made it difficult to distinguish among limited intervention efficacy, inadequate exposure, weak fidelity, poor workflow integration, and mismatch between intervention target and evaluated outcome [20,21,78]. Multimorbidity DHI trials should therefore treat implementation reporting as a core design requirement, with clearer links among reach, adherence, fidelity, maintenance, digital outputs, and clinical action [21,78].

Sustainment is a further challenge. Short-term use, acceptability, or retention during a funded trial does not necessarily mean that an intervention can be maintained once active implementation support is withdrawn [14,81]. Sustained implementation requires stable workforce roles, financing, interoperability, clinical accountability, and routines through which digital information is acted on. This challenge may be particularly consequential in low- and middle-income settings, where the limited number of published multimorbidity-focused DHI trials may reflect infrastructure constraints as well as fragmented primary care systems, workforce and information-system limitations, and funding architectures historically oriented toward disease-specific programs [83-85]. However, our supplementary search of trial registries and protocols identified a growing pipeline of multimorbidity-focused digital programs across diverse settings, including low- and middle-income contexts, suggesting increasing interest in more integrated and implementation-conscious models of care (Multimedia Appendix 11). Thus, the implementation question is not only whether a digital tool can be deployed, but whether the surrounding system has sufficient capacity, incentives, and care pathways to embed, act on, and sustain it.

Finally, implementation should be viewed as an equity question as much as an operational one [69,86,87]. The goal is not only to maximize average feasibility or adherence, but also to determine who is reached, who remains engaged, and who benefits over time [69,86]. Evidence from low- and middle-income settings and underserved groups remained limited, relative to the global burden of multimorbidity, and reporting by socioeconomic position, digital literacy, rurality, language, disability, or other equity-relevant characteristics was sparse. This does not mean that multimorbidity DHIs are inherently inequitable, but it does mean that equity cannot be inferred from average feasibility or acceptability alone [86,87]. Without deliberate attention to reach, support needs, workflow fit, and sustainment, digital health may continue to benefit primarily those patients and systems already best positioned to engage with it [69,87].

### Intervention-Multimorbidity Matching Patterns and Implications

The complexity-quantifiability framework synthesizes the preceding findings into an exploratory tool for interpreting and designing multimorbidity DHI trials. The framework positions DHI categories according to the quantifiability of dominant outcomes and the complexity of the multimorbidity populations in which they were evaluated. Its purpose is not to classify technologies by type alone, but to assess whether the intervention mechanism is aligned with the target population, outcome domain, and implementation context. This focus responds to a recurring challenge in multimorbidity DHI research: prior reviews have reported substantial heterogeneity in intervention models, mechanisms, outcomes, and implementation barriers, making it difficult to interpret DHIs as a single intervention class [10,11]. Consistent with complex intervention guidance, effects should therefore be interpreted in relation to mechanisms of action, context, and outcome choice rather than technology type alone [20,21].

In this framework, quantifiability refers to evaluability rather than clinical importance. Outcomes such as HbA_1c_ and blood pressure are easier to standardize and lie closer to monitoring, feedback, and treatment-adjustment pathways through which many DHIs operate [8,9]. By contrast, outcomes such as treatment burden, care coordination, patient experience, and psychosocial functioning are highly relevant to multimorbidity care but are often more context-dependent and less consistently measured across trials [7,75,88]. This creates an evaluative asymmetry: biomarker-oriented interventions may be easier to detect and pool, whereas benefits in patient-important domains may be clinically meaningful but analytically harder to capture. Weaker evidence in these domains may therefore partly reflect weaker evaluability rather than lower clinical relevance [75,88].

The complexity dimension adds a second layer of interpretation. In relatively bounded cardiometabolic populations, the intervention mechanism, clinical target, and outcome metric can often be specified with greater precision. In more complex multimorbidity populations, effects may be distributed across interacting domains such as symptoms, function, medication burden, and care coordination [1,67]. This does not make DHIs less relevant for complex multimorbidity; rather, it means that trials need to specify which part of complexity the intervention is intended to address. For example, a medication-optimization DHI should be evaluated in relation to prescribing quality, treatment burden, decision-making, and downstream service use, whereas a psychosocial DHI should be assessed according to whether its therapeutic mechanism is aligned with depressive symptoms, coping, pain interference, functional participation, or quality of life [20,21].

The relative sparsity of evidence in the high-complexity, high-quantifiability quadrant is informative but should be interpreted descriptively. Within the included randomized evidence, this quadrant was less populated, suggesting that current trials have not yet fully addressed clinically complex multimorbidity populations using objective, repeatable, and clinically actionable end points. This gap likely reflects both design and methodological challenges. Trials in highly complex populations may face recruitment, retention, safety, and external-validity challenges, and may exclude patients with severe frailty, cognitive impairment, unstable illness, or extensive comorbidity [21]. At the same time, many existing DHIs may not be sufficiently multicomponent, workflow-integrated, or responsive to competing clinical priorities to influence objective outcomes in these populations [20,21]. As a result, clinically complex patients may remain underrepresented in biomarker-oriented DHI trials despite being among those who could benefit most from integrated, data-informed support.

Future multimorbidity DHI research should therefore move beyond broad technology labels and define the intended fit among intervention mechanism, patient complexity, outcome architecture, implementation strategy, and follow-up horizon. For lower-complexity cardiometabolic populations, objective physiological end points may be appropriate when digital monitoring is linked to timely feedback and clinical response. For higher-complexity populations, trials may need to combine objective markers with patient-prioritized outcomes, treatment burden, care coordination measures, and implementation end points [20,21,75]. In this sense, the complexity-quantifiability framework should be viewed not as a validated taxonomy, but as a pragmatic, hypothesis-generating tool for trial design, interpretation, and evidence synthesis.

### Strengths and Limitations

This review has several strengths. It provides an updated synthesis of randomized evidence on DHIs for adults with multimorbidity, a population often underrepresented in single-condition digital health reviews. By jointly examining effectiveness, implementation outcomes, and intervention-multimorbidity matching patterns, this review extends prior work beyond pooled effects alone and offers a more clinically interpretable account of why digital interventions may or may not generate benefit in multimorbidity. The broad search strategy, restriction of quantitative synthesis to randomized and cluster-randomized trials, and explicit attention to implementation and intervention-outcome alignment strengthened the relevance of the findings for both trial interpretation and future study design.

Several limitations should also be considered. First, by prioritizing randomized and cluster-randomized trials, this review may have excluded pragmatic, observational, or implementation-focused studies that could better inform sustainability, scalability, and external validity in routine care. Second, most meta-analyses included only a small number of studies, and substantial heterogeneity remained across multimorbidity profiles, intervention models, comparators, outcome definitions, and follow-up periods. Consequently, several pooled estimates were imprecise, prediction intervals were wide, and subgroup findings should be interpreted cautiously. Third, the evidence base was concentrated largely in high-income settings and enrolled unevenly represented populations, limiting generalizability to health systems with different service structures, digital infrastructures, and care needs. Fourth, implementation reporting was often incomplete or nonstandardized, particularly for reach, adherence, fidelity, and maintenance, making it difficult to distinguish limited intervention efficacy from inadequate exposure, suboptimal delivery, or contextual mismatch. Finally, many patient-important outcomes and noncardiometabolic biomarkers relevant to multimorbidity were reported too inconsistently or sparsely to support reliable cumulative synthesis.

### Conclusions

This systematic review and meta-analysis extends prior reviews that focused mainly on telemedicine or descriptively mapped digital tools by integrating randomized evidence across multiple outcome domains, GRADE certainty ratings, prediction intervals, implementation findings, and an intervention-multimorbidity matching perspective. This integrated approach addresses 3 key questions for DHIs in multimorbidity: whether they are effective, how they are implemented, and under what conditions they are most likely to be useful. Overall, pooled effects did not show consistent or transferable benefits across most major outcome domains. Depression severity was the only outcome whose pooled 95% CI favored the intervention, but its prediction interval spanned benefit to harm, and certainty was very low. Many DHIs appeared feasible in trial contexts, particularly when supported by monitoring, coaching, or clinician contact, but favorable feasibility or adherence indicators did not consistently translate into measurable clinical benefit.

The main contribution of this review is to show that the effectiveness of DHIs in multimorbidity should not be judged by technology type alone. The complexity-quantifiability framework suggests that real-world value depends on the alignment among intervention mechanism, patient complexity, outcome architecture, and delivery context. These conclusions should be interpreted cautiously, given the small number of studies contributing to most pooled outcomes, wide prediction intervals, substantial heterogeneity, low-to-very-low certainty of evidence, and incomplete implementation reporting. Future DHIs for multimorbidity should move beyond generic monitoring, reminders, or stand-alone self-management tools toward adaptive, burden-sensitive, and workflow-integrated models that link digital data to patient priorities, clinician response, and care coordination, while explicitly evaluating who is reached, who remains engaged, and whether benefits are sustained in routine care.

## Supplementary material

10.2196/90458 Multimedia Appendix 1 Amendments to and deviations from the registered PROSPERO protocol.

10.2196/90458 Multimedia Appendix 2 Eligibility criteria for study inclusion.

10.2196/90458 Multimedia Appendix 3 Search strings.

10.2196/90458 Multimedia Appendix 4 Characteristics of included studies.

10.2196/90458 Multimedia Appendix 5 Risk of bias of included studies assessed using Risk of Bias tool for randomized trials, version 2 for the main outcome of each study.

10.2196/90458 Multimedia Appendix 6 Study-level results for additional outcomes, alternative time points, and trial-reported analyses not used as main pooled effect estimates.

10.2196/90458 Multimedia Appendix 7 Exploratory subgroup analyses.

10.2196/90458 Multimedia Appendix 8 Leave-one-out sensitivity analyses.

10.2196/90458 Multimedia Appendix 9 Study-level implementation data for mode of delivery, reach, adherence, and feasibility.

10.2196/90458 Multimedia Appendix 10 Coding framework used to classify digital health interventions.

10.2196/90458 Multimedia Appendix 11 Ongoing and planned digital health trials and implementation projects for multimorbidity.

10.2196/90458 Checklist 1 PRISMA 2020 main checklist.

10.2196/90458 Checklist 2 PRISMA-S checklist.

## Abbreviations

CBT: cognitive behavioral therapy
DBP: diastolic blood pressure
DHI: digital health intervention
GRADE: Grading of Recommendations Assessment, Development and Evaluation
HbA_1c_: glycated hemoglobin
HR: hazard ratio
HRQoL: health-related quality of life
JADE: Joint Asia Diabetes Evaluation
MD: mean difference
PRESS: Peer Review of Electronic Search Strategies
PRISMA: Preferred Reporting Items for Systematic Reviews and Meta-Analyses
PRISMA-S: PRISMA Extension for Reporting Literature Searches in Systematic Reviews
RCT: randomized controlled trial
RoB 2: Risk of Bias tool for randomized trials, version 2
RR: risk ratio
SBP: systolic blood pressure
SMD: standardized mean difference
