# Supplementary material for: Effectiveness and Implementation of Digital Health Interventions on Physiological, Psychological, and Functional Outcomes in Adults With Multimorbidity: Systematic Review and Meta-Analysis of Randomized Controlled Trials
Source: J Med Internet Res. 2026 Jul 28;28:e90458. doi: 10.2196/90458 (PMC13412019; doi:10.2196/90458)
Supplement: Multimedia Appendix 1 [file jmir-v28-e90458-s001.docx]

**Table S1.** Amendments to and deviations from the registered PROSPERO protocol (CRD420251067068)

| **Domain** | **Registered protocol** | **Final review approach** | **Type** | **Rationale and stage** |
| --- | --- | --- | --- | --- |
| Planned synthesis model | Original protocol planned a network meta-analysis; the revised PROSPERO record states that this was changed because most included studies compared only 2 groups. | Pairwise meta-analysis was conducted for outcomes with sufficiently comparable data. | Registered amendment | The trial network was too sparse and disconnected for network meta-analysis. Implemented before quantitative synthesis. |
| Implementation outcomes | The revised PROSPERO record states that reach, uptake, and feasibility were added because they had not been clearly specified previously. | Reach, uptake, engagement/adherence, and feasibility were extracted when reported and synthesized narratively. | Registered amendment | Implementation was relevant to the review aims, but reporting was too heterogeneous for quantitative pooling. Implemented before final data extraction. |
| Certainty assessment | The PROSPERO record stated that certainty of findings would not be assessed. | Certainty of evidence was assessed using GRADE for the main pooled outcomes. | Deviation | GRADE was added during peer review and revision to improve interpretability and align the review with current reporting expectations. Implemented during revision. |
| Author contact / missing data | The PROSPERO record stated that study authors would be contacted for missing or unclear data where required. | No additional studies or unpublished outcome data were sought through direct contact with study authors or organizations; the review relied on published reports, supplementary materials, trial registrations, and protocols where available. | Deviation | The final analyses were completed using available sources, and no author-contact round was undertaken. Implemented during data extraction and synthesis. |
| Supplementary search during revision | The PROSPERO record indicated that only published studies would be sought. | The main searches used for study selection and quantitative synthesis remained those completed up to April 6, 2026. During revision, a targeted supplementary search of protocol and registry sources was conducted on March 15, 2026, for contextual interpretation only. | Deviation / extension | This supplementary search was undertaken in response to peer-review requests while preserving the original evidence base for the main review and meta-analysis. Implemented during revision. |

**Abbreviation:** PROSPERO, International Prospective Register of Systematic Reviews.
