# Supplementary material for: Effectiveness and Implementation of Digital Health Interventions on Physiological, Psychological, and Functional Outcomes in Adults With Multimorbidity: Systematic Review and Meta-Analysis of Randomized Controlled Trials
Source: J Med Internet Res. 2026 Jul 28;28:e90458. doi: 10.2196/90458 (PMC13412019; doi:10.2196/90458)
Supplement: Multimedia Appendix 2 [file jmir-v28-e90458-s002.docx]

**Eligibility criteria for study inclusion**

Eligibility criteria were defined a priori using the PICOS framework (Population, Intervention, Comparator, Outcomes, and Study design).

**Inclusion criteria**

Studies were eligible for inclusion if they met **all** of the following criteria:

**1. Population**
Adults aged **18 years or older with multimorbidity**, defined as the coexistence of **2 or more chronic health conditions**, typically including at least 1 chronic physical condition. Eligible combinations could include physical–physical or physical–mental comorbidity (eg, diabetes, hypertension, cardiovascular disease, chronic obstructive pulmonary disease, chronic kidney disease, depression, or arthritis). For studies including mixed populations, data had to be reported separately for the eligible multimorbidity subgroup to allow independent extraction.

**2. Intervention**
Any **digital health intervention (DHI)** intended to support the management of adults with multimorbidity. Eligible interventions included, but were not limited to:
(1) mobile health applications (mHealth);
(2) telemonitoring or home telehealth;
(3) web-based platforms or portals;
(4) digital self-management tools;
(5) AI-driven decision support systems;
(6) SMS- or email-based behavior change programs; and
(7) clinical decision support systems.

To be eligible, the digital component had to constitute a **core therapeutic, monitoring, self-management, or care-coordination element** of the intervention, rather than serving only as an adjunct for follow-up, administrative communication, reminder delivery, or passive information provision.

**3. Comparator**
Usual care, enhanced usual care, non-digital interventions, or other active comparators **without the same digital intervention component**.

**4. Outcomes**
Studies reporting at least **1 clinical, behavioral, patient-reported, or healthcare utilization outcome** relevant to multimorbidity management, including but not limited to:
(1) clinical outcomes (eg, blood pressure, glycemic control, hospital admissions, mortality);
(2) behavioral outcomes (eg, medication adherence, self-management);
(3) patient-reported outcomes (eg, quality of life, depressive symptoms); and
(4) healthcare utilization outcomes (eg, readmissions, emergency visits, healthcare costs).

**5. Study design**
(1) randomized controlled trials (RCTs) or cluster RCTs; and
(2) published in English.

**Exclusion criteria**

Studies were excluded if they:

(1) were **not primary reports of randomized trials,** including protocols, editorials, commentaries, conference abstracts without full reports, narrative reviews, cross-sectional studies, retrospective studies, qualitative studies, or secondary analyses that did not provide independently usable outcome data;

(2) were **pilot, feasibility, acceptability, usability, or proof-of-concept trials** whose primary purpose was not to evaluate intervention effectiveness on eligible patient outcomes;

(3) included **duplicate or overlapping data** from another included report, in which case only the most complete and relevant publication was retained; or

(4) evaluated interventions that addressed only **a single index disease** without a clear focus on multimorbidity management, even if some participants had comorbid conditions.
