# Supplementary material for: Effectiveness and Implementation of Digital Health Interventions on Physiological, Psychological, and Functional Outcomes in Adults With Multimorbidity: Systematic Review and Meta-Analysis of Randomized Controlled Trials
Source: J Med Internet Res. 2026 Jul 28;28:e90458. doi: 10.2196/90458 (PMC13412019; doi:10.2196/90458)
Supplement: Multimedia Appendix 3 [file jmir-v28-e90458-s003.docx]

**1. PubMed**

Database: PubMed (National Library of Medicine)

Platform: PubMed.gov

Date last searched: April 6, 2026

Records retrieved: 1350

| Search | Query | Items found |
| --- | --- | --- |
| #1 | "Multimorbidity"[Mesh] OR "Comorbidity"[Mesh] OR "Multiple Chronic Conditions"[Mesh] | 138,926 |
| #2 | multimorbid*[tiab] OR "multiple morbidity"[tiab] OR "multiple long-term condition*"[tiab] OR "multiple chronic condition*"[tiab] OR "multiple chronic disease*"[tiab] OR "multiple chronic illness*"[tiab] OR ((coexist*[tiab] OR co-occur*[tiab] OR cooccur*[tiab] OR concurrent*[tiab] OR comorbid*[tiab]) AND (chronic[tiab] OR condition*[tiab] OR disease*[tiab] OR illness*[tiab])) | 359,769 |
| #3 | #1 OR #2 | 452,899 |
| #4 | "Digital Health"[Mesh] OR "Telemedicine"[Mesh] OR "Remote Patient Monitoring"[Mesh] OR "Remote Consultation"[Mesh] OR "Mobile Applications"[Mesh] OR "Text Messaging"[Mesh] OR "Wearable Electronic Devices"[Mesh] OR "Telerehabilitation"[Mesh] | 100,010 |
| #5 | ehealth[tiab] OR "e-health"[tiab] OR "digital health"[tiab] OR telemed*[tiab] OR telehealth[tiab] OR telecare[tiab] OR telemonitor*[tiab] OR "remote patient monitoring"[tiab] OR teleconsult*[tiab] OR videoconsult*[tiab] OR "mobile health"[tiab] OR mhealth[tiab] OR "m-health"[tiab] OR "mobile app*"[tiab] OR smartphone*[tiab] OR "text messag*"[tiab] OR sms[tiab] OR "web-based"[tiab] OR "internet-based"[tiab] OR wearable*[tiab] OR telerehabilitation[tiab] OR email*[tiab] OR "patient portal*"[tiab] OR "electronic health record*"[tiab] OR "electronic medical record*"[tiab] OR "personal health record*"[tiab] OR "clinical decision support"[tiab] OR "decision support system*"[tiab] OR "health information technolog*"[tiab] OR "virtual care"[tiab] | 360,542 |
| #6 | #4 OR #5 | 392,681 |
| #7 | #3 AND #6 | 11,347 |
| #8 | randomized controlled trial[pt] OR controlled clinical trial[pt] OR randomized[tiab] OR randomised[tiab] OR placebo[tiab] OR "clinical trials as topic"[mesh:noexp] OR randomly[tiab] OR trial[ti] OR "cluster random*"[tiab] OR "cluster-random*"[tiab] OR "group random*"[tiab] OR "group-random*"[tiab] | 1,845,361 |
| #9 | animals[mh] NOT humans[mh] | 5,440,787 |
| #10 | #7 AND #8 NOT #9 | 1350 |

**2. Web of Science**

Database: Web of Science Core Collection (Science Citation Index Expanded)

Platform: Clarivate Analytics

Date last searched: April 6, 2026

Records retrieved: 635

| Search | Query | Items found |
| --- | --- | --- |
| #1 | TS=(multimorbid* OR "multiple morbidity" OR "multiple long-term condition*" OR "multiple chronic condition*" OR "multiple chronic disease*" OR "multiple chronic illness*" OR ((coexist* OR co-occur* OR cooccur* OR concurrent* OR comorbid*) NEAR/3 (condition* OR disease* OR illness*))) | 81,976 |
| #2 | TS=("digital health" OR ehealth OR "e-health" OR telemed* OR telehealth OR telecare OR telemonitor* OR "remote patient monitoring" OR "remote consultation" OR teleconsult* OR videoconsult* OR "mobile health" OR mhealth OR "m-health" OR "mobile app*" OR smartphone* OR "text messag*" OR sms OR "web-based" OR "internet-based" OR wearable* OR telerehabilitation OR email* OR "patient portal*" OR "electronic health record*" OR "electronic medical record*" OR "personal health record*" OR "clinical decision support" OR "decision support system*" OR "health information technolog*" OR "virtual care") | 499,561 |
| #3 | TS=(randomized OR randomised OR randomly OR placebo OR trial OR "controlled trial*" OR "clinical trial*" OR "controlled clinical trial*" OR "random* allocat*" OR "cluster random*" OR "cluster-random*" OR "group random*" OR "group-random*" OR "stepped wedge" OR "stepped-wedge") | 2,744,532 |
| #4 | #1 AND #2 AND #3 | 635 |

**3. Embase**

Database: Embase

Platform: Embase.com (Elsevier)

Date last searched: April 6, 2026

Records retrieved: 406

| Search | Query | Items found |
| --- | --- | --- |
| #1 | (multimorbid* OR "multiple morbidity" OR "multiple long-term condition*" OR "multiple chronic condition*" OR "multiple chronic disease*" OR "multiple chronic illness*" OR ((coexist* OR co-occur* OR cooccur* OR comorbid*) NEAR/3 ("chronic condition*" OR "chronic disease*" OR "chronic illness*"))):ti,ab,kw | 26,678 |
| #2 | ("digital health" OR ehealth OR "e-health" OR telemed* OR telehealth OR telecare OR telemonitor* OR "remote patient monitoring" OR "remote consultation" OR teleconsult* OR videoconsult* OR "mobile health" OR mhealth OR "m-health" OR "mobile app*" OR smartphone* OR "text messag*" OR sms OR "web-based" OR "internet-based" OR wearable* OR telerehabilitation OR email* OR "patient portal*" OR "electronic health record*" OR "electronic medical record*" OR "personal health record*" OR "clinical decision support" OR "decision support system*" OR "health information technolog*" OR "virtual care"):ti,ab,kw | 540,210 |
| #3 | randomized:ti,ab,kw OR randomised:ti,ab,kw OR randomly:ti,ab,kw OR placebo:ti,ab,kw OR 'controlled trial*':ti,ab,kw OR 'clinical trial*':ti,ab,kw OR 'controlled clinical trial*':ti,ab,kw OR 'random* allocat*':ti,ab,kw OR 'cluster random*':ti,ab,kw OR 'cluster-random*':ti,ab,kw OR 'group random*':ti,ab,kw OR 'group-random*':ti,ab,kw OR 'stepped wedge':ti,ab,kw OR 'stepped-wedge':ti,ab,kw | 2,895,030 |
| #4 | 'multimorbidity'/exp | 15,550 |
| #5 | (#1 OR #4) AND #2 AND #3 | 406 |
| #6 | 'animal'/exp NOT 'human'/exp | 6,480,031 |
| #7 | #5 NOT #6 | 406 |

**4. Cochrane Library**

Database: Cochrane Library (includes Cochrane Database of Systematic Reviews, CENTRAL, etc.)

Platform: Wiley

Date last searched: April 6, 2026

Records retrieved: 980

| Search | Query | Items found |
| --- | --- | --- |
| #1 | MeSH descriptor: [Multimorbidity] explode all trees | 207 |
| #2 | MeSH descriptor: [Multiple Chronic Conditions] explode all trees | 104 |
| #3 | (multimorbid* OR multiple NEXT morbid* OR multiple NEXT long-term NEXT condition* OR multiple NEXT chronic NEXT condition* OR multiple NEXT chronic NEXT disease* OR multiple NEXT chronic NEXT illness* OR ((coexist* OR co-occur* OR coccur* OR comorbid*) NEAR/3 (chronic condition* OR chronic disease* OR chronic illness*))):ti,ab,kw | 8174 |
| #4 | #1 OR #2 OR #3 | 8174 |
| #5 | MeSH descriptor: [Digital Health] explode all trees | 118 |
| #6 | MeSH descriptor: [Telemedicine] explode all trees | 6149 |
| #7 | MeSH descriptor: [Remote Patient Monitoring] explode all trees | 8 |
| #8 | MeSH descriptor: [Remote Consultation] explode all trees | 493 |
| #9 | MeSH descriptor: [Mobile Applications] explode all trees | 2949 |
| #10 | (digital health OR ehealth OR e-health OR telemed* OR telehealth OR telecare OR telemonitor* OR remote NEXT patient NEXT monitor* OR remote NEXT consultation OR teleconsult* OR videoconsult* OR mobile NEXT health OR mhealth OR m-health OR mobile app* OR smartphone* OR text NEXT messag* OR sms OR web-based OR internet-based OR wearable* OR telerehabilitation OR email* OR patient NEXT portal* OR electronic NEXT health NEXT record* OR electronic NEXT medical NEXT record* OR personal NEXT health NEXT record* OR clinical NEXT decision NEXT support OR decision NEXT support NEXT system* OR health NEXT information NEXT technolog* OR virtual NEXT care):ti,ab,kw | 93,727 |
| #11 | #5 OR #6 OR #7 OR #8 OR #9 OR #10 | 93,781 |
| #13 | #4 AND #11 | 980 |

**5. CINAHL with Full Text（EBSCOhost）**

Database: CINAHL with Full Text

Platform: EBSCOhost (EBSCO)

Date last searched: April 7, 2026

Records retrieved: 76

| Search | Query | Items found |
| --- | --- | --- |
| S1 | TI ( multimorbid* OR "multiple morbidity" OR "multiple long-term condition*" OR "multiple chronic condition*" OR "multiple chronic disease*" OR "multiple chronic illness*" OR ((coexist* OR co-occur* OR cooccur* OR comorbid*) N3 ("chronic condition*" OR "chronic disease*" OR "chronic illness*")) ) | 3787 |
| S2 | AB ( multimorbid* OR "multiple morbidity" OR "multiple long-term condition*" OR "multiple chronic condition*" OR "multiple chronic disease*" OR "multiple chronic illness*" OR ((coexist* OR co-occur* OR cooccur* OR comorbid*) N3 ("chronic condition*" OR "chronic disease*" OR "chronic illness*")) ) | 5906 |
| S3 | MH "Multimorbidity+" OR MH "Multiple Chronic Conditions" | 2708 |
| S4 | S1 OR S2 OR S3 | 8459 |
| S5 | TI ( "digital health" OR ehealth OR "e-health" OR telemed* OR telehealth OR telecare OR telemonitor* OR "remote patient monitoring" OR "remote consultation" OR teleconsult* OR videoconsult* OR "mobile health" OR mhealth OR "m-health" OR "mobile app*" OR smartphone* OR "text messag*" OR sms OR "web-based" OR "internet-based" OR wearable* OR telerehabilitation OR email* OR "patient portal*" OR "electronic health record*" OR "electronic medical record*" OR "personal health record*" OR "clinical decision support" OR "decision support system*" OR "health information technolog*" OR "virtual care" ) | 61,427 |
| S6 | AB ( "digital health" OR ehealth OR "e-health" OR telemed* OR telehealth OR telecare OR telemonitor* OR "remote patient monitoring" OR "remote consultation" OR teleconsult* OR videoconsult* OR "mobile health" OR mhealth OR "m-health" OR "mobile app*" OR smartphone* OR "text messag*" OR sms OR "web-based" OR "internet-based" OR wearable* OR telerehabilitation OR email* OR "patient portal*" OR "electronic health record*" OR "electronic medical record*" OR "personal health record*" OR "clinical decision support" OR "decision support system*" OR "health information technolog*" OR "virtual care" ) | 96,396 |
| S7 | MH "Digital Health" OR MH "Telemedicine+" OR MH "Remote Patient Monitoring" OR MH "Remote Consultation" OR MH "Mobile Applications+" OR MH "Telerehabilitation" | 52,028 |
| S8 | S5 OR S6 OR S7 | 157,293 |
| S9 | TI ( randomized OR randomised OR randomly OR placebo OR "controlled trial*" OR "clinical trial*" OR "controlled clinical trial*" OR "random* allocat*" OR "cluster random*" OR "cluster-random*" OR "group random*" OR "group-random*" OR "stepped wedge" OR "stepped-wedge" ) | 202,273 |
| S10 | AB ( randomized OR randomised OR randomly OR placebo OR "controlled trial*" OR "clinical trial*" OR "controlled clinical trial*" OR "random* allocat*" OR "cluster random*" OR "cluster-random*" OR "group random*" OR "group-random*" OR "stepped wedge" OR "stepped-wedge" ) | 445,925 |
| S11 | S9 OR S10 | 527,964 |
| S12 | S4 AND S8 AND S11 | 76 |

**6. Scopus**

Database: Scopus

Platform: Elsevier

Date last searched: April 7, 2026

Records retrieved: 387

| Search | Query | Items found |
| --- | --- | --- |
| #1 | TITLE-ABS-KEY(multimorbid* OR "multiple morbidity" OR "multiple long-term condition*" OR "multiple chronic condition*" OR "multiple chronic disease*" OR "multiple chronic illness*" OR "coexisting chronic condition*" OR "co-existing chronic condition*" OR "coexisting chronic disease*" OR "co-existing chronic disease*" OR "coexisting chronic illness*" OR "co-existing chronic illness*" OR "co-occurring chronic condition*" OR "co occurring chronic condition*" OR "co-occurring chronic disease*" OR "co occurring chronic disease*" OR "co-occurring chronic illness*" OR "co occurring chronic illness*" OR "comorbid chronic condition*" OR "comorbid chronic disease*" OR "comorbid chronic illness*") | 23,546 |
| #2 | TITLE-ABS-KEY ( "digital health" OR ehealth OR "e-health" OR telemed* OR telehealth OR telecare OR telemonitor* OR "remote patient monitoring" OR "remote consultation" OR teleconsult* OR videoconsult* OR "mobile health" OR mhealth OR "m-health" OR "mobile app*" OR smartphone* OR "text messag*" OR sms OR "web-based" OR "internet-based" OR wearable* OR telerehabilitation OR email* OR "patient portal*" OR "electronic health record*" OR "electronic medical record*" OR "personal health record*" OR "clinical decision support" OR "decision support system*" OR "health information technolog*" OR "virtual care" ) | 1,069,018 |
| #3 | TITLE-ABS-KEY ( randomized OR randomised OR randomly OR placebo OR "controlled trial*" OR "clinical trial*" OR "controlled clinical trial*" OR "random* allocat*" OR "cluster random*" OR "cluster-random*" OR "group random*" OR "group-random*" OR "stepped wedge" OR "stepped-wedge" ) | 3,664,013 |
| #4 | #1 AND #2 AND #3 | 387 |

**7. Grey Literature Websites (Searched but no records retrieved)**

| Source | URL | Search method | Date searched | Records |
| --- | --- | --- | --- | --- |
| U.S. Food and Drug Administration | www.fda.gov | Browsed using terms "multimorbidity" and "digital health"; first 50 results screened | April 7, 2026 | 0 |
| GreyNet International | greynet.org | Browsed using terms "multimorbidity" and "digital health"; first 50 results screened | April 7, 2026 | 0 |
| OPENGREY | opengrey.eu | Searched using terms "multimorbidity" and "digital health" | April 7, 2026 | 0 |
| NTIS | www.ntis.gov | Searched using terms "multimorbidity" and "digital health" | April 7, 2026 | 0 |
| APA PsycExtra | www.apa.org/psycextra/ | Searched using terms "multimorbidity" and "digital health" | April 7, 2026 | 0 |

**8. Trial Registries**

| Registry | URL | Search method | Date searched | Records |
| --- | --- | --- | --- | --- |
| ClinicalTrials.gov | clinicaltrials.gov | Searched using terms: "multimorbidity" AND "digital health" | March 15, 2026 | 12 |
| WHO ICTRP | trialsearch.who.int | Searched using terms: "multimorbidity" AND "digital health" | March 15, 2026 | 7 |
| EU Clinical Trials Register | euclinicaltrials.eu | Searched using terms: "multimorbidity" AND "digital health" | March 15, 2026 | 4 |

**Note:** A supplementary search of trial registries was conducted during revision to identify ongoing or unpublished studies for contextual interpretation. These records were not part of the main study-selection pathway for the systematic review and meta-analysis and were therefore not included in the main PRISMA flow diagram or quantitative synthesis.

**9. Citation Searching**

| Method | Details |
| --- | --- |
| Reference list screening | Reference lists of all included studies and relevant systematic reviews were manually screened. |
| Forward citation tracking | Performed using Scopus and Web of Science for all included studies. |
| Date performed | April 7, 2026 |
| Records retrieved | 11 |
