# Supplementary material for: Effectiveness and Implementation of Digital Health Interventions on Physiological, Psychological, and Functional Outcomes in Adults With Multimorbidity: Systematic Review and Meta-Analysis of Randomized Controlled Trials
Source: J Med Internet Res. 2026 Jul 28;28:e90458. doi: 10.2196/90458 (PMC13412019; doi:10.2196/90458)
Supplement: Multimedia Appendix 4 [file jmir-v28-e90458-s004.docx]

**Table S1. Characteristics of included studies**

| **Author, year** | **Country / setting** | **Aim / research question** | **Study design** | **Participants** | **Intervention** | **Orientation** | **Comparator** | **Data collection (follow-up duration)** | **Main findings** |
| --- | --- | --- | --- | --- | --- | --- | --- | --- | --- |
| González-Ortega et al (2017) [65] | Spain  (High income)/ Urban primary care centers in Barcelona | To evaluate whether adding telephone coaching by a family physician to usual care reduces emergency visits/hospital admissions and improves health status, quality of life, and caregiver burden in complex chronic patients (CCPs). | RCT | N =161; Mean age = 80.5 (SD = 10); 52.8% female; Multimorbidity profile: high comorbidity (Charlson index mean 3.9); | **Telephone coaching** by external family physician (biweekly calls over 6 months, ~5–8 min each), based on patient-tailored unscripted counseling and EHR review. | Clinician-Facing | Usual care by assigned primary care providers (physicians and nurses) without additional coaching. | Baseline and 6-month follow-up; face-to-face interviews; outcomes validated via EHR and hospital records; instruments: SF-12, Barthel, Pfeiffer, Norton, Charlson, Zarit, Gijón scales. | - No statistically significant reduction in total urgent visits (mean difference 0.18, 95% CI −0.48 to 0.84).  - Physical component of SF-12 improved in the intervention group (mean difference vs. control: +4.71 points, 95% CI 0.41 to 9.03; calculated as control minus intervention).  - No significant differences were observed in mental health, functional status, or caregiver burden. |
| Chan et al (2022) [36] | Multinational /13 hospital-based diabetes centers in 8 countries/regions in Asia: China (Upper middle income), Hong Kong, China (High income), Malaysia (Upper middle income), Philippines (Lower middle income), South Korea (High income), Taiwan, China (High income), Thailand (Upper middle income), Vietnam (Lower middle income) | To evaluate the effect of the Joint Asia Diabetes Evaluation (JADE) web portal, nurse  reminders, and team-based care on multiple risk factors in patients with DKD. | Multinational RCT (3-arm) | N = 2,393; Mean age = 67.7 (SD = 9.8); 52.9% female; Multimorbidity profile: all participants had type 2 diabetes with DKD (89.6% with eGFR <65, 36.2% with macroalbuminuria, 30.9% with cardiovascular disease). | **Web-based platform:** (1) Empowered care: JADE web portal–based personalized reports + nurse telephone follow-up every 3 months; (2) Team-based empowered care: As above + in-person clinic visits with physician-nurse team every 3 months. | Clinician-Facing | Usual care: standard medical management at study sites. | Structured assessments via JADE portal at baseline and 12 months; data from physical exams, labs, and patient self-report questionnaires. | - The team-based empowered care group showed the greatest improvement in attainment of ≥3 treatment targets (HbA1c, BP, LDL-C, TG, RAAS inhibitor use), with 9.1% within-group increase. - Compared to usual care, team-based care increased the likelihood of attaining targets (RR 1.20, 95% CI 1.03–1.40). - Significant reductions in HbA1c (−0.39%) and LDL-C (−0.14 mmol/L) in the team-based group. - Improved patient self-care behaviors in intervention groups.  - Among all participants, those who attained multiple treatment targets had a lower incidence of CV/kidney/cancer events compared to those who did not (8.4% vs 14.5%, *P*=.004). |
| Baumeister et al (2021) [48] | Germany (High income)/ 82 orthopedic rehabilitation clinics | To evaluate the effectiveness of a guided internet- and mobile-based intervention (IMI) for patients with chronic back pain (CBP) and depression in routine care, focusing on depression severity, remission, pain, QoL, and work capacity. | RCT (multicenter) | N = 210; Mean age ~50 years; 60% female (125/209); Multimorbidity profile: chronic back pain with mild-to-moderate depressive disorder (DSM-5 diagnosed). | **Guided Internet- and Mobile-Based CBT** (eSano BackCare-D): 6 core + 3 optional modules; approx. 1 session/week over 9 weeks; access via secured platform Minddistrict; optional SMS reminders and eCoach feedback. | Patient-Facing | Treatment-As-Usual (TAU): access to routine care including psychotherapist/psychiatrist visits and antidepressants. | Baseline, 9 weeks (T1), 6 months (T2); via online self-report and telephone interviews; instruments: HAM-D, PHQ-9, QIDS, SCID, AQoL-6D, ODI, PSEQ. | - No significant difference in clinician-rated depression severity at 9 weeks (HAM-D).  - Significant improvement in self-reported depression (PHQ-9), pain-related outcomes, and QoL at T1 and T2.  - Remission rates significantly higher in IG at T2 (OR 1.97, *P*=.035). |
| Liang et al (2021) [57] | Taiwan, China (High income)/ 400-bed regional hospital and home setting | To evaluate the effectiveness of a nurse-led, integrated tele-homecare program for older patients with multiple chronic illnesses (MCIs) and a high risk of readmission. | RCT | N = 200; Mean age = 80.67 (SD = 7.29); 58% female; Multimorbidity profile: mean number of chronic conditions = 2.72; high readmission risk defined by LACE index ≥7. | **Nurse-led integrated tele-homecare** using: one-touch smartphone, BP monitor, medication dispenser, glucometer, emergency call button; 24-hr call center; twice-daily monitoring; home visits at discharge, 3 months, and 6 months; plus 121 individualized home visits. | Clinician-Facing | Usual care: discharge planning + routine home care visits at 3 and 6 months (assessment, education, vitals, consultation); no telemonitoring. | Baseline (T0), 3 months (T3), 6 months (T6); instruments: C-MABS, ADLs, EQ-5D-5L, EQ-VAS; mortality, readmission, ED visits collected via hospital records. | - Mortality: Significantly reduced in intervention group (8% vs 19%; OR 0.371, *P*=.027).  - ED Visits: Significantly fewer in intervention group (12% vs 26%; OR 0.388, *P*=.013).  - Readmission: No significant difference (44% vs 41%; *P*=.668).  - Quality of Life (EQ-5D-5L): Improved at 6 months in intervention group (β=2.391, *P*=.003).  - Medication Adherence, ADLs, Health Status: No significant group × time interaction effect. |
| Wakefield et al (2011) [41] | USA (High income)/ Iowa City Veterans Affairs Medical Center (ICVAMC) | To evaluate the efficacy of a nurse-managed home telehealth intervention in improving glycemic control (HbA1c), systolic blood pressure (SBP), and adherence among veterans with comorbid diabetes and hypertension. | RCT (3-arm) | N = 302; Mean age: 68 years (range 40–89), 2% female, 96% White; Multimorbidity profile: all had comorbid type 2 diabetes and hypertension; received VA primary care. | **Home telehealth** (Viterion device), nurse-managed care over 6 months. (1)High-intensity: daily BP, BG input + full branching educational content (diet, exercise, meds, foot care, etc.). (2)Low-intensity: daily BP + 1 daily yes/no or multiple-choice question. | Clinician-Facing | Usual care: access to nurse care manager; standard VA primary care. | Baseline, 6 months (post-intervention), 12 months (maintenance); A1c from lab, SBP via automatic BP machine; adherence via validated self-report scales; depressive symptoms (GDS). | - A1c: Significant reduction at 6 months in both intervention groups vs control (*P*<.05), but effect not sustained at 12 months.  - SBP: Significant reduction in high-intensity group vs others at 6 and 12 months (*P*<.05).  - Adherence: Improved in all groups over time, no between-group difference.  - No serious adverse events reported. |
| Tchalla et al (2025) [58] | France (High income)/ Home-based care in rural and urban settings | To evaluate the feasibility and effectiveness of a 12-month home telemonitoring program (eCOBAHLT) in preventing rehospitalizations among older adults (≥65 years) with multimorbidity following hospital discharge. | RCT (multicenter) | N = 534; Mean age = 80.3 (SD = 8.1); 52.4% female; Multimorbidity profile: all had ≥2 chronic conditions; Common conditions included CHF (34.1%), stroke (21.5%), diabetes (14.4%). | **Home-based remote monitoring** using e-GEROPASS software and 7 external biometric sensors (24/7 data transmission); monthly research staff calls; real-time geriatric review and GP feedback. | Clinician-Facing | Usual care by general practitioner; no DHT components. | Baseline and 12 months; monthly telephone interviews; biometric data collected continuously (intervention); hospitalizations tracked prospectively. | - Significantly fewer unplanned hospitalizations in intervention group: 40.4% vs 48.7% (*P*=.043); adjusted RR = 0.72 (95% CI 0.51 to 0.94).  - Higher rate of ED visits without admission in intervention group (6.0% vs 2.2%, *P*=.027).  - No adverse effects reported; program was feasible (92.9% completion). |
| Yao et al (2021) [31] | China (Upper middle income)/ 40 centers across tertiary hospitals | To evaluate whether an integrated care approach supported by mHealth technology (based on the ABC pathway) can reduce atrial fibrillation–related adverse events in patients with multimorbidity. | Cluster RCT | N = 1,890; Mean age = 72.0 (intervention), 72.8 (control); 33.4% female (intervention), 41.9% female (control); Multimorbidity profile: hypertension (81.1%), CAD (62.8%), diabetes (39.7%), pulmonary disease, PAD, prior stroke, HF, etc. | **Mobile health (mHealth) technology implementing the ABC** (Atrial Fibrillation Better Care) pathway: - A: stroke prevention; - B: symptom management; - C: comorbidity management; Participants used a smartphone-based app (mAFA App); follow-up mean: 419 days. | Clinician-Facing | Usual care: standard local practice, not protocolized. | Baseline to end of follow-up; mean follow-up: 419 (257) days in intervention vs 457 (154) days in control group; outcomes tracked via clinical records, app logs, and events adjudication. | - Composite outcome (stroke/thromboembolism, all-cause death, rehospitalization) significantly reduced in intervention group (HR=0.37, 95% CI 0.26 to 0.53, *P*<.001).  - Rehospitalization significantly reduced (HR=0.42, 95% CI 0.27 to 0.64).  -Stroke/ thromboembolism lower in intervention (0.5% vs 2.9%).  - C outcome (ACS, HF, uncontrolled BP): 3.2% vs 13.7%, HR=0.29.  - No significant difference in bleeding or symptom control.  - Subgroup effects consistent by age, sex, prior stroke. |
| Monreal-Bartolomé et al (2025) [52] | Spain (High income)/ Primary care centers in Andalusia, Aragon, and the Balearic Islands | To assess the effectiveness of a blended low-intensity psychological intervention (internet-delivered + face-to-face) combined with improved treatment as usual (iTAU), versus iTAU alone, for patients with multimorbidity (depression and type 2 diabetes or low back pain) in primary care settings. | RCT | N=183; Mean age = 51.4 (SD 11.3); 72.1% female; Multimorbidity profile: major depressive disorder (mild to moderate) + either type 2 diabetes or chronic low back pain. | **Blended low-intensity psychological program**: Blended low‑intensity psychological program: 2 face‑to‑face sessions combined with 6 web‑based modules delivered via a secured online platform (Minddistrict); each module (~60 minutes) included psychoeducation, behavioral activation, positive psychology, and mindfulness exercises; participants received motivational support and feedback from an e‑coach; intervention duration 8–12 weeks. | Patient-Facing | Improved treatment as usual (iTAU) based on national guidelines for depression; includes GP training. | Baseline, post-intervention, and 3-month follow-up (primary endpoint); instruments included PHQ-9, HbA1c, RMDQ, FPS-R, SF-12, PANAS, OFS. | - Composite outcome score improved significantly in the intervention group versus control (regression coefficient B=-0.34, 95% CI -0.64 to -0.04; Hedges g=0.39). - Greater reduction in depression (PHQ-9), negative affect, and improved perceived health, positive affect, and openness to the future. - No significant effect on HbA1c, pain intensity, or disability at 3 months. - Positive and negative affect mediated the intervention effect on composite outcome. |
| Rollman et al (2021) [40] | USA (High income)/ 8 university-based and community hospitals in southwestern Pennsylvania | To determine whether a blended collaborative care program for treating both heart failure (HF) and depression can improve clinical outcomes more than collaborative care for HF only and physicians’ usual care (UC). | RCT (3-arm) | N = 756; Mean age = 64.0 (SD 13.0); 44% female; Multimorbidity profile: all had heart failure with reduced ejection fraction (<45%), 20–40% had comorbid depression; common comorbidities include hypertension, diabetes, hyperlipidemia. | **Telephone-delivered blended collaborative care** (HF + depression): Nurse-led care with weekly to monthly follow-ups over 12 months; care included symptom monitoring, psychoeducation, PHQ-9 tracking, medication support, and physician review. | Clinician-Facing | (1) Enhanced Usual Care (eUC): Collaborative care for HF only (without mental health component); (2) Usual Care (UC): Standard physician-led care with no structured intervention. | Assessments at baseline, 3, 6, and 12 months via telephone; outcome measures: MCS-12, PHQ-9, PROMIS-D, HRS-D, KCCQ-12, PCS-12, rehospitalization, and mortality; Follow-up duration: 12 months. | - Blended care improved mental health-related quality of life (MCS-12) compared with usual care (+4.47 points, 95% CI 1.65 to 7.28; P=.002), but not compared with enhanced usual care (+1.12 points, 95% CI −1.15 to 3.40; P=.33).  - Mood improved on PROMIS-D in the blended care group compared with both usual care (ES=0.47, 95% CI 0.28 to 0.67) and enhanced usual care (ES=0.24, 95% CI 0.07 to 0.41).  - HRS-D mood symptoms showed a differential improvement only among women receiving blended care versus usual care.  - No significant differences were observed in physical function, physical HRQoL, HF pharmacotherapy use, rehospitalizations, or mortality. - Telephone-delivered blended collaborative care improved selected mental health outcomes but did not improve physical or event-based outcomes. |
| Gustafson Sr et al (2024) [62] | USA (High income)/ Primary care clinics at University of Wisconsin–Madison | To evaluate the effectiveness of an enhanced version of the ElderTree (ET) eHealth intervention in improving mental and physical quality of life, psychological well-being, and loneliness among older adults with multiple chronic conditions (MCCs). | RCT | N = 344; Mean age ≈ 74.7; 60.8% female; Multimorbidity profile: mean number of chronic conditions = 5.28; Inclusion: ≥65 years with ≥3 of 11 predefined chronic conditions (e.g., hypertension, diabetes, COPD, etc.) based on EHR. | **ElderTree (ET):** Web-based information and peer support platform tailored to older adults with MCCs; components included weekly self-monitoring surveys (10 domains), personalized feedback, clinician summary reports, community chat groups, well-being content, and lifestyle articles; access via laptop + internet for 12 months. | Patient-Facing | Attention control: laptop + internet + 4 static health websites (e.g., Cleveland Clinic, Mayo Clinic); no interactive features or peer/social components. | Baseline, 6-month, and 12-month surveys; instruments: PROMIS, UCLA Loneliness, Flourishing Scale, etc. ET usage logs continuously collected. | - Significant improvement in mental quality of life (PROMIS T-score) was observed in the intervention group compared with control (Δ=+1.5 points; P=.04); the effect was stronger in women (Δ=+2.6; P=.002) and was mediated by improved relatedness. - No significant effects on physical QOL, loneliness, or well-being overall. - ET use significantly predicted improvements in mental QOL (≥167 days/6 months for clinically meaningful change). - No differences in symptom distress, medication adherence, or crisis care use. - Intervention was feasible and sustained (>75% still active at 12 months). |
| Bernocchi et al (2018) [56] | Italy (High income)/3 hospitals, home-based setting post-rehabilitation | To evaluate the feasibility and effectiveness of a 4-month integrated home-based telerehabilitation program (Telereab-HBP) for older patients with coexisting chronic obstructive pulmonary disease (COPD) and chronic heart failure (CHF), focusing on exercise tolerance and other health outcomes. | RCT (multicenter) | N = 112; Mean age ~70 years; 18% female; Multimorbidity profile: all had both COPD (GOLD B-D) and CHF (NYHA II–IV). | **Telerehabilitation (Telereab-HBP):** Weekly remote nurse and physiotherapist calls; Remote monitoring (pulse oximeter, ECG); Home-based exercise (3–7 times/week depending on level); 4 months intervention + 2-month post-intervention follow-up. | Clinician-Facing | Usual care: standard medical therapy, education, and self-initiated physical activity recommendations without remote monitoring or scheduled follow-up. | Baseline (T0), 4 months (T1, end of intervention), and 6 months (T2, 2 months post-intervention); Data collected via in-person assessments, 6MWT, questionnaires (MLHFQ, CAT), clinical data. | - 6MWT increased by 60m (95% CI 22.2 to 97.8) in IG vs −15m in CG (P=.004).  - Time to hospitalisation/death was longer in IG (median 113.4 vs 104.7 days, P=.048).  - Significant improvements in IG vs CG for: Dyspnoea (MRC, P=.05); Physical activity (PASE, P=.0015); Disability (Barthel, P=.0006); QoL (MLHFQ, P=.0007; CAT, P<.001).  - Benefits sustained at 6 months. |
| Mihevc et al (2025) [55] | Slovenia (High income)/ Primary healthcare centers in urban (Ljubljana) and rural regions (Trebnje, Slovenj Gradec) | To evaluate the impact of a 12-month mHealth home telemonitoring intervention on clinical outcomes in older individuals with arterial hypertension (AH) and type 2 diabetes (T2D), compared to standard care. | RCT (multicenter) | N = 117; Mean age = 71.3 years (SD 4.7); 39.3% female; Multimorbidity profile: all participants had both AH and T2D (≥1 year); 20.5% had CKD; 47.9% had obesity. | **mHealth-supported home telemonitoring via smartphone app:** BP monitor (2x/week, morning & evening readings), BG monitor (monthly, fasting + postprandial); data transmitted via Bluetooth to cloud-based telemedicine platform; alerts triggered more intensive profiles or teleconsultation with GPs. | Clinician-Facing | Standard integrated care delivered by GPs and nurses per national chronic disease protocols, including annual checkups, health promotion referrals, and education. | Baseline, 6 months, 12 months; Self-monitoring + clinical assessments (blood tests, questionnaires, validated instruments); Outcomes measured: SBP, DBP, HbA1c, fasting BG, lipid profile, BMI, GFR, quality of life (Appraisal of Diabetes Scale), and behavioral risk factors. | - Significant reductions in SBP (−9.7 mmHg vs −2.8 mmHg; between-group Δ = −6.9 mmHg, *P*=.001).  - Significant HbA1c reduction in intervention group (−0.5%, *P*<.001); between-group Δ = −0.5%, *P*=.002.  - No significant effects on other outcomes (DBP, lipid profile, BMI, GFR, quality of life, lifestyle) after adjustment for multiple comparisons.  - Intervention was feasible, with 91.4% completing follow-up. |
| Yu et al (2020) [37] | Canada (High income)/ 10 Family Health Teams (primary care clinics) in Southwestern Ontario | To assess the impact of MyDiabetesPlan, a web-based shared decision-making and goal-setting tool, on decisional conflict, diabetes distress, health-related quality of life, and patient assessment of chronic illness care in adults with diabetes and multimorbidity. | Cluster RCT(2-step) | N = 213; Majority ≥55 years; 54.9% female in intervention group; 41.4% female in control group; Multimorbidity profile: adults (≥18 years) with diabetes (type 1 or type 2) and ≥2 comorbidities (e.g., heart disease, stroke, hypertension, arthritis, cancer, etc.) | **Web-based patient decision aid:** MyDiabetesPlan (includes provider- and patient-directed components); clinicians trained at baseline; patients used system before clinical appointments; used ≥2 times during follow-up. | Patient-Facing | Usual care: static Diabetes Canada resources (clinician-directed initially; then patient education pamphlet + web resources after 6 months). | Baseline, 6 months, 12 months; via online or mail survey; instruments: DCS, DDS, SF-12, PACIC. | - Modest reduction in decisional conflict at 12 months (−3.5 points; 95% CI −7.4 to 0.4).  - Improved patient assessment of chronic illness care (PACIC +0.7 points; 95% CI 0.4 to 1.0; *P*<.001).  - No significant changes in diabetes distress or quality of life.  - Greater impact in patients >65 years or higher income.  - No reported adverse events. |
| Jungo et al (2023) [38] | Switzerland (High income)/ Primary care (43 GP clusters) | To evaluate whether a structured medication review centred around an electronic clinical decision support system (eCDSS) improves medication appropriateness and reduces prescribing omissions in older adults with multimorbidity and polypharmacy compared with usual care. | Cluster RCT | N = 323; Median age = 77 (IQR 73–83); 45% female; Multimorbidity profile: all aged ≥65 years with ≥3 chronic conditions and ≥5 long-term medications. | **Electronic Clinical Decision Support System (eCDSS)** – STRIPA tool based on STOPP/START criteria; one-time medication review with shared decision-making. | Clinician-Facing | Usual care: standard GP-led medication discussion without eCDSS. | Baseline, 6 months, 12 months; data from EHR (FIRE project), phone interviews, GP reports. | - No statistically significant improvement in medication appropriateness (MAI: OR 1.05; 95% CI 0.59–1.87) or prescribing omissions (AOU: OR 0.90; 95% CI 0.41–1.96) at 12 months.  - 58.5% of patients had ≥1 recommendation implemented.  - Adverse drug events were fewer in the intervention group (6 vs 9), but this difference was not statistically significant (p = 0.42).  - The intervention was safe and well accepted, but results were inconclusive for the primary and most secondary outcomes. |
| Blum et al (2021) [39] | Switzerland (High income), Netherlands (High income), Belgium (High income), Republic of Ireland (High income)/ inpatient wards in university-based hospitals | To examine the effect of optimising drug treatment on drug related hospital admissions in older adults with multimorbidity and polypharmacy admitted to hospital. | Cluster RCT | N= 2,008; Median age = 79 ( ≥70 years); 44.7% female; Multimorbidity profile: all with ≥3 chronic conditions and ≥5 long-term medications. | **Web-based STRIP Assistant (STRIPA):** One-time structured drug review during hospital admission; Conducted by a trained doctor–pharmacist team using STOPP/START criteria, SHiM questionnaire, lab values, and patient preferences; Recommendation report shared with hospital doctors and general practitioners. | Clinician-Facing | Usual care: no structured review; unstructured medication review possible. | Data collected at 2, 6, and 12 months; telephone interviews with participants/proxies; adjudicated hospital admissions; instruments: EQ-5D, Barthel Index, MMAS-8, etc. | - No significant reduction in first drug-related hospital admission (HR 0.95; 95% CI 0.77–1.17).  - 86.1% received STOPP/START recommendations; 62.2% had ≥1 implemented.  - No significant differences in mortality, falls, adverse events, or medication adherence.  - Slight improvement in quality of life at 12 months (adjusted mean difference = 2.29; 95% CI 0.31–4.26).  - Intervention was safe and well accepted. |
| Clarke et al (2019) [63] | Australia (High income)/ Community-based, general practice settings, and online recruitment | To evaluate the effectiveness of a self-guided web-based cognitive behavioral therapy (CBT) program (myCompass) in improving social and occupational functioning in adults with type 2 diabetes (T2DM) and mild-to-moderate depressive symptoms; secondary aims included impact on depression, anxiety, diabetes distress, and diabetes self-care behaviors. | RCT | N = 780; Mean age ~58 years (SD 10.4); ~68.8% female; Multimorbidity profile: all had T2DM + mild-to-moderate depressive symptoms (PHQ-9), ~40.9% had previous depression diagnosis. | **myCompass:** a fully automated, unguided, web-based cognitive behavioral therapy (CBT) program accessed via mobile or computer for 8 weeks + 4-week taper (symptom monitoring only); personalized CBT module recommendations and symptom tracking; frequency: mean 6 logins, ~0.29 modules completed. | Patient-Facing | Active placebo: Healthy Lifestyles – Web-based health literacy program (e.g., skin care, hygiene), 12 modules; same delivery format and SMS/email reminders, but without therapeutic content. | Baseline and 3 months postintervention (primary endpoint); instruments: WSAS, PHQ-9, DDS, GAD-7, SMP-T2D behavior items (e.g., medication adherence, blood glucose monitoring) | - No significant between-group differences on primary outcome (WSAS) or most secondary outcomes.  - Both groups showed modest improvements over time in depression, anxiety, diabetes distress, and healthy eating.  - Control group showed small but significantly better outcomes for blood glucose monitoring and medication adherence.  - No moderation by age, glycemic control, or depression severity.  - Low program engagement in the intervention group (mean 0.29 modules completed). |
| Lear et al (2021) [64] | Canada (High income)/ 71 primary care clinics in small urban and rural areas of British Columbia | To evaluate whether an internet-based chronic disease self-management (CDM) and symptom monitoring program integrated into primary care can reduce hospitalizations among patients with multiple chronic diseases over a 2-year period. | RCT | N = 229; Mean age = 70.5 (SD 9.1); 39.4% female; Multimorbidity profile: ≥2 of the following — diabetes, heart failure, ischemic heart disease, chronic kidney disease, or COPD. | **Internet-based self-management and symptom monitoring program integrated with primary care:** weekly (or daily initially) online symptom/biometric reporting (weight, BP, blood glucose), email prompts, alerts to nurse, nurse-led follow-up (via phone), shared action plans, support from dietitian and exercise specialist, lifestyle questionnaires every 8 weeks; median logins per week: 2.5; 84.5% logged in ≥1x/week. | Clinician-Facing | Usual care: general educational resources and no structured digital intervention. | Baseline, 12 months, and 24 months via telephone interview and electronic health records; instruments included SF-36, heiQ, MOS-SSS. | - No statistically significant reduction in all-cause hospitalizations (RR 0.68, 95% CI 0.43–1.10; *P*=.12).  - Fewer participants in intervention group hospitalized (27.6% vs 40.7%; OR 0.55, 95% CI 0.31–0.96; *P*=.03).  - Composite outcome (hospitalization or death) lower in intervention group (31.9% vs 45.1%; OR 0.57, 95% CI 0.33–0.98; *P*=.04).  - Time to first hospitalization was significantly delayed in the intervention group (HR 0.62, 95% CI 0.39–0.97; *P*=.04).  - Compared to control, the intervention group showed improved self-management in 4 of 8 domains (as measured by the heiQ) and improved social support in 2 of 5 domains of the MOS‑SSS.  - High adherence: 96.6% completed 2-year intervention. |
| Prabhakaran et al (2019) [32] | India (Lower middle income)/ 40 Community Health Centers (CHCs) across Haryana and Karnataka | To evaluate the effectiveness of the mWellcare mHealth-based electronic decision support (EDS) system for integrated management of five chronic conditions (hypertension, diabetes mellitus, depression, tobacco and alcohol use) in primary care settings in India, using hypertension and diabetes as entry points. | Cluster RCT (multicenter) | N = 3,698; Mean age = 55.1 (SD 11.0); 44.8% female; Multimorbidity profile: All had hypertension and/or diabetes at baseline; study also addressed depression, tobacco, and alcohol use; 50% had hypertension only; ~35% had diabetes (including overlap); ~15% had both conditions. | **mWellcare Android-based mHealth system:** Electronic health record (EHR) storage; clinical decision support system (CDSS) generating personalized treatment recommendations; SMS reminders for follow-up and adherence; used during every clinical visit; | Clinician-Facing | **Enhanced usual care (EUC):** Same clinical training, nurse staffing, and pamphlets as intervention group; No EDS system; tablets used only for baseline data; Treatment decisions made by physician based on judgment and simplified charts. | 12 months follow-up; measures: SBP, HbA1c, FBG, total cholesterol, BMI, Framingham CVD risk, tobacco & alcohol use, depression (PHQ-9), medication adherence; Instruments: Omron BP monitor, glucometer (capillary blood), HbA1c labs, PHQ-9, AUDIT. | - No statistically significant difference in SBP (Δ = −1.0 mmHg; 95% CI: −4.6 to 2.7) or HbA1c (Δ = +0.11%; 95% CI: −0.24 to 0.45) between groups.  - Both groups improved over time: SBP −13.7 (mWellcare) vs −12.7 mmHg (EUC); HbA1c −0.48% vs −0.58%.  - No between-group differences in any secondary outcomes.  - Self-reported medication adherence (secondary outcome) higher in mWellcare group (e.g., antihypertensive adherence 81.1% vs 57.9%).  - Intervention feasible, but incremental benefit over EUC not demonstrated |
| Yoo et al (2009) [53] | South Korea (High income)/ Korea University Hospital (tertiary) and Guro-Gu Public Health Centre (community) | To evaluate the effectiveness and feasibility of a Ubiquitous Chronic Disease Care (UCDC) system using both cellular phones and the internet in improving multiple metabolic parameters in overweight patients with coexisting type 2 diabetes and hypertension. | RCT | N = 123 at baseline; 111 completed the study; Mean age ~58 years (30–70 years); ~42% female; Multimorbidity profile: Overweight (BMI ≥23.0 kg/m²) patients with both Type 2 diabetes and hypertension (diagnosed ≥1 year). | **UCDC system integrating:** Mobile phone-based self-monitoring of blood glucose (2x/day), blood pressure (2x/day), and weight (1x/day); Automated feedback via SMS based on ADA and Korean Diabetes guidelines (3x/day); Daily exercise logging via SMS response; Internet-based physician interface for remote monitoring and individualized feedback; Daily automated and interactive engagement over 12 weeks. | Patient-Facing | Usual care: routine outpatient visits and standard physician management; no digital intervention. | 12 weeks (3 months); assessments at baseline and endline; data from clinical exams, SMBG, HBPM/OBPM, lab tests, and arterial stiffness measurements. | - Significant improvements in the intervention group compared to control for: HbA1c (−0.5%, P<.001); Systolic and diastolic blood pressure (both office‑based and home‑based measurements); LDL cholesterol, total cholesterol, triglycerides; Adiponectin (increase) and baPWV (decrease, indicating improved arterial stiffness). - No significant changes in hsCRP or IL-6. - No medication changes; high adherence (compliance rate >85% for BG/BP measurements); - Intervention was feasible, safe, and well accepted. |
| Gellis et al (2014) [59] | USA (High income) / Hospital-affiliated home healthcare setting | To evaluate an integrated telehealth intervention (I-TEAM) to improve chronic illness (CHF, COPD) and comorbid depression in home healthcare settings. | RCT | N = 102; Mean age = 79 years; 65.7% female; Multimorbidity profile: all had CHF or COPD and comorbid depressive symptoms (positive depression screen). | **Integrated Telehealth (I-TEAM):**daily telemonitoring (weight, blood pressure, pulse, oxygen saturation) plus 8 weekly sessions of problem-solving treatment for depression delivered by a telehealth nurse over 3 months. | Clinician-Facing | Usual in-home care + psychoeducation (UC+P). | Baseline, 3 months, 6 months, and 12 months; instruments: HAM-D, PHQ-9, SF-12, SPSI-R, and health care utilization data. | - Depression scores (HAM-D and PHQ-9) were significantly lower in the I-TEAM group at 3 and 6 months. - I-TEAM significantly improved problem-solving skills and self-efficacy. - The intervention group had significantly fewer ED visits at 12 months (0.6 vs 1.4; P = .01). - No significant between-group difference was observed in hospital days at 12 months. |
| Bothelius et al (2024) [47] | Sweden (High income) / University hospital pain clinic | To assess the feasibility and efficacy of internet-based CBT for insomnia (ICBT-i) for patients with chronic benign pain and comorbid insomnia in a routine care setting. | RCT | N = 85; Mean age ~48 years; 71% female; Multimorbidity profile: chronic benign pain (IASP criteria) + insomnia (ISI >10). | **Internet-based CBT for Insomnia (ICBT-i):**8 text-based modules (sleep restriction, stimulus control, cognitive restructuring) over 8 weeks, adapted for chronic pain. Guided therapist support via messaging. | Patient-Facing | Internet-based Applied Relaxation (IAR): 8 text-based modules over 8 weeks, focusing on relaxation techniques. | Baseline, post-treatment (8 weeks), and 6-month follow-up; instruments: ISI, BIS, BPI-SF, MADRS-S, GAD-7, WSAS, BBQ, CORE-10, DBAS-10, SPAQ. | - Low treatment engagement (avg. 2.0/8 modules for ICBT-i, 2.4/8 for IAR). - Both groups significantly improved insomnia symptoms, but no significant between-group difference on primary outcome (ISI). - IAR was superior to ICBT-i in reducing pain interference and anxiety at post-treatment (p=.003 & p=.010). - Gains were stable at 6 months, with ICBT-i showing some delayed benefits. |
| Sanabria-Mazo et al (2023) [42] | Spain (High income) / Pain units at two hospitals (Parc Sanitari Sant Joan de Déu, Hospital del Mar) | To examine the efficacy of adding remote, synchronous, group videoconference-based ACT or BATD to TAU for chronic low back pain (CLBP) plus comorbid depressive symptoms. | RCT (3-arm, multicenter) | N = 234; Mean age ~54.5 years; 67.5% female; Multimorbidity profile: CLBP (>3 months, pain >4/10) + moderate-to-severe depressive symptoms (PHQ-9 ≥10). | **Group videoconference-based (Zoom) ACT or BATD:** 8 weekly 1.5-hour sessions + TAU. Both therapies delivered in groups of 7-13 participants. | Patient-Facing | Treatment-As-Usual (TAU): management by general practitioners (medication, exercise advice). | Baseline, post-treatment (2 months), and 12-month follow-up; instruments: BPI-IS, NRS, DASS-21, PCS, CPAQ-8, BADS-SF, PIPS. | - ACT significantly reduced pain interference vs TAU at post-treatment (d=0.64) and 12 months (d=0.73). BATD was superior to TAU at 12 months (d=0.66).  - ACT and BATD reduced pain catastrophizing vs TAU at post-treatment (d=0.45 & 0.59) and 12 months (both d=0.59).  - No significant between-group differences in depression or anxiety symptoms.  - Improvements in pain interference were related to changes in psychological flexibility. |
| Or et al (2020) [54] | Hong Kong, China (High income) / Outpatient clinics of two public hospitals | To test the effectiveness and safety of a prototype technological surrogate nursing (TSN) app for self-care in patients with coexisting type 2 diabetes and hypertension. | RCT | N = 299; Mean age ~63.8 years; 34% female; Multimorbidity profile: all had physician-confirmed type 2 diabetes and hypertension for ≥1 month. | **Technological Surrogate Nursing (TSN) app:** Tablet-based app with Bluetooth-connected monitors for self-monitoring of BG and BP, structured data display, educational resources, and programmable reminders. | Patient-Facing | Conventional self-management: supplied with same BG and BP monitors and logbooks, but no TSN app. | Baseline, 8, 12, 16, and 24 weeks; primary outcomes: HbA1c, SBP, DBP; secondary outcomes: medication adherence, disease knowledge, self-efficacy. | - HbA1c significantly improved in both groups from baseline, but no significant between-group difference.  - No significant between-group differences in SBP or DBP.  - Significant improvements in medication adherence, disease-specific activity adherence, and disease knowledge within the intervention group, but not significantly different from control.  - Study provides a method to infer patient self-safety from the data. |
| Gasslander et al (2022) [46] | Sweden (High income) / Specialist pain clinic at Uppsala University Hospital | To investigate if a guided, individually tailored, internet-delivered CBT (ICBT) program can improve mood and reduce disability in individuals with chronic pain and comorbid psychological distress. | RCT | N = 187; Mean age 45.9 years; 73% female; Multimorbidity profile: chronic pain (>3 months) + at least one form of psychological distress (e.g., depression, anxiety, PTSD, insomnia). | **Individually Tailored ICBT:** 6-13 modules selected from a bank of 20 (e.g., BA, sleep, worry, trauma, communication) over 8-10 weeks. Guided therapist feedback via secure messaging. | Patient-Facing | Waiting-list control (WLC), offered treatment after post-measurements. | Baseline, post-treatment (3 months), and 12-month follow-up; instruments: MADRS-S, MPI-S, HADS, CPAQ, CSQ-R, PCS, QOLI, ASI, PDI, PSEQ-2, TSK-11. | - Significant improvements in depression (MADRS, d=0.18) and pain interference (MPI, d=0.22) for ICBT vs WLC.  - Significant improvements in pain acceptance (CPAQ, d=0.30), catastrophizing (CSQ, d=-0.15), and quality of life (QOLI, d=0.02).  - All significant changes were stable at 12-month follow-up.  - Low-to-moderate treatment adherence (avg. 5.1 of 10.2 modules completed). |
| Hwang et al (2025) [51] | South Korea (High income) / Public health center & senior welfare center | To evaluate the effectiveness of a digital health coaching self-management program (DHCSMP-MCC) for older adults living alone with multiple chronic conditions. | RCT | N = 49; Mean age = 72.4 years; 76.2% female; Multimorbidity profile: ≥2 chronic conditions, mean 4.0 conditions; all participants lived alone. | **DHCSMP-MCC:** 8-week mHealth intervention via "HAHA 2022" app, combining educational videos, individual telephone health coaching (goal-setting, action plans), self-monitoring (meds, diet, sleep, steps via smart band), and reminders. | Patient-Facing | Usual lifestyle. | Baseline and 8 weeks; instruments: SC-CII, MMAS-8, EQ-5D-5L, EQ-VAS, DHTL-AQ, Self-Care Self-Efficacy scale, GAS. | - Significant group × time interactions were observed for health distress (P = .049) and depression (P = .025) in favor of the intervention group.  - Significant improvement was observed in digital health literacy, particularly in the “use of an app” subdomain (group × time P = .012).  - No significant between-group differences were observed in self-management behaviors, medication adherence, health-related quality of life, or self-efficacy.  - High GAS scores suggested good goal attainment in the intervention group. |
| Landucci et al (2025) [43] | USA (High income) / University of Wisconsin–Madison health system and community organizations | To test whether older adults with chronic pain and multiple chronic conditions use and benefit more from the ElderTree eHealth intervention when delivered on a smart display versus a touchscreen laptop, compared with usual care. | RCT (3-arm) | N = 268; Mean age = 69.8 years (SD = 7.34); 66.0% female; Multimorbidity profile: chronic pain plus ≥3 chronic conditions (mean number of chronic conditions = 6.54, SD = 2.46). | **ElderTree (ET):**Web-based information and peer support platform (discussion groups, private messages, wellness activities, health library, weekly surveys, pain course). Delivered via (1) smart display (Google Nest Hub Max, voice+touch) or (2) touchscreen laptop. | Patient-Facing | Usual care (no device or ElderTree). | Baseline, 4, and 8 months; primary outcomes: PROMIS-29 pain interference & psychosocial QoL. | - No significant differences were found between the laptop and smart display groups, or between the combined intervention groups and control, for pain interference or psychosocial quality of life.  - Gender moderated the effect of the combined intervention versus control on pain interference (P = .04); women in the intervention group showed reduced pain interference.  - Participants in the laptop group used ElderTree more frequently and reported more favorable perceptions than those in the smart display group. |
| Chiang et al (2020) [61] | Taiwan, China (High income) / Outpatient clinics of a medical center | To determine the effectiveness of a 12-week home-based telehealth exercise training program to increase physical activity, exercise capacity, and HRQoL in patients with cardiometabolic multimorbidity. | RCT | N = 50; Mean age = 60.0 years; 28% female; Multimorbidity profile: ≥2 cardiometabolic conditions (eg, hypertension, type 2 diabetes, hyperlipidemia, heart disease, metabolic syndrome, or gout). | **Home-based telehealth exercise training:** 36 individualized sessions (30 minutes, 3 times/week for 12 weeks) using heart rate–sensing smart clothing and a mobile app with real-time feedback, plus weekly telephone reminders and monthly outpatient follow-up. | Patient-Facing | Usual lifestyles + routine outpatient follow-up. | Baseline and 12 weeks; instruments: IPAQ, graded exercise testing (VO2peak, workload), SF-36. | - Significant group × time interactions were observed for total physical activity (β = 1333 MET-min/week, P = .004) and moderate-intensity physical activity (β = 330, P = .04).  - The intervention group showed a significant increase in VO2peak (β = 4.43, P = .04).  - Significant improvements were observed in physical function (β = 7.55, P = .03) and physical component summary score (β = 4.42, P = .03) of the SF-36. |
| Stewart et al (2021) [60] | Canada (High income) / 9 urban primary care sites (Ontario) | To assess the effectiveness of a provider-created, patient-centred, multi-provider case conference with follow-up (Telemedicine IMPACT Plus) for patients with multimorbidity. | Pragmatic RCT (mixed-methods) | N = 163; Mean age = 62.5 years; 65% female; Multimorbidity profile: ≥3 chronic conditions (mean approximately 6.0 conditions). | **Telemedicine IMPACT Plus (TIP):**Nurse-led planning + 1-1.5 hour multi-provider case conference (FP, internist, psychiatrist, social worker, PT, OT, pharmacist, dietitian, home care) focused on patient goals + 4 months nurse follow-up. | Clinician-Facing | Usual care plus a one-page list of community resources. | Baseline and 4 months; outcomes: heiQ, Self-Efficacy, VR-12, EQ-5D, Kessler-10, Health Behaviour Survey. | - No significant between-group differences were observed in the primary outcomes of self-management and self-efficacy.  - In subgroup analysis, the intervention improved mental health status among participants with annual income ≥C$50,000 (β = 11.0, P = .006).  - Qualitative findings suggested that patients valued the multidisciplinary team, felt supported, and appreciated the follow-up planning, although implementation experiences varied. |
| Panagioti et al (2018) [66] | UK (High income) / Primary care (North West England) | To evaluate the reach, effectiveness, and cost-effectiveness of telephone health coaching for older people with multimorbidity using a 'Trial within a Cohort' (TWiCs) design. | Trial within a Cohort (TWiCs) | N = 1,306 eligible participants; 504 were selected for intervention (207 consented; 41% uptake); Mean age = 74.7 years; 54.4% female; Multimorbidity profile: ≥2 long-term conditions. | **Proactive Telephone Coaching and Tailored Support (PROTECTS): 6** monthly telephone calls (~20 min) from a health advisor, focusing on health coaching (diet, exercise), social prescribing, and low-intensity support for low mood (BA, CBT). | Clinician-Facing | Usual NHS care. | Baseline, 6, 12, and 20 months; outcomes: Patient Activation Measure (PAM), WHOQOL-physical, MHI-5, SDSCA, EQ-5D-5L, healthcare utilization. | - No significant between-group differences were observed in the primary outcomes in the intention-to-treat analysis.  - Participants selected for the intervention had lower emergency care use but higher planned care use and higher overall costs.  - The incremental cost per QALY was £8049. |
| Wang et al (2025) [33] | China (Upper middle income) / Community health centers (2 counties, urban & rural) | To develop and assess the effectiveness of a community-based integrated care model for patients with diabetes and depression (CIC-PDD). | Cluster RCT (pragmatic) | N = 630; Mean age = 67.6 years; 70% female; Multimorbidity profile: all had type 2 diabetes and depression (PHQ-9 ≥10). | **Community-based Integrated Care for Diabetes and Depression (CIC-PDD):** Multi-professional team (specialist, PCP as case manager, health communicator), structured treatment plan, scheduled follow-up (6 & 12 months), enhanced interprofessional communication. | Clinician-Facing | Enhanced usual care (EUC): routine diabetes care + depression screening/referral. | Baseline, 6, and 12 months; outcomes: PHQ-9, HbA1c, SF-12, SDSCA, Morisky-8, MTBQ, PACIC-20. | - At 12 months, the intervention group showed significantly greater improvement in the primary outcomes, including both depressive symptoms and HbA1c.  - Significant improvements were also observed in mental quality of life, diabetes self-care, medication adherence, and patient experience of care.  - Rural participants benefited more from the intervention than urban participants. |
| Ye et al (2024) [34] | China (Upper middle income) / 900th Hospital of Joint Logistic Support Force | To investigate the effects of telehealth education (via WeChat) on glycolipid metabolism, blood pressure, and self-management in patients with coexisting T2DM and hypertension. | RCT | N = 174; 44.8% female; Multimorbidity profile: all had coexisting type 2 diabetes mellitus and hypertension. | **Telehealth education via WeChat:** daily posts on disease knowledge and self-management, question-and-answer support, lifestyle guidance, and peer communication delivered through a WeChat group over 26 weeks. | Patient-Facing | Conventional care (outpatient follow-up every 3 months, medication adjustment, diet/exercise education). | Baseline and 26 weeks; outcomes: weight, BMI, FBG, 2h-PBG, HbA1c, SBP/DBP, lipids, SDSCA. | - Compared with the control group, the telehealth education group showed significantly greater reductions in weight, body mass index, fasting blood glucose, 2-hour postprandial blood glucose, HbA1c, systolic blood pressure, and LDL-C.  - The telehealth education group also had significantly higher total SDSCA scores and better self-management across subdomains. |
| Araya et al (2021) [35] | Brazil (Upper middle income) / 20 primary care sites in São Paulo | To investigate the effectiveness of a digital intervention (CONEMO) in reducing depressive symptoms among people with hypertension and/or diabetes. | Cluster RCT | N = 880; Mean age = 56.0 years; 86.5% female; Multimorbidity profile: hypertension and/or diabetes plus depressive symptoms (PHQ-9 ≥10). | **CONEMO:** 18-session, low-intensity, smartphone-based behavioral activation intervention over 6 weeks, minimally supported by nurse assistants (initial meeting, 2 mandatory calls, additional calls for non-adherence). | Patient-Facing | Enhanced usual care (EUC): treatment as usual + safety net (risk assessment/referral). | Baseline, 3, and 6 months; primary outcome: ≥50% reduction in PHQ-9 at 3 months. | - At 3 months, the proportion of participants achieving a ≥50% reduction in PHQ-9 score was significantly higher in the intervention group than in enhanced usual care (40.7% vs 28.6%; adjusted OR = 1.6; P = .001).  - Effects were not sustained at 6 months. |
| Araya et al (2021) [35] | Peru (Upper middle income) / 7 outpatient sites in Lima | To investigate the effectiveness of a digital intervention (CONEMO) in reducing depressive symptoms among people with hypertension and/or diabetes. | Individual RCT | N = 432; Mean age = 59.7 years; 81.5% female; Multimorbidity profile: hypertension and/or diabetes plus depressive symptoms (PHQ-9 ≥10). | **CONEMO:** 18-session, low-intensity, smartphone-based behavioral activation intervention over 6 weeks, minimally supported by nurse assistants (initial meeting, 2 mandatory calls, additional calls for non-adherence). | Patient-Facing | Enhanced usual care (EUC): treatment as usual + safety net (risk assessment/referral). | Baseline, 3, and 6 months; primary outcome: ≥50% reduction in PHQ-9 at 3 months. | - At 3 months, the proportion of participants achieving a ≥50% reduction in PHQ-9 score was significantly higher in the intervention group than in enhanced usual care (52.7% vs 34.1%; adjusted OR = 2.1; P < .001).  - Effects were not sustained at 6 months. |
| O'Moore et al (2018) [50] | Australia (High income) / internet-delivered intervention in adults with knee osteoarthritis recruited through health care organizations | To determine the efficacy of an internet-based CBT (iCBT) program for depression in older adults with knee OA and comorbid major depressive disorder (MDD). | RCT | N = 69; Mean age = 62.0 years; 80% female; Multimorbidity profile: knee osteoarthritis plus major depressive disorder. | **Internet-based CBT for depression (Sadness Program):** 6 online lessons (CBT skills: psychoeducation, BA, cognitive restructuring, relapse prevention) over 10 weeks. Therapist support via email (and phone if deterioration). | Patient-Facing | Treatment as Usual (TAU): standard OA treatment (no structured psychological intervention). | Baseline, week 5, week 11 (post), and week 24 (3-month follow-up); outcomes: PHQ-9, K-10, ASES, WOMAC, SF-12, MINI. | - The iCBT group showed significantly greater improvement in depression symptoms and psychological distress than treatment as usual at postintervention and 3-month follow-up.  - Significant improvements were also observed in arthritis self-efficacy, pain, stiffness, and physical function at follow-up.  - Most iCBT participants no longer met diagnostic criteria for depression at 3-month follow-up. |
| Rifkin et al (2013) [45] | USA (High income) / VA CKD/Hypertension clinic | To test the effectiveness of a real-time, wireless BP monitoring intervention for older patients with CKD and hypertension on data exchange and BP control. | RCT | N = 43 analyzed; Mean age = 68 years; predominantly male; 26% Black; Multimorbidity profile: stage 3 or greater chronic kidney disease and uncontrolled hypertension. | **Wireless telemonitoring:** Bluetooth-enabled BP cuff + internet hub (cellular modem) transmitting readings automatically. Study physicians/pharmacist reviewed weekly and called if BP above goal. | Clinician-Facing | Usual care: home BP monitoring as recommended by physician, with no structured transmission or remote review. | Baseline and 6 months; outcomes: number of BP readings shared, BP change, medication changes, adherence (MMAS-8). | - The intervention group transmitted a median of 29 readings per month, whereas only 20% of controls brought blood pressure logs to visits.  - Systolic blood pressure improved in both groups; the between-group difference was not statistically significant (P for comparison = .31).  - Most intervention participants continued regular device use over 6 months, and device acceptability was high. |
| Schuffelen et al (2025) [49] | Germany (High income) / Online recruitment (nationwide) | To examine whether adding a fully automated digital CBT for insomnia (dCBT-I, somnio) to care-as-usual (CAU) improves depressive symptoms in patients with comorbid depression and insomnia. | RCT (pragmatic) | N = 140; Mean age = 39.76 years (SD = 11.65); 85.7% female; Multimorbidity profile: comorbid depression and insomnia. | **Digital CBT for Insomnia (dCBT-I, somnio):** 10 sequential core modules (psychoeducation, relaxation, stimulus control, bedtime restriction, cognitive therapy) delivered via mobile app or web platform over 12 weeks. Fully automated with avatar and reminders. | Patient-Facing | Waiting list control (WLC) + care-as-usual (CAU). WLC gained access after 24-week follow-up. | Baseline, 12 weeks (post), and 24 weeks (follow-up); outcomes: PHQ-9, ISI, ESS, FSS, WHO-5, PANAS, RSQ, PCS, sleep diary. | - Large treatment effects favored dCBT-I for depressive symptoms at 12 weeks and 24 weeks.  - Very large treatment effects favored dCBT-I for insomnia severity at both follow-up points.  - Significant improvements were also observed in fatigue, well-being, affect, and emotion regulation, but not daytime sleepiness. |
| Hsu et al (2021) [44] | Taiwan, China (High income) / Medical center (Kaohsiung Chang Gung Memorial Hospital) | To investigate the effects of diet control and telemedicine-based resistance exercise intervention on body composition, blood biochemistry, and functional performance in patients with obesity and knee OA. | RCT (3-arm) | N = 66; Multimorbidity profile: obesity and mild-to-moderate knee osteoarthritis. | **Three arms:** (D) Diet control (1200 kcal/day, dietitian follow-up via phone/app); (E) Elastic band resistance exercise (telemedicine supervision via phone/app); (D+E) Combined intervention. All 12 weeks. | Patient-Facing | Active comparators across 3 groups: diet control (D), resistance exercise (E), and combined diet control plus resistance exercise (D + E). | Baseline and 12 weeks; outcomes: body composition (BIA), WOMAC, TUG test, total cholesterol, LDL, triglycerides. | - The combined intervention group (D + E) showed the most consistent improvements in body composition, lower-limb functional performance, and blood biochemistry.  - Compared with the other groups, D + E achieved greater improvements in body fat percentage, timed up-and-go performance, WOMAC total score, and lipid outcomes.  - Diet-containing groups achieved significant weight loss, while the combined group preserved muscle mass better than diet control alone. |

**Abbreviations:** 2h-PBG, 2-hour postprandial blood glucose; 6MWT, 6-minute walk test; ACS, acute coronary syndrome; ACT, Acceptance and Commitment Therapy; ADLs, activities of daily living; AQoL-6D, Assessment of Quality of Life-6 Dimensions; AOU, Assessment of Underuse; ASES, Arthritis Self-Efficacy Scale; ASI, Anxiety Sensitivity Index; BADS-SF, Behavioral Activation for Depression Scale-Short Form; BATD, Behavioral Activation Therapy for Depression; BBQ, Brunnsviken Brief Quality of Life Questionnaire; BDI, Beck Depression Inventory; BG, blood glucose; BIA, bioelectrical impedance analysis; BIS, Bergen Insomnia Scale; BMI, body mass index; BP, blood pressure; BPI-IS, Brief Pain Inventory-Interference Scale; BPI-SF, Brief Pain Inventory-Short Form; C-MABS, Chinese Medication Adherence Behavior Scale; CAD, coronary artery disease; CAT, COPD Assessment Test; CAU, care as usual; CBP, chronic back pain; CBT, cognitive behavioral therapy; CDSS, clinical decision support system; CCPs, complex chronic patients; CHCs, community health centers; CHF, chronic heart failure; CI, confidence interval; CKD, chronic kidney disease; CLBP, chronic low back pain; CONEMO, Control Emocional; COPD, chronic obstructive pulmonary disease; CORE-10, Clinical Outcomes in Routine Evaluation-Outcome Measure 10; CPAQ, Chronic Pain Acceptance Questionnaire; CPAQ-8, 8-item Chronic Pain Acceptance Questionnaire; CSQ-R, Coping Strategies Questionnaire-Revised; DASS-21, Depression Anxiety Stress Scales-21; DBAS-10, 10-item Dysfunctional Beliefs and Attitudes about Sleep Scale; DBP, diastolic blood pressure; dCBT-I, digital cognitive behavioral therapy for insomnia; DCS, Decisional Conflict Scale; DDS, Diabetes Distress Scale; DHTL-AQ, Digital Health Technology Literacy Assessment Questionnaire; DKD, diabetic kidney disease; DSM-5, Diagnostic and Statistical Manual of Mental Disorders, Fifth Edition; ECG, electrocardiogram; ED, emergency department; eCDSS, electronic clinical decision support system; eCoach, electronic coach; eGFR, estimated glomerular filtration rate; EHR, electronic health record; EQ-5D, EuroQol 5-Dimension questionnaire; EQ-5D-5L, EuroQol 5-Dimension 5-Level; EQ-VAS, EuroQol Visual Analog Scale; ESS, Epworth Sleepiness Scale; ET, ElderTree; EUC, enhanced usual care; FBG, fasting blood glucose; FP, family physician; FPS-R, Faces Pain Scale-Revised; FSS, Fatigue Severity Scale; GAD-7, Generalized Anxiety Disorder-7; GAS, Goal Attainment Scaling; GDS, Geriatric Depression Scale; GFR, glomerular filtration rate; GOLD, Global Initiative for Chronic Obstructive Lung Disease; GP, general practitioner; HADS, Hospital Anxiety and Depression Scale; HAM-D, Hamilton Depression Rating Scale; HbA1c, glycated hemoglobin A1c; HBPM, home blood pressure monitoring; HDL-C, high-density lipoprotein cholesterol; heiQ, Health Education Impact Questionnaire; HF, heart failure; HR, hazard ratio; HRQoL, health-related quality of life; HRS-D, Hamilton Rating Scale for Depression; hsCRP, high-sensitivity C-reactive protein; HTN, hypertension; I-TEAM, Integrated Telehealth Education and Activation of Mood; IAR, internet-based applied relaxation; IASP, International Association for the Study of Pain; iCBT, internet-based cognitive behavioral therapy; ICBT-i, internet-based cognitive behavioral therapy for insomnia; IG, intervention group; IL-6, interleukin-6; IMI, internet- and mobile-based intervention; IPAQ, International Physical Activity Questionnaire; ISI, Insomnia Severity Index; iTAU, improved treatment as usual; K-10, Kessler Psychological Distress Scale; K&L, Kellgren-Lawrence; KCCQ-12, Kansas City Cardiomyopathy Questionnaire-12; LACE, Length of stay, Acuity of admission, Comorbidity, and Emergency department use; LDL-C, low-density lipoprotein cholesterol; MADRS-S, Montgomery-Åsberg Depression Rating Scale-Self-rated; MAI, Medication Appropriateness Index; MCS-12, 12-item Mental Component Summary; MDD, major depressive disorder; MHI-5, Mental Health Inventory-5; MINI, Mini-International Neuropsychiatric Interview; MLHFQ, Minnesota Living with Heart Failure Questionnaire; MMAS-8, 8-item Morisky Medication Adherence Scale; MOS-SSS, Medical Outcomes Study Social Support Survey; MRC, Medical Research Council dyspnea scale; MTBQ, Multimorbidity Treatment Burden Questionnaire; NRS, Numeric Rating Scale; NYHA, New York Heart Association; OA, osteoarthritis; OBPM, office blood pressure monitoring; ODI, Oswestry Disability Index; OFS, Openness to the Future Scale; OR, odds ratio; OT, occupational therapist; PACIC, Patient Assessment of Chronic Illness Care; PAD, peripheral artery disease; PAM, Patient Activation Measure; PANAS, Positive and Negative Affect Schedule; PASE, Physical Activity Scale for the Elderly; PCS, Pain Catastrophizing Scale; PCS-12, 12-item Physical Component Summary; PDI, Pain Disability Index; PHQ-9, 9-item Patient Health Questionnaire; PIPS, Psychological Inflexibility in Pain Scale; PROMIS-D, Patient-Reported Outcomes Measurement Information System Depression; PSEQ, Pain Self-Efficacy Questionnaire; PSEQ-2, 2-item Pain Self-Efficacy Questionnaire; PT, physiotherapist; PTSD, post-traumatic stress disorder; QALY, quality-adjusted life year; QIDS, Quick Inventory of Depressive Symptomatology; QoL, quality of life; QOLI, Quality of Life Inventory; RAAS, renin-angiotensin-aldosterone system; RCT, randomized controlled trial; RD, risk difference; RMDQ, Roland-Morris Disability Questionnaire; RR, risk ratio; RSQ, Response Style Questionnaire; SBP, systolic blood pressure; SC-CII, Self-Care of Chronic Illness Inventory; SCID, Structured Clinical Interview for DSM Disorders; SDSCA, Summary of Diabetes Self-Care Activities; SF-12, 12-Item Short Form Health Survey; SF-36, 36-Item Short Form Health Survey; SMBG, self-monitoring of blood glucose; SMS, short message service; SMP-T2D, Self-Management Profile for Type 2 Diabetes; SPSI-R, Social Problem-Solving Inventory-Revised; STOPP/START, Screening Tool of Older Persons’ Prescriptions/Screening Tool to Alert to Right Treatment; STRIPA, STRIP Assistant; T2DM, type 2 diabetes mellitus; TAU, treatment as usual; TC, total cholesterol; TG, triglycerides; TSK-11, Tampa Scale of Kinesiophobia-11; TSN, technological surrogate nursing; TUG, timed up-and-go test; TWiCs, Trials within Cohorts; UC+P, usual care plus psychoeducation; UCDC, Ubiquitous Chronic Disease Care system; VA, Veterans Affairs; VO₂peak, peak oxygen uptake; VR-12, Veterans RAND 12-Item Health Survey; baPWV, brachial-ankle pulse wave velocity; WLC, waiting-list control; WOMAC, Western Ontario and McMaster Universities Osteoarthritis Index; WSAS, Work and Social Adjustment Scale.

**Note:** Araya et al. reported 2 parallel trials in Brazil and Peru; these are presented as separate trial entries because the design and setting differed, but they represent 1 report. This table summarizes the key characteristics of the included randomized controlled trials evaluating digital health interventions for adults with multimorbidity. Data were extracted independently by 2 reviewers using a standardized template adapted from the Cochrane Consumers and Communication Review Group data extraction template for included studies. References for each study are provided in the main reference list.
