# Supplementary material for: Effectiveness and Implementation of Digital Health Interventions on Physiological, Psychological, and Functional Outcomes in Adults With Multimorbidity: Systematic Review and Meta-Analysis of Randomized Controlled Trials
Source: J Med Internet Res. 2026 Jul 28;28:e90458. doi: 10.2196/90458 (PMC13412019; doi:10.2196/90458)
Supplement: Multimedia Appendix 5 [file jmir-v28-e90458-s005.docx]

**Table S1.** Risk of bias of included studies assessed using RoB 2 for the main outcome of each study.

| **No.** | **Author (year)** | **D1** | **D2** | **D3** | **D4** | **D5** | **Overall** |
| --- | --- | --- | --- | --- | --- | --- | --- |
| 1 | González-Ortega et al (2017) [65] | Low | Some concerns | Low | Some concerns | Low | Some concerns |
| 2 | Chan et al (2022) [36] | Low | Some concerns | Low | Low | Low | Some concerns |
| 3 | Baumeister et al (2021) [48] | Low | Some concerns | Low | Low | Low | Some concerns |
| 4 | Liang et al (2021) [57] | Low | Some concerns | High | Low | Low | High |
| 5 | Wakefield et al (2011) [41] | Low | Some concerns | Some concerns | Low | Low | Some concerns |
| 6 | Tchalla et al (2025) [58] | Some concerns | Some concerns | Low | Low | Low | Some concerns |
| 7 | Yao et al (2021) [31] | Some concerns | Some concerns | Low | Low | Low | Some concerns |
| 8 | Monreal-Bartolomé et al (2025) [52] | Low | Some concerns | High | Some concerns | Low | High |
| 9 | Rollman et al (2021) [40] | Low | Some concerns | Low | Some concerns | Low | Some concerns |
| 10 | Gustafson et al (2024) [62] | Low | Some concerns | Low | Some concerns | Low | Some concerns |
| 11 | Bernocchi et al (2018) [56] | Low | Some concerns | High | Some concerns | Low | High |
| 12 | Mihevc et al (2025) [55] | Low | Some concerns | Low | Low | Low | Some concerns |
| 13 | Yu et al (2020) [37] | Some concerns | Some concerns | Low | Some concerns | Low | Some concerns |
| 14 | Jungo et al (2023) [38] | Low | Some concerns | Low | Low | Low | Some concerns |
| 15 | Blum et al (2021) [39] | Low | Some concerns | Low | Low | Low | Some concerns |
| 16 | Clarke et al (2019) [63] | Low | Some concerns | High | Some concerns | Low | High |
| 17 | Lear et al (2021) [64] | Low | Some concerns | Low | Low | Low | Some concerns |
| 18 | Prabhakaran et al (2019) [32] | Some concerns | Some concerns | Low | Low | Low | Some concerns |
| 19 | Yoo et al (2009) [53] | Some concerns | Some concerns | Low | Some concerns | Low | Some concerns |
| 20 | Gellis et al (2014) [59] | Low | Some concerns | Low | Some concerns | Low | Some concerns |
| 21 | Bothelius et al (2024) [47] | Low | Some concerns | High | Some concerns | Low | High |
| 22 | Sanabria-Mazo et al (2023) [42] | Low | Some concerns | High | Some concerns | Low | High |
| 23 | Or et al (2020) [54] | Low | Some concerns | Low | Low | Low | Some concerns |
| 24 | Gasslander et al (2022) [46] | Low | Some concerns | High | Some concerns | Low | High |
| 25 | Hwang et al (2025) [51] | Low | Some concerns | Low | Some concerns | Low | Some concerns |
| 26 | Landucci et al (2025) [43] | Low | Some concerns | Low | Some concerns | Low | Some concerns |
| 27 | Chiang et al (2020) [61] | Low | Some concerns | Low | Low | Low | Some concerns |
| 28 | Stewart et al (2021) [60] | Low | Some concerns | Low | Low | Low | Some concerns |
| 29 | Panagioti et al (2018) [66] | Low | Some concerns | Low | Low | Low | Some concerns |
| 30 | Wang et al (2025) [33] | Some concerns | Some concerns | Low | Low | Low | Some concerns |
| 31 | Ye et al (2024) [34] | Some concerns | Some concerns | Low | Low | Low | Some concerns |
| 32 | Araya et al (2021) [35] | Some concerns | Some concerns | Low | Some concerns | Low | Some concerns |
| 33 | O'Moore et al (2018) [50] | Low | Some concerns | Low | Some concerns | Low | Some concerns |
| 34 | Rifkin et al (2013) [45] | Some concerns | Some concerns | Low | Low | Low | Some concerns |
| 35 | Schuffelen et al (2025) [49] | Low | Some concerns | Low | Some concerns | Low | Some concerns |
| 36 | Hsu et al (2021) [44] | Low | Some concerns | Low | Some concerns | Low | Some concerns |

**Note:** Judgments were made using the revised Cochrane Risk of Bias tool for randomized trials (RoB 2). For studies reporting multiple primary or co-primary outcomes, judgments were based on the main outcome specified by trial authors when available; otherwise, the most clinically relevant main outcome for this review was used. Araya 2021 contained 2 trials (Brazil cluster-randomized trial and Peru individually randomized trial) and is displayed as a combined judgment. For Ye 2024, D4 was judged on the basis of HbA1c as the main objective outcome.

**Table S2. Summary of RoB 2 judgments across domains**

| **Domain** | **Low risk, n (%)** | **Some concerns, n (%)** | **High risk, n (%)** |
| --- | --- | --- | --- |
| D1 randomization process | 27 (75.0) | 9 (25.0) | 0 (0.0) |
| D2 deviations from intended interventions | 0 (0.0) | 36 (100.0) | 0 (0.0) |
| D3 missing outcome data | 28 (77.8) | 1 (2.8) | 7 (19.4) |
| D4 measurement of the outcome | 18 (50.0) | 18 (50.0) | 0 (0.0) |
| D5 selection of the reported result | 36 (100.0) | 0 (0.0) | 0 (0.0) |
| Overall bias | 0 (0.0) | 29 (80.6) | 7 (19.4) |

**Abbreviations:** D1, bias arising from the randomization process; D2, bias due to deviations from intended interventions; D3, bias due to missing outcome data; D4, bias in measurement of the outcome; D5, bias in selection of the reported result
