# Supplementary material for: Effectiveness and Implementation of Digital Health Interventions on Physiological, Psychological, and Functional Outcomes in Adults With Multimorbidity: Systematic Review and Meta-Analysis of Randomized Controlled Trials
Source: J Med Internet Res. 2026 Jul 28;28:e90458. doi: 10.2196/90458 (PMC13412019; doi:10.2196/90458)
Supplement: Multimedia Appendix 6 [file jmir-v28-e90458-s006.docx]

**Table S1**. Study-level results for additional outcomes, alternative time points, and trial-reported analyses not used as main pooled effect estimates

| **Study** | **Outcome** | **Time point** | **Intervention group result** | **Comparator group result** | **Sample size (I/C)** | **Between-group estimate or statistical result** | **Study-reported interpretation** |  |  |  |  |  |  |  |
| --- | --- | --- | --- | --- | --- | --- | --- | --- | --- | --- | --- | --- | --- | --- |
| **Glycemic and multimarker disease-control outcomes** | | | | | | | |  |  |  |  |  |  |  |
| Clarke et al (2019) [63] | SMP-T2D Blood Glucose Monitoring | 3 months | 49.61→49.25 | 45.55→55.83 | 232/241 | *β*=8.98, *P*=.006 | Control improved more |  |  |  |  |  |  |  |
|  | Fasting BG (mmol/l) | 12 months | -1.2 (-1.9 to -0.5) | -0.4 (-1.0 to 0.2) | 55/62 | Diff -0.8 (-1.4 to -0.2), adj *P*=.06 | Not significant after adjustment |  |  |  |  |  |  |  |
| Wakefield et al (2011) [41] | HbA1c change | 6 months | High: -0.44; Low: -0.40 | -0.07 | 93 / 102 / 107 | High vs control *P*=.02; Low vs control *P*=.03 | Both intervention groups reduced HbA1c |  |  |  |  |  |  |  |
|  | HbA1c (12 months) | 12 months | High: -0.19; Low: -0.17 | -0.33 | 93/102/107 | *P*=.65 between groups | No sustained difference |  |  |  |  |  |  |  |
| **Lipid and metabolic biomarker outcomes** | | | | | | | |  |  |  |  |  |  |  |
| Chan et al (2022) [36] (JADE) | ≥3 treatment targets (HbA1c, BP, LDL, TG, RAASi) | 12 months | 355/796 (44.6%) | Usual:304/795(38.2%); Empowered:286/802(35.7%) | 796 / 795 / 802 | Team vs Usual: RR 1.17 (1.00-1.37); vs Empowered: RR 1.25 (1.06-1.48) | Team-based empowered care improved target attainment |  |  |  |  |  |  |  |
|  | LDL-cholesterol change (mmol/L) | 12 months | -0.14 (1.35) | Usual: 0.02 (1.11); Empowered: 0.09 (1.47) | 796/795/802 | *P*<.001 vs usual | Significant LDL reduction in team-based arm |  |  |  |  |  |  |  |
| Mihevc et al (2025) [55] | LDL-cholesterol | 12 months | -0.1 (-0.3 to 0.1) | +0.2 (0.0 to 0.4) | 55/62 | Diff 0.3 (0.1-0.6), adj *P*>.05 | Not significant after multiple comparison correction |  |  |  |  |  |  |  |
| Prabhakaran et al (2019) [32] | Total cholesterol (mg/dL) | 12 months | 0.1 | 2.0 | 1842/1856 | Adj diff -2.5 (-7.1-2.0), *P*=.29 | No difference |  |  |  |  |  |  |  |
| Yoo et al (2009) [53] | Total cholesterol (mmol/L) | 12 weeks | 4.6 → 4.1 | 4.5 → 4.5 | 57/54 | *P*<.001 for IG | Improved lipids |  |  |  |  |  |  |  |
|  | LDL-cholesterol (mmol/L) | 12 weeks | 2.6 → 2.2 | 2.4 → 2.3 | 57/54 | *P*<.001 for IG | Improved LDL |  |  |  |  |  |  |  |
|  | Adiponectin (μg/ml) | 12 weeks | 2.9 → 7.5 | 3.1 → 3.5 | 57/54 | *P*<.001 for IG | Increased adiponectin |  |  |  |  |  |  |  |
| **Blood pressure and cardiovascular risk outcomes** | | | | | | | |  |  |  |  |  |  |  |
|  |  |  |  |  |  |  |  | CVD risk score (%) | 12 months | 2.4 | 0.6 | 1842/1856 | Adj diff -0.4 (-2.3-1.5), *P*=.66 | No difference |
| Wakefield et al (2011) [41] | SBP change | 6 months | High: -6.05; Low: -0.29 | +4.48 | 93/102/107 | High vs control *P*<.001; high vs low *P*=.06 | High-intensity group reduced SBP |  |  |  |  |  |  |  |
|  | SBP (12 months) | 12 months | High: -4.92; Low: +0.76 | +3.34 | 93/102/107 | High vs control *P*=.006; high vs low *P*=.08 | SBP reduction maintained in high-intensity group |  |  |  |  |  |  |  |
| Yao et al (2021) [31] (mAFA-II) | Composite of ACS, HF, uncontrolled BP (C criterion) | ~12 months | 27/833 (3.2%) | 145/1057 (13.7%) | 833/1057 | HR 0.29 (0.19-0.45), *P*<.001 | Improved comorbidity management |  |  |  |  |  |  |  |
| **Mental health and psychosocial outcomes** | | | | | | | |  |  |  |  |  |  |  |
|  |  |  |  |  |  |  |  | HAM-D (6-month) | 6 months | 8.97 (6.44) | 9.95 (7.13) | 104/105 | *β*=-0.14 (95% CI -0.40 to 0.12), *P*=.283 | Non-significant |
|  |  |  |  |  |  |  |  | QIDS (depression) | 9 weeks | 12.12→7.29 | 12.24→8.56 | 104/105 | *β*=-0.27 (95% CI -0.52 to -0.01), *P*=.038 | Significant improvement in IG |
|  |  |  |  |  |  |  |  | PHQ-9 (self-report) | 9 weeks | 12.69→8.92 | 13.00→11.29 | 104/105 | *β*=-0.40 (95% CI -0.61 to -0.19), *P*<.001 | IG significantly better |
| Bothelius et al (2024) [47] | Insomnia Severity Index (ISI) | 9 weeks | 19.1 → 14.1 (d=1.49) | 19.2 → 13.6 (d=2.00) | 42 / 43 | Between-group *d*=0.14, non-sig | Both groups improved, no specific effect |  |  |  |  |  |  |  |
|  | Bergen Insomnia Scale (BIS) | 9 weeks | 28.6 → 20.8 (d=0.90) | 27.0 → 19.6 (d=0.82) | 42/43 | Time effect *P*=.019, group×time n.s. | Both improved |  |  |  |  |  |  |  |
|  | Anxiety (GAD-7) | 9 weeks | 7.7 → 7.2 | 6.8 → 3.9 | 42/43 | Group×time *P*=.01, favoring IAR | IAR better for anxiety |  |  |  |  |  |  |  |
|  | GAD-7 (anxiety) | 3 months | 7.60→6.66 | 7.33→6.20 | 232/241 | *β*=-0.276, *P*=.46 | Both improved |  |  |  |  |  |  |  |
|  | Diabetes Distress Scale | 3 months | 2.56→2.24 | 2.56→2.18 | 232/241 | *β*=-0.062, *P*=.36 | Both improved |  |  |  |  |  |  |  |
|  | HADS (emotional distress) | 3 months | 17.52 → 15.65 | 16.61 → 17.23 | 95/92 | *d*=0.19, *P*=.003 | Reduced distress |  |  |  |  |  |  |  |
| Gellis et al (2014) [59] (I-TEAM) | HAM-D (depression) | 3 months | 18.1 → 9.8 | 17.8 → 18.6 | 46 / 48 | *P*=.02 | 50% lower depression in IG |  |  |  |  |  |  |  |
|  | SF-12 Mental Component | 3 months | 39.7 → 53.6 | 39.4 → 42.8 | 46/48 | *P*=.01 | Improved mental QoL |  |  |  |  |  |  |  |
| González-Ortega et al (2017) [65] | SF-12 Mental Component | 6 months | 43.9 → 41.7 (P=.406) | 42.0 → 42.4 (P=.169) | 71 / 79 | Mean diff 2.60 (95% CI -3.90 to 9.11), *P*=.424 | No significant difference |  |  |  |  |  |  |  |
| Gustafson et al (2024) [62] | Mental QoL (PROMIS) | 12 months | 46.25→↑ | →↓ | 176 / 168 | Arm×time *β*=0.76 (0.14-1.37), *P*=.02; 12-month diff 1.5 T-score (*d*=0.15) | Small improvement in mental QoL overall |  |  |  |  |  |  |  |
|  | Psychological well-being | 12 months | 31.33→ | — | 176/168 | *β*=0.23 (-0.26-0.72), *P*=.36 | No significant difference |  |  |  |  |  |  |  |
|  | Loneliness (UCLA) | 12 months | 16.59→ | — | 176/168 | *β*=-0.25 (-0.73-0.22), *P*=.29 | No effect on loneliness |  |  |  |  |  |  |  |
|  | Mental QoL (sex moderation) | 12 months | Women: diff 2.60 (P=.002); Men: diff -0.06 (P=.95) | — | 177W/91M | Sex×arm×time *β*=1.33 (0.09-2.58), *P*=.04 | Moderation finding suggesting stronger benefit among women |  |  |  |  |  |  |  |
|  | Psychological well-being (sex moderation) | 12 months | Women: diff 1.38 (P=.04); Men: diff 0.88 (P=.28) | — | 177W/91M | Sex×arm×time *β*=1.13 (0.13-2.12), *P*=.03 | Moderation finding suggesting stronger benefit among women |  |  |  |  |  |  |  |
| Hwang et al (2025) [51] | Health distress | 8 weeks | 1.88 → 1.53 | 1.78 → 2.17 | 21 / 21 | Group×time χ²=3.89, *P*=.049 | Improved health distress |  |  |  |  |  |  |  |
|  | Depression (PHQ-8) | 8 weeks | 4.35 → 3.78 | 4.03 → 6.31 | 21/21 | Group×time χ²=4.99, *P*=.025 | Improved depression |  |  |  |  |  |  |  |
|  | PANAS positive affect | 3 months | Improved | — | 93/90 | *B* = 4.73 (2.01-7.45), *P*<.05 | Increased positive affect |  |  |  |  |  |  |  |
|  | PANAS negative affect | 3 months | Reduced | — | 93/90 | *B* = -3.67 (-6.63 to -0.71), *P*<.05 | Decreased negative affect |  |  |  |  |  |  |  |
|  | Openness to future (OFS) | 3 months | Improved | — | 93/90 | *B* = 4.73 (2.01-7.45), *P*<.05 | Increased openness to future |  |  |  |  |  |  |  |
| Panagioti et al (2018) [66] | Depression (MHI-5) | 20 months | 75.74 | 74.29 | 325/583 | Adj diff 1.00 (-1.25-3.26), *P*=.37 | No effect |  |  |  |  |  |  |  |
| Prabhakaran et al (2019) [32] | Depression score | 12 months | 10.9 | 12.4 | 1842/1856 | Adj diff -1.6 (-4.4-1.2), *P*=.28 | No difference |  |  |  |  |  |  |  |
| Rollman et al (2021) [40] | MCS-12 (mental HRQoL) | 12 months | Blended care: baseline 40.1; estimated 12-month score approximately 44.6 | eUC: baseline 40.2; estimated 12-month score approximately 41.3; UC: baseline 39.9; estimated 12-month score approximately 42.5 | 251/252/126 | Blended vs UC: adjusted difference 4.47 (95% CI 1.65 to 7.28), *P*=.002; blended vs eUC: adjusted difference 1.12 (95% CI −1.15 to 3.40), *P*=.33 | Blended care improved MCS-12 versus UC, but not versus eUC. |  |  |  |  |  |  |  |
|  | PROMIS-Depression | 12 months | Blended care: baseline 60.3; 12-month score approximately 53.8 | eUC: baseline 60.1; 12-month score approximately 56.9; UC: baseline 60.1; 12-month score approximately 58.1 | 251/252/126 | Blended vs UC: ES 0.47 (95% CI 0.28 to 0.67), *P*<.001; blended vs eUC: ES 0.24 (95% CI 0.07 to 0.41), *P*=.006 | Blended care improved mood versus both UC and eUC. |  |  |  |  |  |  |  |
|  | HRS-D (depression) | 12 months | Not separately reported in the main text for the full cohort | Not separately reported in the main text for the full cohort | 251/252/126 | No overall differential improvement was reported; blended vs UC showed improvement only among women (ES 0.35, 95% CI 0.02 to 0.67; *P*=.04). | Subgroup finding in women only; HRS-D did not show a consistent overall treatment effect. |  |  |  |  |  |  |  |
| Sanabria-Mazo et al (2023) [42] | Stress (DASS-21) ACT vs TAU | 2 months | 9.31 → 6.04 | 8.82 → 8.91 | 78/78 | *d*=0.69, *P*<.001 | ACT reduced stress |  |  |  |  |  |  |  |
|  | Depression (DASS-21) | 2 months | No significant difference | — | 78/78 | *P*>.05 | No effect on depression |  |  |  |  |  |  |  |
|  | Anxiety (DASS-21) | 2 months | No significant difference | — | 78/78 | *P*>.05 | No effect on anxiety |  |  |  |  |  |  |  |
| Stewart et al (2021) [60] (TIP) | SF-12 Mental Component | 4 months | 42.66 → 43.86 | 44.01 → 46.23 | 86/77 | *β*=-1.402 (-5.06-2.25), *P*=.45 | No effect overall |  |  |  |  |  |  |  |
| Yu et al (2020) [37] | Diabetes Distress Scale (DDS) | 12 months | 2.08 → 1.86 | 1.93 → 1.90 | 102 / 111 | Mean diff -0.18 (-0.42 to 0.05), *P*=.12 | No significant difference |  |  |  |  |  |  |  |
| **Pain, physical function, and exercise-capacity outcomes** | | | | | | | |  |  |  |  |  |  |  |
| Baumeister et al (2021) [48] | Pain intensity (NRS) | 9 weeks | 1.88→1.43 | 1.78→1.63 | 104/105 | *β*=-0.32 (-0.57 to -0.06), *P*=.013 | Reduced pain intensity in IG |  |  |  |  |  |  |  |
|  | Pain self-efficacy (PSEQ) | 9 weeks | 28.08→35.74 | 32.38→35.04 | 104/105 | *β*=0.33 (0.15-0.51), *P*<.001 | Self-efficacy improved |  |  |  |  |  |  |  |
| Bernocchi et al (2018) [56] | 6MWT (exercise tolerance) | 4 months | Δ +60m (22.2 to 97.8) | Δ -15m (-40.3 to 9.8) | 56 / 56 | Between-group *P*=.004 | Significant improvement in IG |  |  |  |  |  |  |  |
|  | MRC dyspnea | 4 months | Δ -0.17 (-0.3 to -0.02) | Δ 0.07 (-0.1 to 0.3) | 56/56 | *P*=.05 | Reduced dyspnea |  |  |  |  |  |  |  |
|  | PASE (physical activity) | 4 months | Δ +18.1 (-0.6 to 36.9) | Δ -21.3 (-35.7 to -7.0) | 56/56 | *P*=.002 | Improved physical activity profile |  |  |  |  |  |  |  |
|  | Barthel Index | 4 months | Δ +5.4 (3.6 to 7.2) | Δ +1.3 (-0.2 to 2.8) | 56/56 | *P*<.001 | Improved disability |  |  |  |  |  |  |  |
|  | CAT (COPD assessment) | 4 months | Δ -5.3 (-6.9 to -3.7) | Δ +1.6 (-0.4 to 3.5) | 56/56 | *P*<.001 | Improved COPD status |  |  |  |  |  |  |  |
|  | 6MWT (6-month maintenance) | 6 months | Benefits maintained | Further decline (-43m) | 56/56 | ANOVA time×group *P*<.001 | Between-group difference over time remained significant at follow-up |  |  |  |  |  |  |  |
| Blum et al (2021) [39] (OPERAM) | ADL (Barthel) | 12 months | 89.0 | 86.9 | 665/713 | MD 1.60 (-0.70-3.89), *P*=.17 | No difference |  |  |  |  |  |  |  |
| Bothelius et al (2024) [47] | Pain interference (BPI) | 9 weeks | 43.3 → 42.2 | 47.0 → 36.3 | 42/43 | Group×time *P*=.003, favoring IAR | IAR (relaxation) better for pain interference |  |  |  |  |  |  |  |
| Chiang et al (2020) [61] | VO2peak (mL/kg/min) | 12 weeks | +4.43 (0.31-8.56) | +0.03 | 25 / 25 | *P*=.03 | Improved cardiorespiratory fitness |  |  |  |  |  |  |  |
|  | Workload (Watt) | 12 weeks | 116 (post) | 88.7 (post) | 25/25 | *β*=16.7 (-1.33-34.7), *P*=.06 | Trend toward improvement |  |  |  |  |  |  |  |
|  | Total PA (MET/wk) | 12 weeks | +1333 (342-2241) | +283 | 25/25 | *P*=.004 | Increased PA |  |  |  |  |  |  |  |
|  | Moderate PA (MET/wk) | 12 weeks | +331 (15-656) | +150 | 25/25 | *P*=.04 | Increased moderate PA |  |  |  |  |  |  |  |
|  | SF-36 Physical Function | 12 weeks | +7.55 (0.84-14.3) | -0.64 | 25/25 | *P*=.03 | Improved physical function |  |  |  |  |  |  |  |
|  | Pain acceptance (CPAQ) | 3 months | 51.49 → 58.05 | 50.59 → 52.16 | 95/92 | *d*=0.30, *P*=.005 | Increased pain acceptance |  |  |  |  |  |  |  |
| González-Ortega et al (2017) [65] | Barthel Index | 6 months | 78.9 → 76.3 (P=.01) | 83.0 → 80.7 (P=.009) | 71 / 79 | Mean diff 0.33 (95% CI -2.24 to 2.90), *P*=.801 | Functional decline in both groups, no between-group diff |  |  |  |  |  |  |  |
| Landucci et al (2025) [43] | Pain interference (PROMIS) | 8 months | Laptop+SD vs control: n.s. | — | 177 / 91 | Arm×time *β*=-0.41 (-1.23-0.41), *P*=.33 | No overall effect |  |  |  |  |  |  |  |
|  | Pain interference (women) | 8 months | Combined intervention: significant within-group decrease among women (P=.02) | Control: no significant change | Women subgroup | Gender moderation on change over time, *P*=.04; no significant between-arm difference at 8 months | Gender moderated change over time; no significant between-arm difference at 8 months |  |  |  |  |  |  |  |
| Liang et al (2021) [57] | ADL (Barthel) | 6 months | 48.96→53.91 | 44.09→49.04 | 91/76 | GEE *β*=4.951, *P*=.217 | No difference |  |  |  |  |  |  |  |
| Monreal-Bartolomé et al (2025) [52] | Pain intensity / disability | 3 months | No significant change | — | Subgroup | *P*=.79 / *P*=.43 | No improvement in pain outcomes |  |  |  |  |  |  |  |
| Rollman et al (2021) [40] | KCCQ-12 (HF physical function) | 12 months | Blended care: baseline 40.9; estimated 12-month score approximately 52.4 | eUC: baseline 39.8; estimated 12-month score approximately 51.0; UC: baseline 41.4; estimated 12-month score approximately 52.6 | 251/252/126 | No significant between-group difference was reported. | Physical function was similar across randomized arms. |  |  |  |  |  |  |  |
| Sanabria-Mazo et al (2023) [42] | Pain interference (BPI-IS) ACT vs TAU | 2 months (post) | 6.71 → 4.89 | 6.49 → 5.84 | 78 / 78 | *d*=0.64, *P*<.001 | ACT reduced pain interference |  |  |  |  |  |  |  |
|  | Pain interference (BPI-IS) BATD vs TAU | 12 months (FU) | 6.46 → 5.07 | 6.49 → 6.42 | 78/78 | *d*=0.66, *P*<.001 | BATD effective at follow-up |  |  |  |  |  |  |  |
|  | Pain catastrophizing (PCS) ACT | 2 months | 24.88 → 17.29 | 24.14 → 22.09 | 78/78 | *d*=0.45, *P*=.014 | Reduced catastrophizing |  |  |  |  |  |  |  |
|  | Pain catastrophizing (PCS) BATD | 2 months | 24.22 → 14.83 | 24.14 → 22.09 | 78/78 | *d*=0.59, *P*<.001 | Reduced catastrophizing |  |  |  |  |  |  |  |
|  | Pain acceptance (CPAQ-8) ACT | 2 months | 18.08 → 20.27 | 18.53 → 18.36 | 78/78 | *d*=0.34, *P*=.003 | Increased pain acceptance |  |  |  |  |  |  |  |
| **Quality-of-life and perceived-health outcomes** | | | | | | | |  |  |  |  |  |  |  |
| Baumeister et al (2021) [48] | Health-related QoL (AQoL-6D) | 9 weeks | 54.90→48.32 | 54.04→51.20 | 104/105 | *β*=-0.36 (-0.55 to -0.18), *P*<.001 | Better QoL in IG |  |  |  |  |  |  |  |
|  | AQoL-6D (6 months) | 6 months | 47.45 | 49.50 | 104/105 | *β*=-0.28 (-0.47 to -0.08), *P*=.006 | Effect maintained |  |  |  |  |  |  |  |
| Bernocchi et al (2018) [56] | MLHFQ (QoL) | 4 months | Δ -10.5 (-14.2 to -6.8) | Δ -0.44 (-4.9 to 4.0) | 56/56 | *P*<.001 | Improved QoL |  |  |  |  |  |  |  |
| Blum et al (2021) [39] (OPERAM) | Quality of life (EQ-VAS) | 12 months | 67.1 | 65.1 | 657/703 | MD 2.29 (0.31-4.26), *P*=.02 | Small improvement in EQ-VAS in the intervention group |  |  |  |  |  |  |  |
| Gasslander et al (2022) [46] | Quality of Life (QOLI) | 3 months | Improved | — | 95/92 | *d*=0.02, *P*<.001 | Small QoL improvement |  |  |  |  |  |  |  |
| González-Ortega et al (2017) [65] | SF-12 Physical Component | 6 months | 33.6 → 36.1 (P=.834) | 35.2 → 33.0 (P=.082) | 71 / 79 | Mean diff 4.71 (95% CI -9.03 to -0.41), *P*=.033 [reported as published; CI appears internally inconsistent in source] | Reported improvement in physical component; interpret cautiously because the published CI appears internally inconsistent |  |  |  |  |  |  |  |
| Gustafson et al (2024) [62] | Physical QoL (PROMIS) | 12 months | 42.39→ | — | 176/168 | *β*=0.10 (-0.44-0.64), *P*=.71 | No effect on physical QoL |  |  |  |  |  |  |  |
| Jungo et al (2023) [38] (OPTICA) | EQ-5D-5L utilities | 12 months | 0.1 (inv pred) | 0.1 | 160/163 | MD 0.00 (-0.04-0.03), *P*=.93 | No difference |  |  |  |  |  |  |  |
|  | EQ-VAS | 12 months | 72 | 73 | 160/163 | MD -0.42 (-3.77-2.93), *P*=.81 | No difference |  |  |  |  |  |  |  |
| Landucci et al (2025) [43] | Psychosocial QoL (PROMIS) | 8 months | Laptop+SD vs control: n.s. | — | 177/91 | *β*=0.04 (-0.61-0.69), *P*=.9 | No overall effect |  |  |  |  |  |  |  |
|  | Psychosocial QoL (men) | 8 months | Combined intervention: significant within-group decrease among men (P=.02) | Control: stable | Men subgroup | Gender moderation on change over time, *P*=.03; changes did not reach the benchmark for meaningful difference | Negative change over time in men; not clinically meaningful |  |  |  |  |  |  |  |
| Liang et al (2021) [57] | QoL (EQ-5D-5L) | 6 months | 14.47→16.86 | 15.69→15.69 | 91/76 | GEE *β*=2.391, *P*=.003 | Improved QoL in IG |  |  |  |  |  |  |  |
| Mihevc et al (2025) [55] | Diabetes-related QoL (ADS) | 12 months | -0.4 (-1.0 to 0.2) | 0.0 (-0.6 to 0.6) | 55/62 | No significant difference | No effect on QoL |  |  |  |  |  |  |  |
| Monreal-Bartolomé et al (2025) [52] | SF-12 (perceived health) | 3 months | Improved | — | 93/90 | *B* = 9.04 (3.21-14.87), *P*<.05 | Improved perceived health |  |  |  |  |  |  |  |
| Panagioti et al (2018) [66] | WHOQOL Physical Health | 20 months | 55.74 | 55.41 | 327/577 | Adj diff 1.62 (-0.32-3.56), *P*=.1 | No effect |  |  |  |  |  |  |  |
| Rollman et al (2021) [40] | PCS-12 (physical HRQoL) | 12 months | Blended care: baseline 30.0; estimated 12-month score approximately 35.8 | eUC: baseline 28.6; estimated 12-month score approximately 34.2; UC: baseline 29.6; estimated 12-month score approximately 35.4 | 251/252/126 | No significant between-group difference was reported. | No differential effect on physical HRQoL. |  |  |  |  |  |  |  |
|  | SF-12 MCS (income ≥C$50k) | 4 months | 40.78 → 48.29 | 43.70 → 47.73 | 42 / 39 | *β*=11.003, *P*=.006 | Exploratory subgroup benefit in higher-income participants only |  |  |  |  |  |  |  |
| Yu et al (2020) [37] | SF-12 Quality of Life | 12 months | 87.35 → 87.94 | 89.69 → 86.99 | 102 / 111 | Mean diff 1.18 (-3.18 to 5.54), *P*=.57 | No impact on quality of life |  |  |  |  |  |  |  |
| **Self-management, adherence, knowledge, and care-process outcomes** | | | | | | | |  |  |  |  |  |  |  |
| Chan et al (2022) [36] (JADE) | Self-management (≥2 activities) | 12 months | Increase 7.3% | Usual: +1.3%; Empowered: +2.7% | 796/795/802 | *P*<.05 for team vs usual | Improved self-management in team-based group |  |  |  |  |  |  |  |
| Clarke et al (2019) [63] | SMP-T2D Medication Adherence | 3 months | 87.50→86.34 | 87.20→92.04 | 232/241 | *β*=5.117, *P*=.004 | Control improved more |  |  |  |  |  |  |  |
| Gellis et al (2014) [59] (I-TEAM) | Problem-solving skills (SPSI-R) | 3 months | — → 14.6 | — → 8.4 | 46/48 | *P*<.001 | Improved coping skills |  |  |  |  |  |  |  |
| Hwang et al (2025) [51] | Digital health literacy (app use) | 8 weeks | 5.11 → 7.06 | 4.94 → 5.13 | 21/21 | Group×time χ²=6.33, *P*=.012 | Improved app use skills |  |  |  |  |  |  |  |
|  | Self-management behaviors | 8 weeks | No significant group×time | — | 21/21 | *P*>.05 | No difference |  |  |  |  |  |  |  |
|  | Medication adherence | 8 weeks | No significant group×time | — | 21/21 | *P*>.05 | No difference |  |  |  |  |  |  |  |
|  | Self-efficacy | 8 weeks | No significant group×time | — | 21/21 | *P*>.05 | No difference |  |  |  |  |  |  |  |
| Landucci et al (2025) [43] | Laptop vs Smart Display use | 8 months | Laptop: higher use, better ratings | — | 91 / 86 | All *P*<.01 for days of use & ratings | Laptop superior for engagement |  |  |  |  |  |  |  |
| Lear et al (2021) [64] | Self-management (heiQ) | 24 months | Improved in 4/8 domains | — | 116/113 | *P*<.05 for skill, self-monitoring, social, emotional | Improved self-management |  |  |  |  |  |  |  |
|  | Social support (MOS-SSS) | 24 months | Improved in 2/5 domains | — | 116/113 | *P*<.05 for emotional/info, overall | Improved social support |  |  |  |  |  |  |  |
| Liang et al (2021) [57] | Medication adherence | 6 months | 23.04→23.18 | 23.13→22.94 | 91/76 | GEE *β*=-0.082, *P*=.771 | No difference |  |  |  |  |  |  |  |
| Or et al (2020) [54] | Medication adherence | 24 weeks | 4.52 → 4.58 | 4.53 → 4.56 | 151/148 | Diff 0.03 (-0.05-0.12), *P*=.4 | No difference |  |  |  |  |  |  |  |
|  | Diabetes knowledge | 24 weeks | 78.5% → 84.4% | 79.1% → 85.4% | 151/148 | Diff -0.40 (-3.04-2.24), *P*=.77 | No difference |  |  |  |  |  |  |  |
|  | Hypertension knowledge | 24 weeks | 72.4% → 76.7% | 70.9% → 76.2% | 151/148 | Diff -1.01 (-3.40-1.38), *P*=.41 | No difference |  |  |  |  |  |  |  |
| Panagioti et al (2018) [66] | Patient Activation Measure (PAM) | 20 months | 62.88 | 61.92 | 326 / 577 | Adj diff 1.44 (-0.46-3.33), *P*=.13 | No effect |  |  |  |  |  |  |  |
|  | Self-care (SDSCA) | 20 months | 3.49 | 3.54 | 321/572 | Adj diff -0.04 (-0.19-0.11), *P*=.58 | No effect |  |  |  |  |  |  |  |
| Prabhakaran et al (2019) [32] | Medication adherence (7d) | 12 months | 81.1% / 82.4% | 57.9% / 68.9% | — | *P*<.001 | Higher adherence in IG |  |  |  |  |  |  |  |
| Stewart et al (2021) [60] (TIP) | heiQ (self-management) | 4 months | No significant difference (8 subscales) | — | 86 / 77 | All *β* non-sig (P>.05) | No effect on primary outcomes |  |  |  |  |  |  |  |
|  | Self-Efficacy for Chronic Disease | 4 months | 5.69 → 5.93 | 5.59 → 6.06 | 86/77 | *β*=-0.184 (-0.83-0.46), *P*=.58 | No effect |  |  |  |  |  |  |  |
| Yu et al (2020) [37] | Decisional Conflict Scale (DCS) | 12 months | 25.53 → 17.35 | 23.56 → 19.58 | 102 / 111 | Mean diff -3.49 (95% CI -7.4 to 0.42), *P*=.08 | Nonsignificant trend toward lower decisional conflict |  |  |  |  |  |  |  |
|  | Patient Assessment of Chronic Illness Care (PACIC) | 12 months | 2.82 → 3.68 | 3.16 → 3.22 | 102 / 111 | Mean diff 0.71 (0.38-1.04), *P*<.001 | Improved chronic illness care experience |  |  |  |  |  |  |  |
|  | Diabetes knowledge (%) | 12 months | 78.5 → 84.4 | 79.1 → 85.4 | 102 / 111 | Mean diff -0.40 (-3.04 to 2.24), *P*=.77 | No significant difference |  |  |  |  |  |  |  |
|  | Self-efficacy for chronic disease | 12 months | 7.31 → 7.49 | 6.98 → 7.24 | 102 / 111 | Mean diff -0.08 (-0.35 to 0.18), *P*=.55 | No significant difference |  |  |  |  |  |  |  |
| **Medication appropriateness and polypharmacy outcomes** | | | | | | | |  |  |  |  |  |  |  |
| Blum et al (2021) [39] (OPERAM) | Drug-drug interaction (2m) | 2 months | 462/832 (55.5%) | 521/893 (58.3%) | 832/893 | OR 0.87 (0.67-1.14), *P*=.31 | No difference |  |  |  |  |  |  |  |
|  | Drug overuse (2m) | 2 months | 348/832 (41.8%) | 376/893 (42.1%) | 832/893 | OR 0.99 (0.82-1.20), *P*=.91 | No difference |  |  |  |  |  |  |  |
| González-Ortega et al (2017) [65] | Number of medicines | 6 months | 8.7 → 8.6 (P=.628) | 8.9 → 9.2 (P=.063) | 71 / 79 | Mean diff 0.36 (95% CI -0.09 to 0.81), *P*=.113 | No significant difference |  |  |  |  |  |  |  |
| Jungo et al (2023) [38] (OPTICA) | MAI improvement (≥1 point) | 12 months | 68/160 (43%) | 67/163 (41%) | 160 / 163 | OR 1.05 (0.59-1.87), *P*=.87 | Inconclusive |  |  |  |  |  |  |  |
|  | AOU improvement (≥1 omission less) | 12 months | 24/160 (15%) | 28/163 (17%) | 160/163 | OR 0.90 (0.41-1.96), *P*=.79 | Inconclusive |  |  |  |  |  |  |  |
|  | MAI total score | 12 months | Mean 26 | Mean 25 | 160/163 | IRR 1.15 (0.74-1.79), *P*=.53 | No difference |  |  |  |  |  |  |  |
|  | Number of prescribing omissions | 12 months | 0.9 | 1.2 | 160/163 | IRR 0.83 (0.65-1.07), *P*=.15 | No difference |  |  |  |  |  |  |  |
|  | Number of medications | 12 months | 7.8 | 8.0 | 160/163 | MD 0.26 (-0.64-1.16), *P*=.58 | No difference |  |  |  |  |  |  |  |
| **Health care utilization, clinical events, and safety outcomes** | | | | | | | |  |  |  |  |  |  |  |
| Bernocchi et al (2018) [56] | Time to hospitalization/death | 4 months | Median 113.4 days | 104.7 days | 56/56 | Log-rank *P*=.048 | Significantly longer event-free survival |  |  |  |  |  |  |  |
| Blum et al (2021) [39] (OPERAM) | First drug-related hospital admission | 12 months | 211/963 (21.9%) | 234/1045 (22.4%) | 963 / 1045 | HR 0.95 (0.77-1.17), *P*=.62 | No significant reduction |  |  |  |  |  |  |  |
|  | First fall | 12 months | 237 (24.6%) | 263 (25.2%) | 963/1045 | HR 0.96 (0.79-1.15), *P*=.64 | No difference |  |  |  |  |  |  |  |
| Gellis et al (2014) [59] (I-TEAM) | ED visits (12m post) | 12 months | 0.6 | 1.4 | 46/48 | *P*=.03 | Fewer ED visits |  |  |  |  |  |  |  |
| Jungo et al (2023) [38] (OPTICA) | Number of falls | 12 months | 0.2 | 0.2 | 160/163 | IRR 0.90 (0.50-1.64), *P*=.74 | No difference |  |  |  |  |  |  |  |
| Lear et al (2021) [64] | All-cause hospitalizations | 24 months | 56 events | 81 events | 116 / 113 | RR 0.68 (0.43-1.10), *P*=.12 | 30.9% reduction, not significant |  |  |  |  |  |  |  |
|  | Composite hospitalization/death | 24 months | 37/116 (31.9%) | 51/113 (45.1%) | 116/113 | OR 0.57 (0.33-0.98), *P*=.04 | Lower composite outcome |  |  |  |  |  |  |  |
|  | Time to first hospitalization | 24 months | — | — | 116/113 | HR 0.62 (0.39-0.97), *P*=.04 | Lower risk |  |  |  |  |  |  |  |
|  | ED visits | 6 months | 12% | 26% | 100/100 | OR 0.388 (0.183-0.822), *P*=.013 | Fewer ED visits |  |  |  |  |  |  |  |
| Rollman et al (2021) [40] | Rehospitalizations (all-cause) | 12 months | Blended care: 61.2%; incidence rate/person-year 0.99 (95% CI 0.84 to 1.17) | eUC: 53.1%; incidence rate/person-year 1.10 (95% CI 0.94 to 1.29); UC: 58.2%; incidence rate/person-year 0.93 (95% CI 0.74 to 1.18) | 251/252/126 | No significant difference by treatment assignment was reported. | No differential reduction in rehospitalizations. |  |  |  |  |  |  |  |
|  | ED visits without hospitalization | 12 months | 16 (6.0%) | 6 (2.2%) | 267/267 | *P*=.027 | Increased ED visits without admission |  |  |  |  |  |  |  |
|  | Recurrent falls (hospitalization) | 12 months | 31/83 (37.3%) | 37/91 (40.7%) | 267/267 | *P*=.65 | No difference |  |  |  |  |  |  |  |
| Yao et al (2021) [31] (mAFA-II) | Composite outcome (stroke/TE, death, rehospitalization) | ~419/457 days | 49/833 (5.9%) | 195/1057 (18.4%) | 833 / 1057 | HR 0.37 (95% CI 0.26-0.53), *P*<.001 | Significant reduction in composite outcome |  |  |  |  |  |  |  |
|  | Rehospitalization | ~12 months | 33/833 (4.0%) | 116/1057 (11.0%) | 833/1057 | HR 0.42 (0.27-0.64), *P*<.001 | Reduced rehospitalization |  |  |  |  |  |  |  |
|  | Thromboembolism | ~12 months | 4/833 (0.5%) | 31/1057 (2.9%) | 833/1057 | HR 0.17 (0.05-0.51), *P*=.002 | Reduced TE events |  |  |  |  |  |  |  |
|  | Bleeding events | ~12 months | 22/833 (2.6%) | 47/1057 (4.4%) | 833/1057 | HR 0.63 (0.36-1.11), *P*=.11 | No significant difference |  |  |  |  |  |  |  |
| **Other reported outcomes** | | | | | | | |  |  |  |  |  |  |  |
| Baumeister et al (2021) [48] | Remission (SCID) | 6 months | 79 (76%) | 66 (63%) | 104/105 | OR 1.97 (1.05-3.68), *P*=.035 | Higher remission at 6 months |  |  |  |  |  |  |  |
|  | Work capacity (SPE) | 9 weeks | 1.70→1.77 | 1.76→1.62 | 104/105 | *β*=0.15 (-0.08 to 0.38), *P*=.197 | No difference |  |  |  |  |  |  |  |
| Bothelius et al (2024) [47] | Self-rated health | 9 weeks | 1.5 → 1.6 | 1.5 → 2.2 | 42/43 | Group×time *P*=.048, favoring IAR | IAR better |  |  |  |  |  |  |  |
| Clarke et al (2019) [63] | Work and Social Adjustment (WSAS) | 3 months | 13.64→12.24 | 12.23→10.82 | 232 / 241 | Group×Time *β*=0.196, *P*=.77 | Both improved, no group effect |  |  |  |  |  |  |  |
|  | SMP-T2D Physical Activity | 3 months | 53.44→55.98 | 48.26→52.44 | 232/241 | *β*=0.083, *P*=.98 | No difference |  |  |  |  |  |  |  |
| Gasslander et al (2022) [46] | CSQ Catastrophizing | 3 months | 13.73 → 11.90 | 12.39 → 12.75 | 95/92 | *d*=0.15, *P*<.001 | Reduced catastrophizing |  |  |  |  |  |  |  |
|  | CSQ Ignoring | 3 months | 10.63 → 11.09 | 9.89 → 9.43 | 95/92 | *d*=0.34, *P*=.042 | Improved coping |  |  |  |  |  |  |  |
| González-Ortega et al (2017) [65] | Charlson Comorbidity Index | 6 months | 3.7 → 3.8 (P=.161) | 3.7 → 3.8 (P=.011) | 71 / 79 | Mean diff -0.03 (95% CI -0.22 to 0.16), *P*=.794 | No significant difference |  |  |  |  |  |  |  |
|  | Norton Scale (pressure ulcer risk) | 6 months | 17.0 → 16.6 (P=.003) | 17.7 → 17.5 (P=.058) | 71 / 79 | Mean diff 0.16 (95% CI -0.14 to 0.46), *P*=.287 | No significant difference |  |  |  |  |  |  |  |
|  | Pfeiffer Test (cognitive) | 6 months | 1.8 → 1.9 (P=.201) | 1.4 → 1.4 (P=.902) | 71 / 79 | Mean diff -0.14 (95% CI -0.42 to 0.14), *P*=.328 | No significant difference |  |  |  |  |  |  |  |
| Mihevc et al (2025) [55] | BMI | 12 months | -0.2 (-0.5 to 0.1) | -0.1 (-0.4 to 0.2) | 55/62 | No significant difference | No effect |  |  |  |  |  |  |  |
|  | Behavioral risk factors | 12 months | No significant changes | — | 55/62 | All *P*>.05 | No lifestyle improvements |  |  |  |  |  |  |  |
| Monreal-Bartolomé et al (2025) [52] | Composite multimorbidity score | 3 months | Significant reduction | — | 93 / 90 | B = -0.34 (95% CI -0.64 to -0.04); Hedges g=0.39 | Improved composite score |  |  |  |  |  |  |  |
| Panagioti et al (2018) [66] | Cost-effectiveness (QALY) | 20 months | +0.019 QALY; +£151 cost | — | 504/802 | ICER £8,050/QALY; 70% probability cost-effective at £20k | Likely cost-effective |  |  |  |  |  |  |  |
| Stewart et al (2021) [60] (TIP) | Physical activity / healthy eating | 4 months | No significant difference | — | 86/77 | All *P*>.05 | No effect on health behaviors |  |  |  |  |  |  |  |
| Yao et al (2021) [31] (mAFA-II) | AF symptoms (reported) | ~12 months | 50/833 (6.0%) | 89/1057 (8.4%) | 833/1057 | HR 0.82 (0.56-1.20), *P*=.31 | No difference |  |  |  |  |  |  |  |

**Abbreviations:** 6MWT, 6-minute walk test; ACS, acute coronary syndrome; ACT, acceptance and commitment therapy; ADL, activities of daily living; ADS, Appraisal of Diabetes Scale; AF, atrial fibrillation; AQoL-6D, Assessment of Quality of Life–6 Dimension; AOU, Assessment of Underutilization; BATD, behavioral activation treatment for depression; BG, blood glucose; BIS, Bergen Insomnia Scale; BMI, body mass index; BP, blood pressure; BPI, Brief Pain Inventory; BPI-IS, Brief Pain Inventory–Interference Scale; CAT, COPD Assessment Test; CI, confidence interval; CPAQ-8, Chronic Pain Acceptance Questionnaire–8; CSQ, Coping Strategies Questionnaire; CVD, cardiovascular disease; DASS-21, 21-item Depression Anxiety Stress Scales; DBP, diastolic blood pressure; DCS, Decisional Conflict Scale; DDS, Diabetes Distress Scale; ED, emergency department; eUC, enhanced usual care; EQ-VAS, EuroQol visual analog scale; ES, effect size; GAD-7, 7-item Generalized Anxiety Disorder scale; HAM-D, Hamilton Depression Rating Scale; HADS, Hospital Anxiety and Depression Scale; HbA1c, glycated hemoglobin; heiQ, Health Education Impact Questionnaire; HF, heart failure; HR, hazard ratio; HRQoL, health-related quality of life; IAR, internet-based applied relaxation; ICER, incremental cost-effectiveness ratio; IG, intervention group; IRR, incidence rate ratio; JADE, Joint Asia Diabetes Evaluation; KCCQ-12, 12-item Kansas City Cardiomyopathy Questionnaire; LDL-C, low-density lipoprotein cholesterol; MADRS-S, Montgomery-Åsberg Depression Rating Scale–Self-rated; MAI, Medication Appropriateness Index; MCS-12, 12-item Mental Component Summary; MHI-5, 5-item Mental Health Inventory; MLHFQ, Minnesota Living with Heart Failure Questionnaire; MRC, Medical Research Council dyspnea scale; MOS-SSS, Medical Outcomes Study Social Support Survey; n.s., not significant; NRS, numeric rating scale; OFS, Openness to the Future Scale; OR, odds ratio; PACIC, Patient Assessment of Chronic Illness Care; PAM, Patient Activation Measure; PANAS, Positive and Negative Affect Schedule; PASE, Physical Activity Scale for the Elderly; PCS, Pain Catastrophizing Scale; PCS-12, 12-item Physical Component Summary; PHQ-8, 8-item Patient Health Questionnaire; PHQ-9, 9-item Patient Health Questionnaire; PROMIS, Patient-Reported Outcomes Measurement Information System; PSEQ, Pain Self-Efficacy Questionnaire; QALY, quality-adjusted life-year; QIDS, Quick Inventory of Depressive Symptomatology; QoL, quality of life; RAASi, renin-angiotensin-aldosterone system inhibitor; RR, risk ratio; SCID, Structured Clinical Interview for DSM; SDSCA, Summary of Diabetes Self-Care Activities; SF-12, 12-item Short Form Health Survey; SMP-T2D, Self-Management Profile for Type 2 Diabetes; SPE, Subjective Prognosis of Employment; TAU, treatment as usual; TC, total cholesterol; TG, triglycerides; TIP, Telemedicine IMPACT Plus; UC, usual care; UCLA, University of California, Los Angeles Loneliness Scale; VO2peak, peak oxygen uptake; WHOQOL, World Health Organization Quality of Life; WSAS, Work and Social Adjustment Scale.

**Note:** Rows are grouped by outcome domain for readability. Results are presented as reported in the original trial reports and may therefore include endpoint values, within-group changes from baseline, change scores, or model-based between-group estimates. This appendix summarizes additional outcomes, alternative time points, trial-reported analyses, and study-level findings that were not used as main pooled effect estimates in the primary meta-analyses. Rows corresponding to effect estimates used directly as main pooled inputs in Figure 2 were excluded, except where the source trial reported alternative time points, secondary measures, subgroup analyses, or nonpooled trial-reported analyses. Comparator values are shown when separately reported in the source trial; for multiarm trials, multiple comparator results are listed in the order reported by the source study. A right arrow (→) indicates change from baseline to follow-up within the same group; Δ indicates a reported change score. An em dash indicates that a separate comparator value was not reported or was not applicable. Approximate follow-up durations are shown with a tilde (~) when the source trial reported variable or mean follow-up rather than a fixed assessment time. Sample sizes are outcome specific when follow-up data were incomplete.
