# Supplementary material for: Effectiveness and Implementation of Digital Health Interventions on Physiological, Psychological, and Functional Outcomes in Adults With Multimorbidity: Systematic Review and Meta-Analysis of Randomized Controlled Trials
Source: J Med Internet Res. 2026 Jul 28;28:e90458. doi: 10.2196/90458 (PMC13412019; doi:10.2196/90458)
Supplement: Multimedia Appendix 7 [file jmir-v28-e90458-s007.docx]

**Exploratory subgroup analyses**

These subgroup analyses were exploratory and were conducted to investigate potential sources of between-study heterogeneity. For HbA1c, SBP, and DBP, follow-up duration and intervention orientation were completely overlapping across the available studies; accordingly, follow-up duration was prioritized as the formal subgroup variable, and intervention orientation was not interpreted as an independent source of heterogeneity. Subgroup findings should therefore be interpreted cautiously.

**Figure S1. Subgroup analysis of HbA1c by follow-up duration.**

**
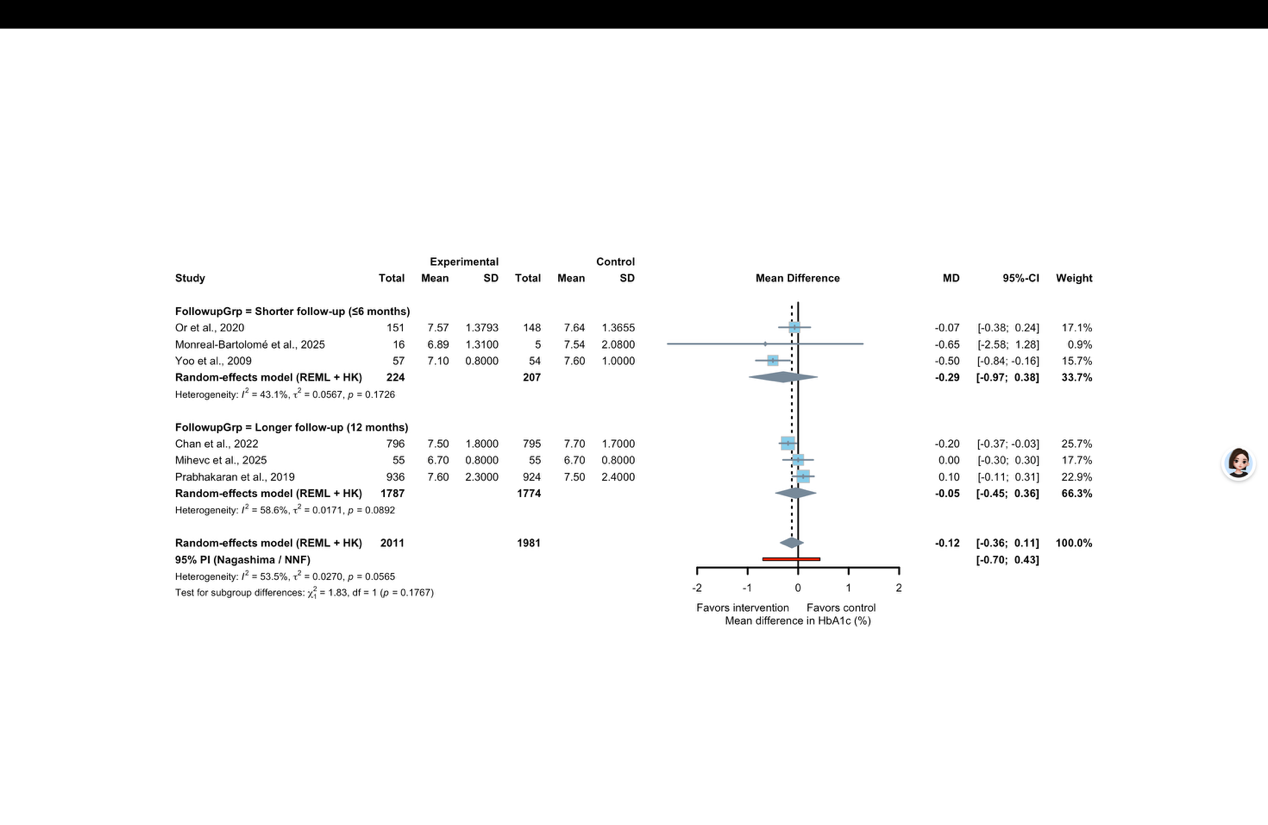
**

Negative mean differences indicate lower HbA1c values and favor the intervention. No statistically significant subgroup difference was observed (P=.18). The subgroup difference should be interpreted cautiously because follow-up duration and intervention orientation were completely overlapping across the available studies.

**Figure S2. Subgroup analysis of SBP by follow-up duration.**

**
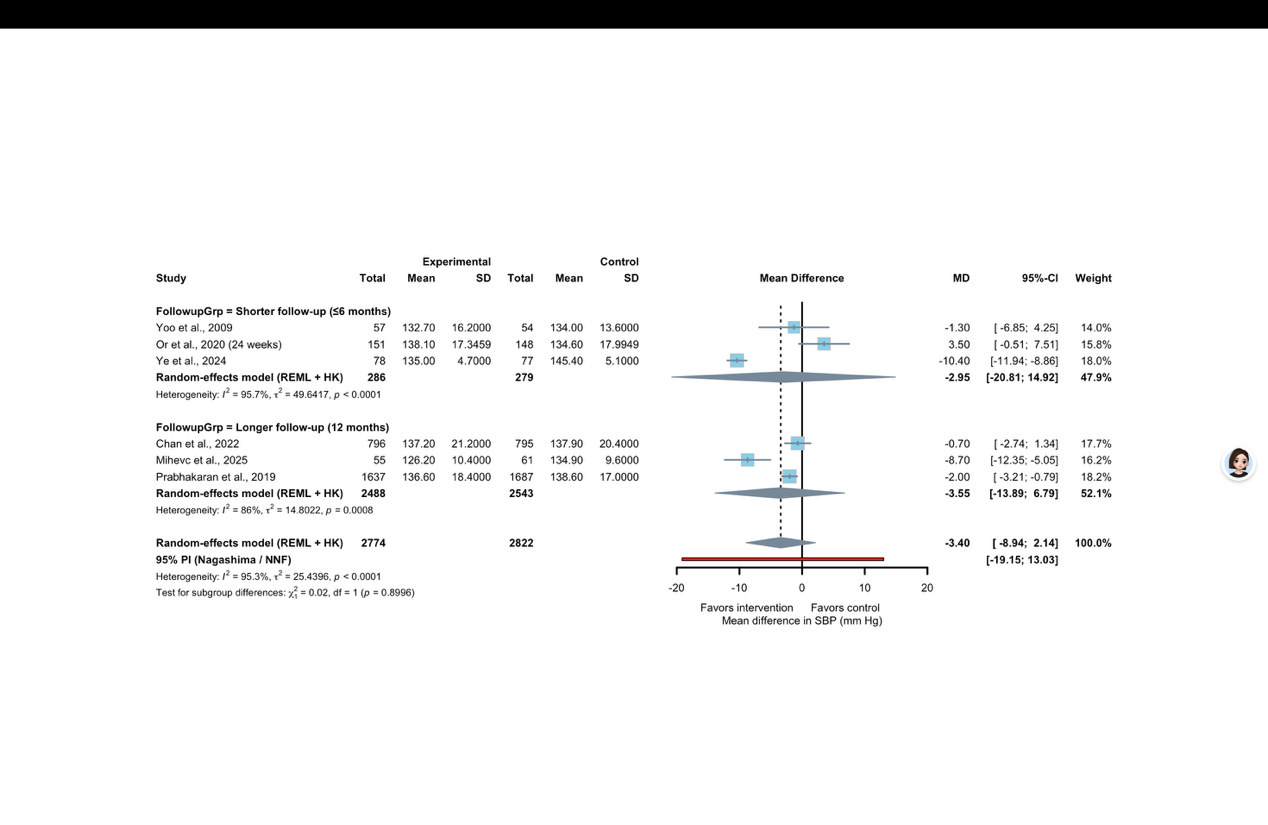
**

Negative mean differences indicate lower systolic blood pressure values and favor the intervention. No statistically significant subgroup difference was observed (P=.90). The subgroup difference should be interpreted cautiously because follow-up duration and intervention orientation were completely overlapping across the available studies.

**Figure S3. Subgroup analysis of DBP by follow-up duration.**


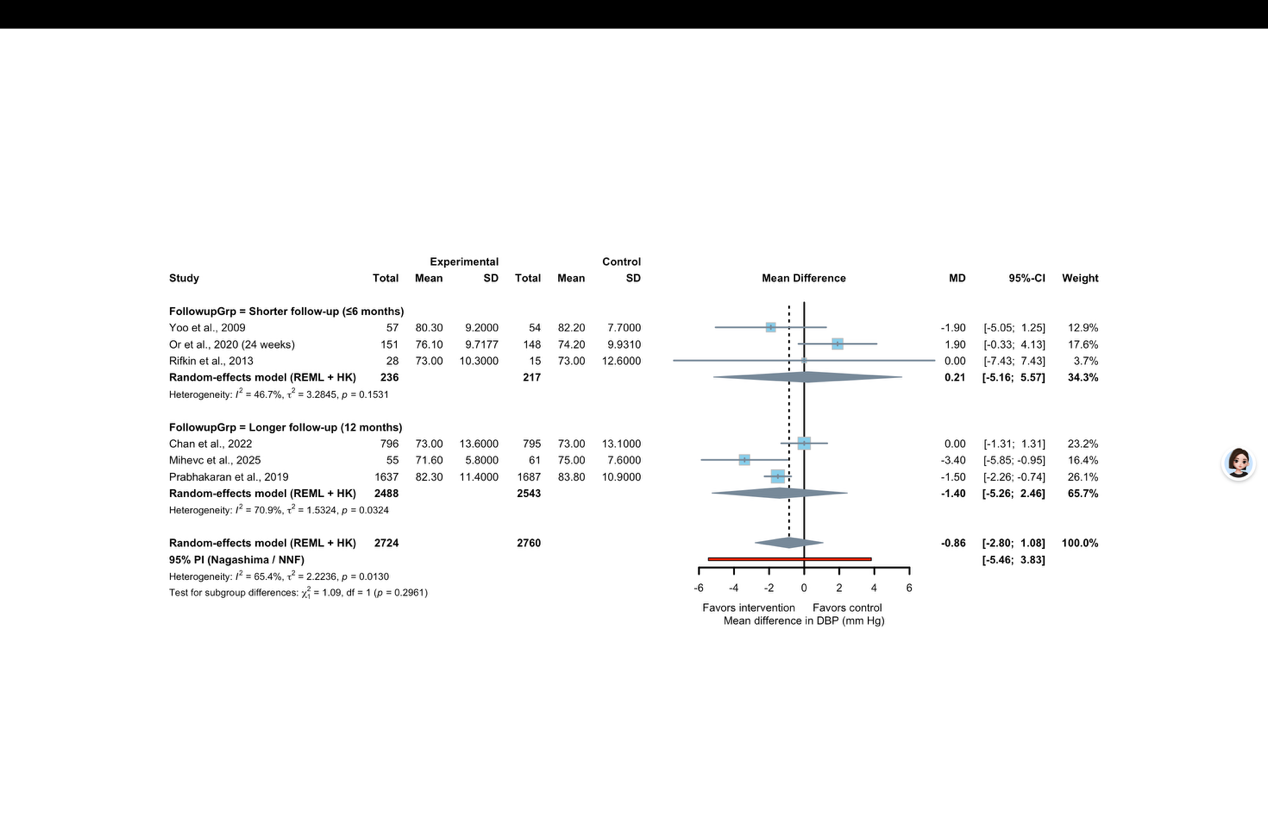


Negative mean differences indicate lower diastolic blood pressure values and favor the intervention. No statistically significant subgroup difference was observed (P=.30). The subgroup difference should be interpreted cautiously because follow-up duration and intervention orientation were completely overlapping across the available studies.
