# Supplementary material for: Effectiveness and Implementation of Digital Health Interventions on Physiological, Psychological, and Functional Outcomes in Adults With Multimorbidity: Systematic Review and Meta-Analysis of Randomized Controlled Trials
Source: J Med Internet Res. 2026 Jul 28;28:e90458. doi: 10.2196/90458 (PMC13412019; doi:10.2196/90458)
Supplement: Multimedia Appendix 8 [file jmir-v28-e90458-s008.docx]

**Leave-one-out sensitivity analyses**

Each leave-one-out analysis shows the pooled effect estimate after omission of 1 study, using the same random-effects model, REML estimation, Hartung-Knapp confidence intervals, and prediction interval approach as in the corresponding main analysis.

**Figure S1. Leave-one-out sensitivity analysis for HbA1c.**


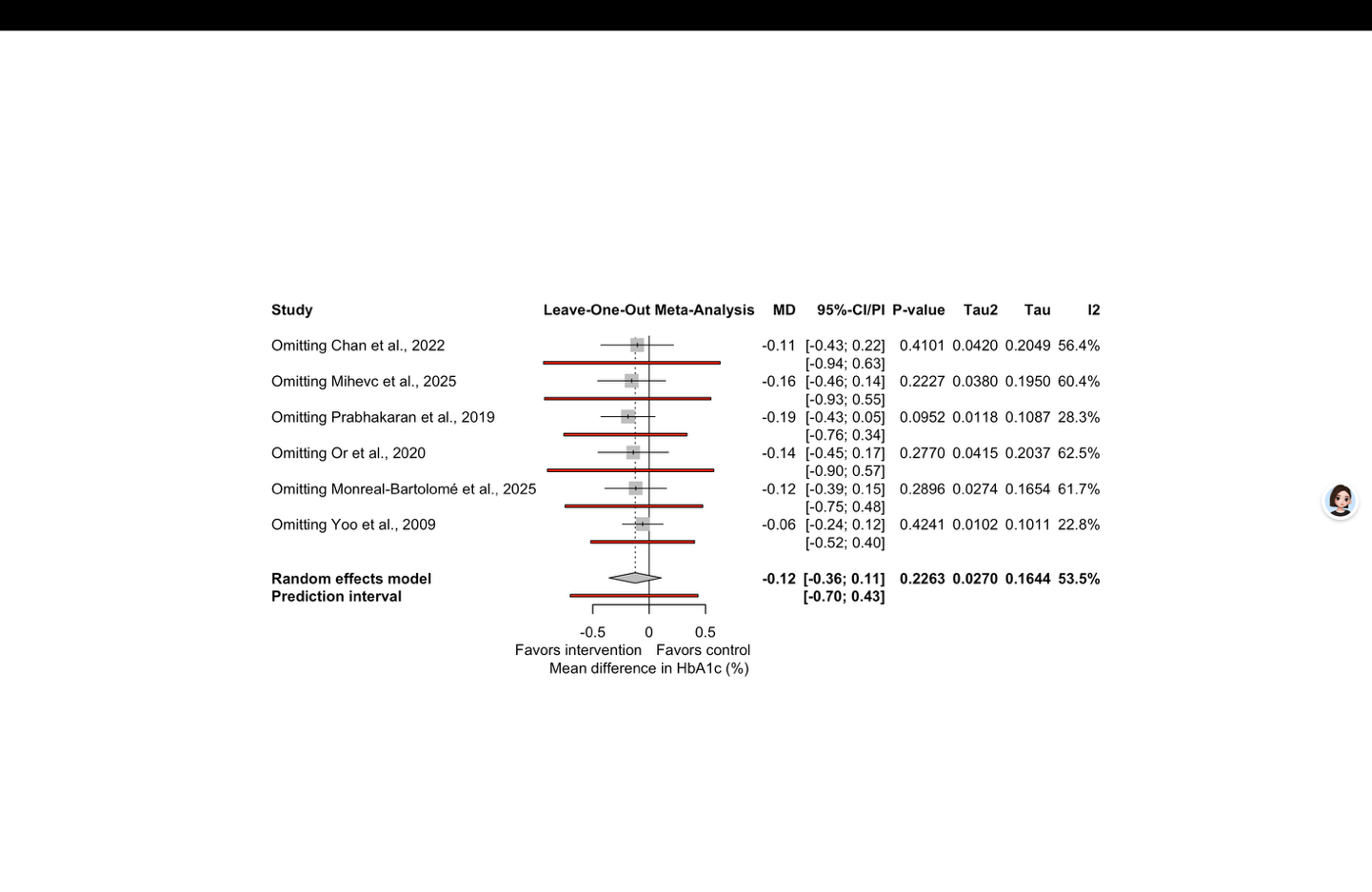


Each row shows the pooled mean difference after omission of 1 study. Negative mean differences indicate lower HbA1c values and favor the intervention.

**Figure S2. Leave-one-out sensitivity analysis for SBP.**


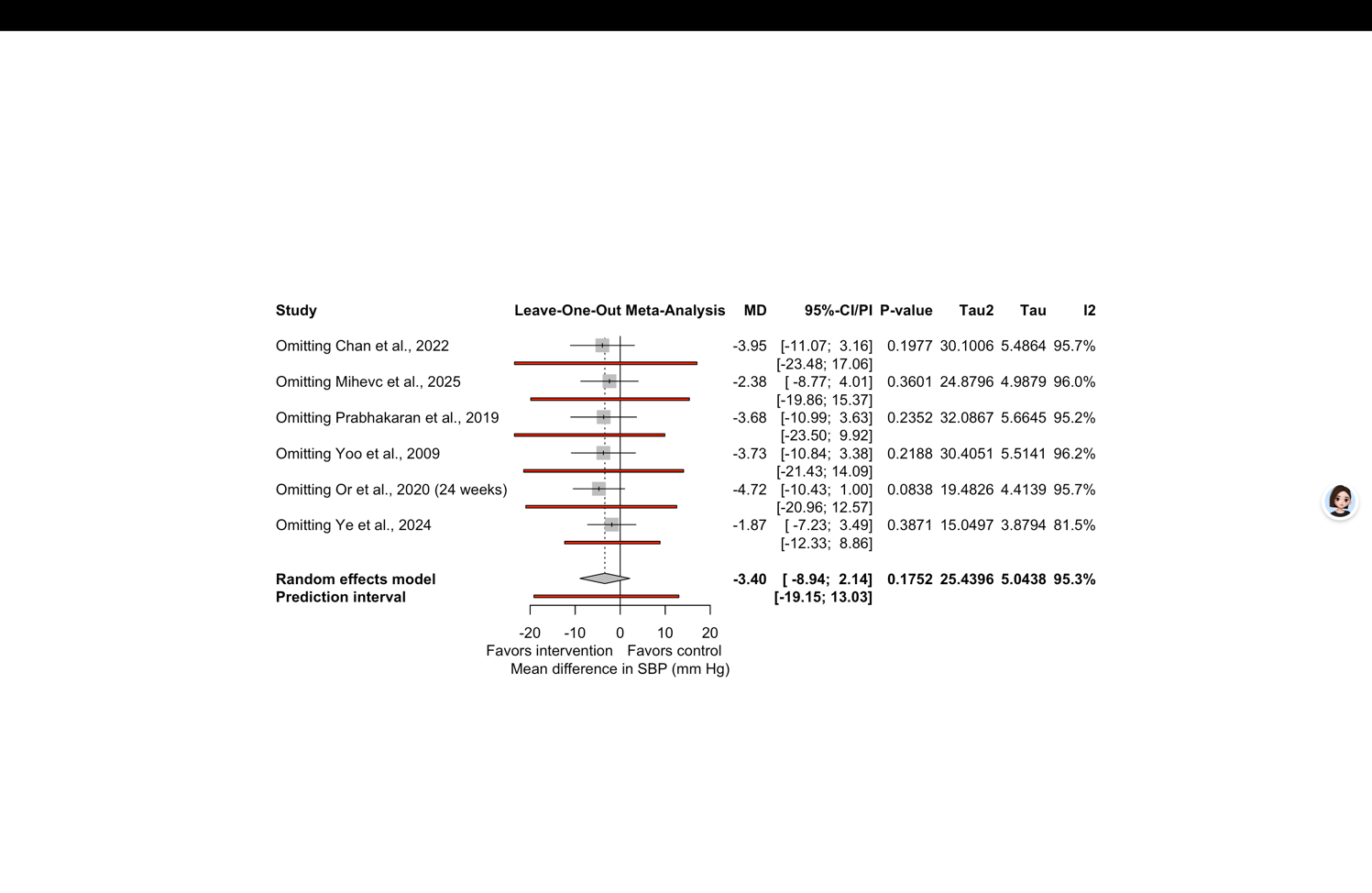


Each row shows the pooled mean difference after omission of 1 study. Negative mean differences indicate lower SBP values and favor the intervention.

**Figure S3. Leave-one-out sensitivity analysis for DBP.**


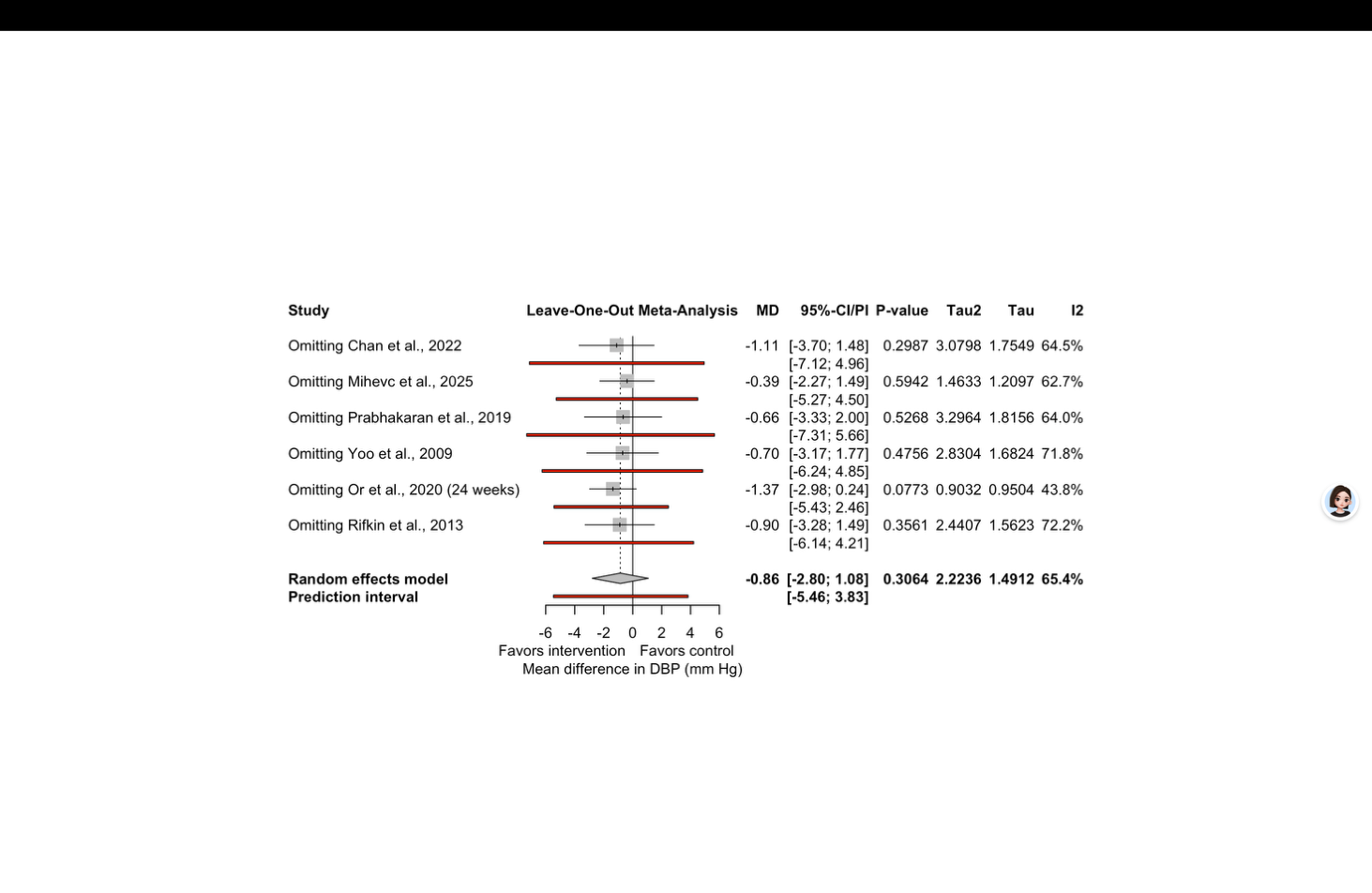


Each row shows the pooled mean difference after omission of 1 study. Negative mean differences indicate lower DBP values and favor the intervention.

**Figure S4. Leave-one-out sensitivity analysis for depression response.**


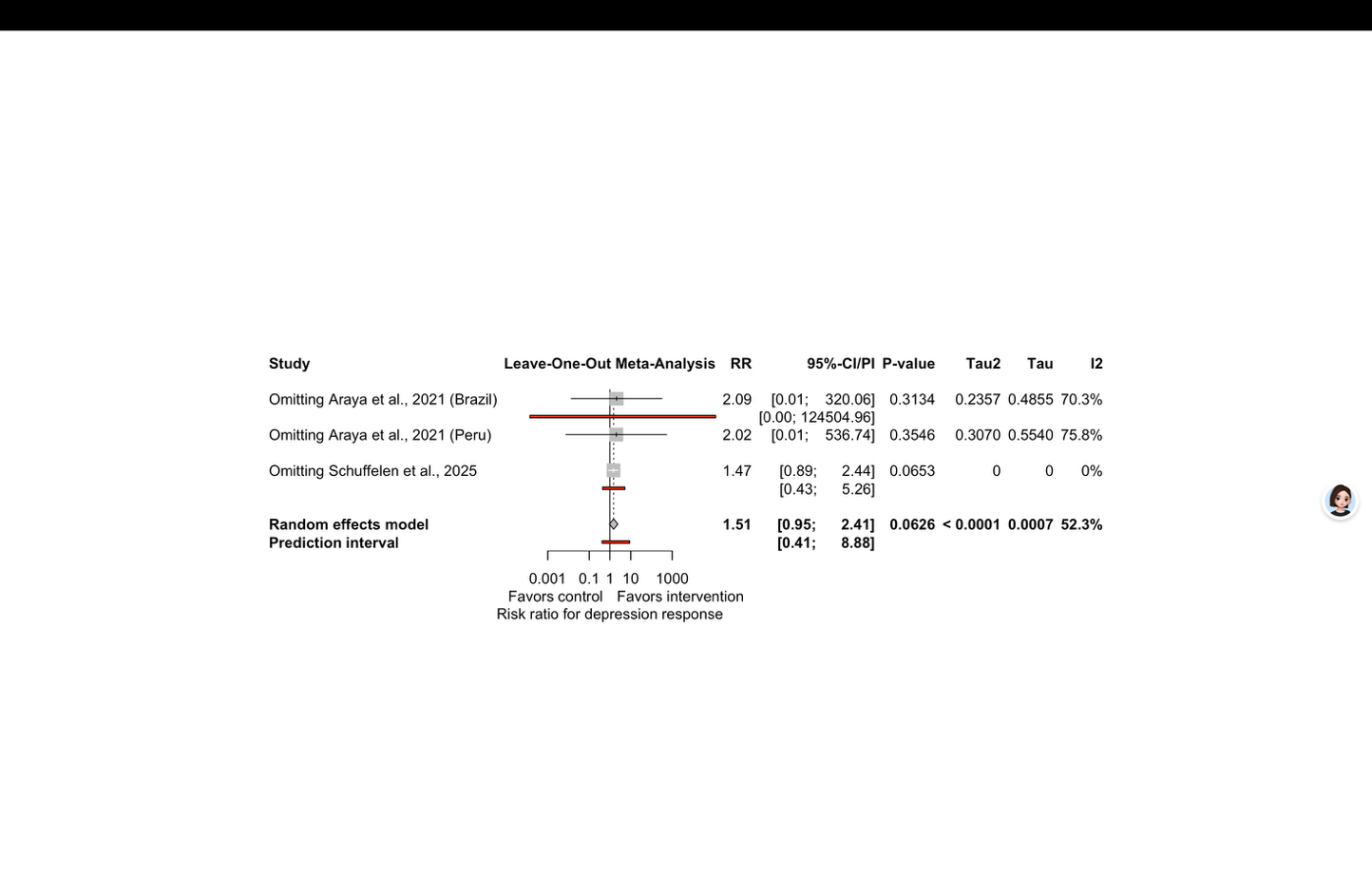


Each row shows the pooled risk ratio after omission of 1 study. Risk ratios above 1.0 indicate higher depression response and favor the intervention.

**Figure S5. Leave-one-out sensitivity analysis for depression severity.**


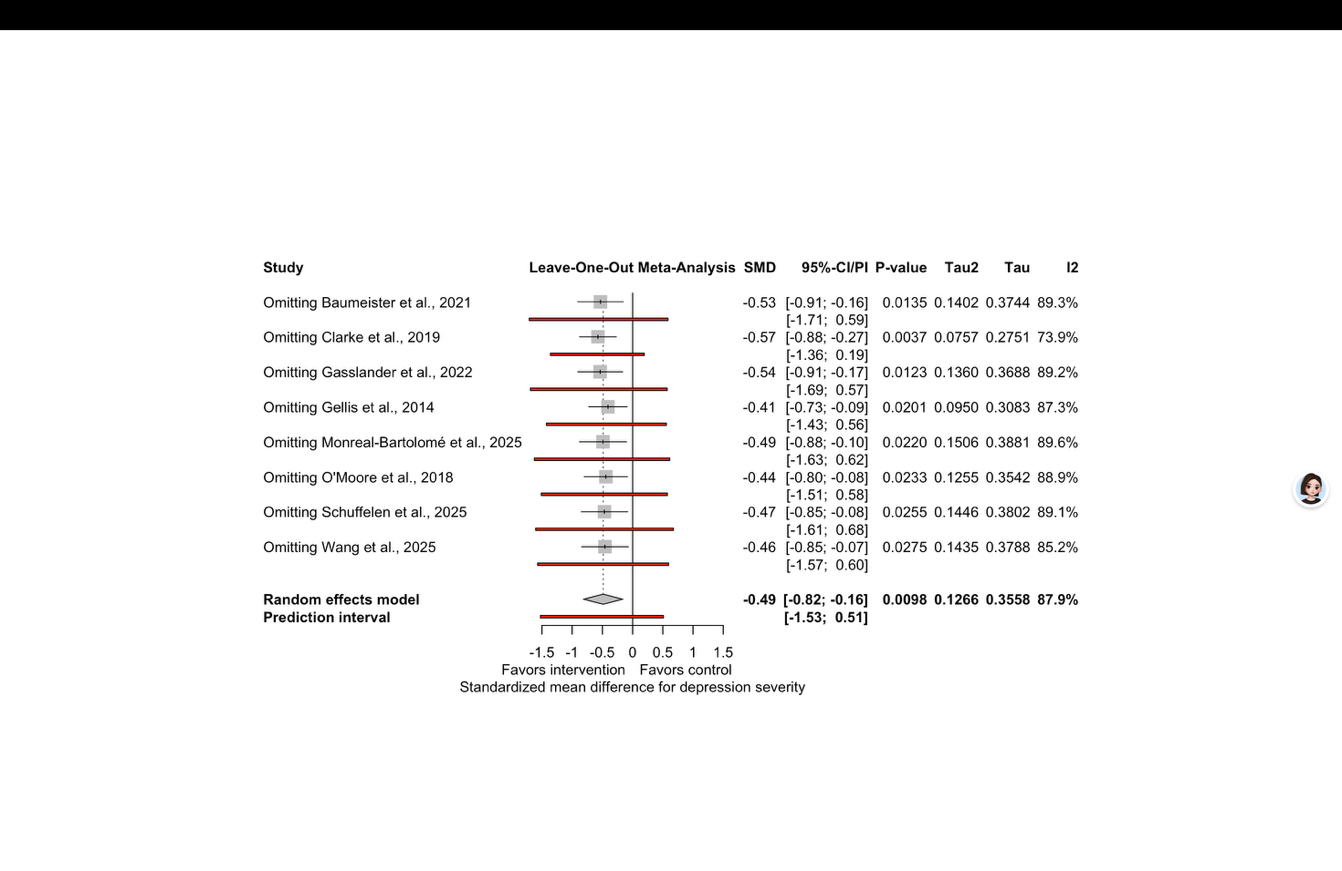


Each row shows the pooled standardized mean difference after omission of 1 study. Negative standardized mean differences indicate lower depression severity and favor the intervention.

**Figure S6. Leave-one-out sensitivity analysis for mortality.**


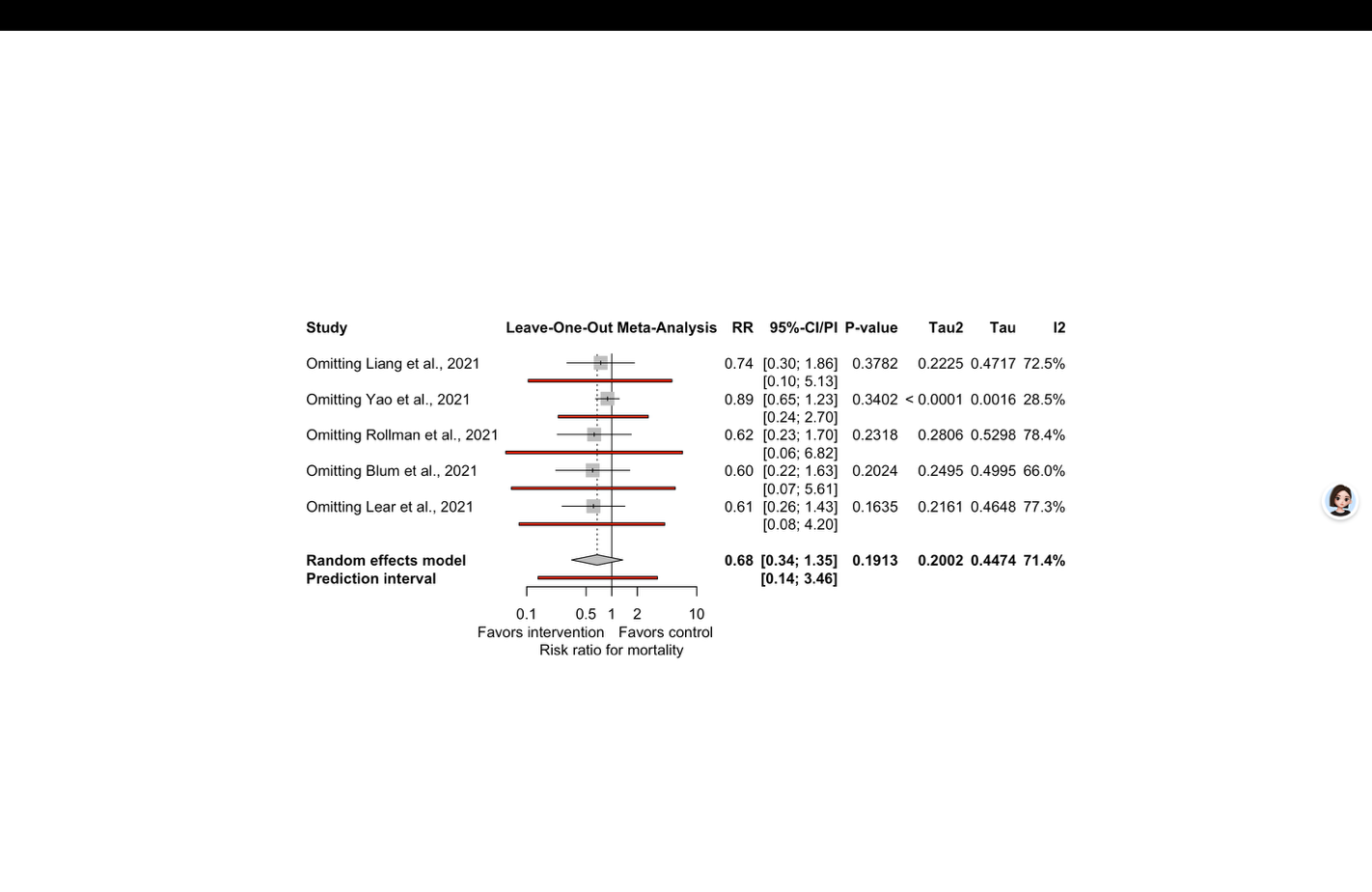


Each row shows the pooled risk ratio after omission of 1 study. Risk ratios below 1.0 indicate lower mortality and favor the intervention.

**Figure S7. Leave-one-out sensitivity analysis for hospitalization/readmission.**


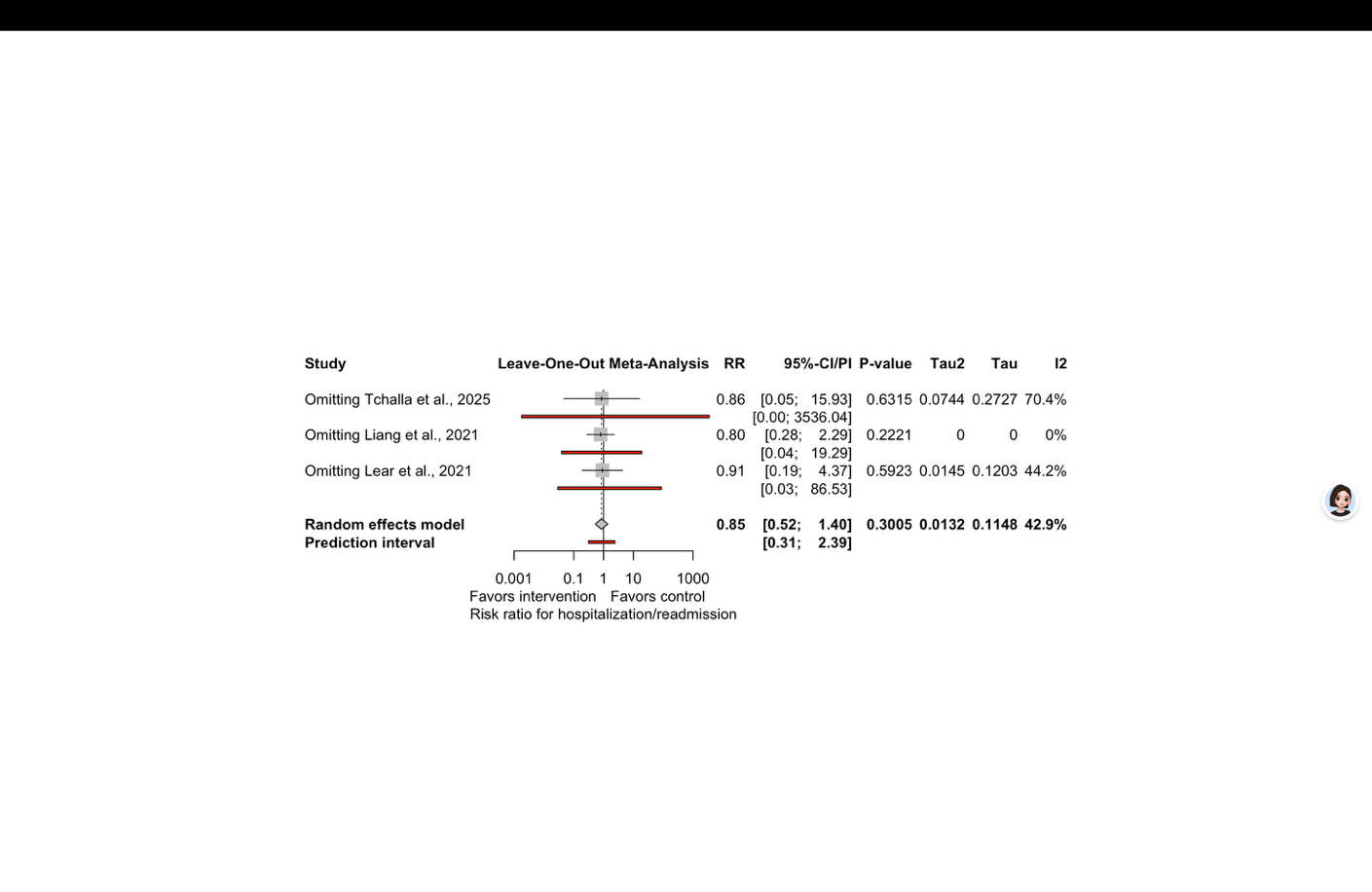


Each row shows the pooled risk ratio after omission of 1 study. Risk ratios below 1.0 indicate fewer hospitalization/readmission events and favor the intervention.

**Figure S8. Leave-one-out sensitivity analysis for mental HRQoL.**


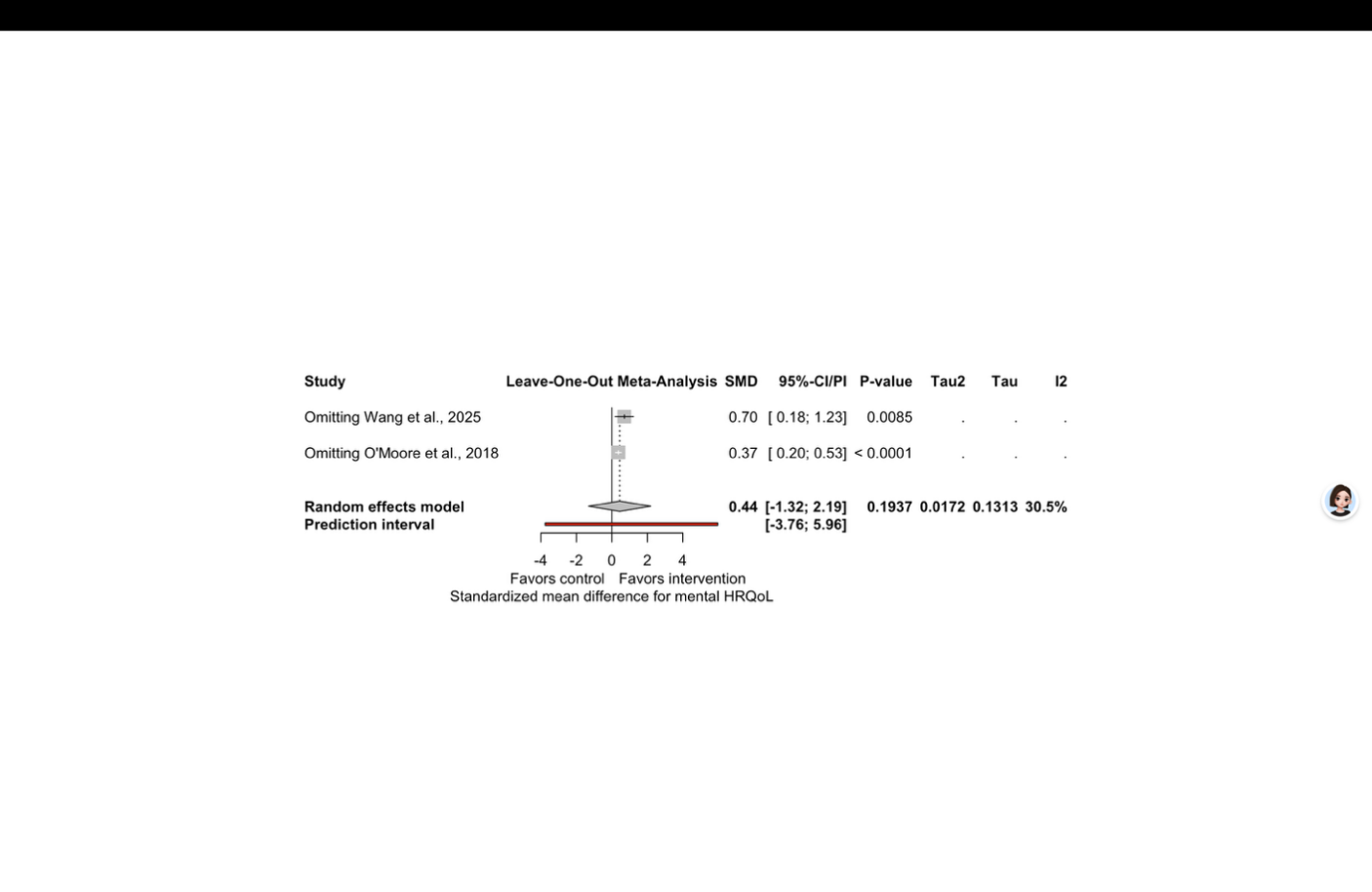


Each row shows the pooled standardized mean difference after omission of 1 study. Positive standardized mean differences indicate better mental HRQoL and favor the intervention.

**Figure S9. Leave-one-out sensitivity analysis for physical HRQoL.**

**
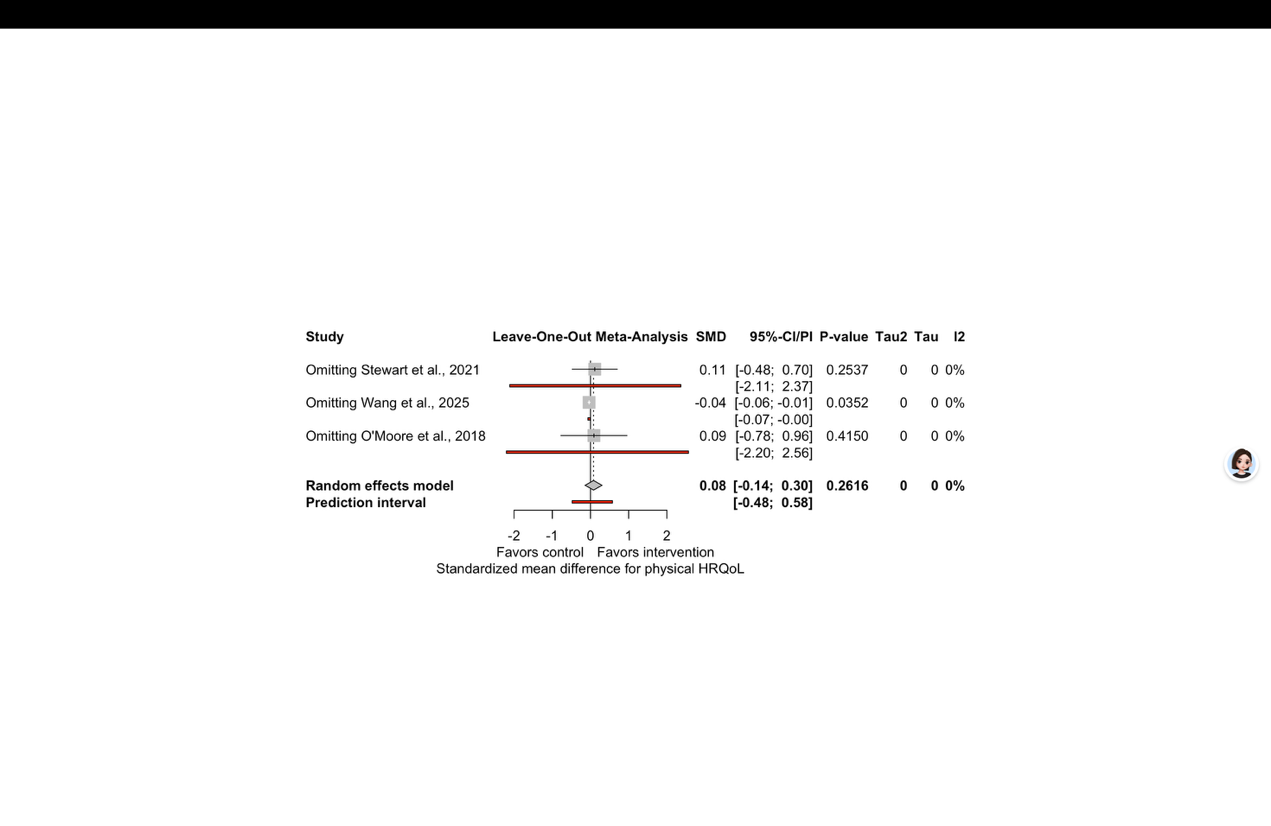
**

Each row shows the pooled standardized mean difference after omission of 1 study. Positive standardized mean differences indicate better physical HRQoL and favor the intervention.

**Figure S10. Leave-one-out sensitivity analysis for pain-related functional impact/disability.**


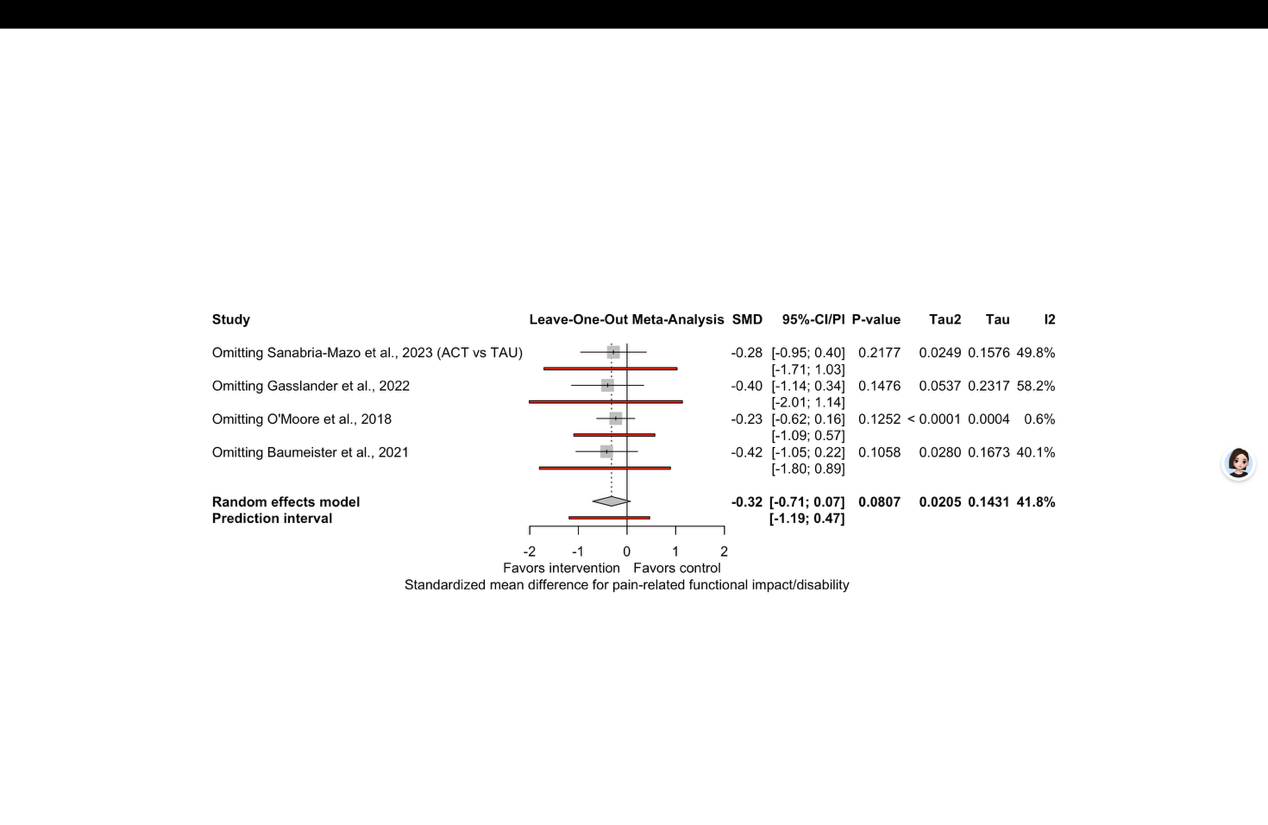


Each row shows the pooled standardized mean difference after omission of 1 study. Negative standardized mean differences indicate lower pain-related functional impact/disability and favor the intervention.
