# Supplementary material for: Effectiveness and Implementation of Digital Health Interventions on Physiological, Psychological, and Functional Outcomes in Adults With Multimorbidity: Systematic Review and Meta-Analysis of Randomized Controlled Trials
Source: J Med Internet Res. 2026 Jul 28;28:e90458. doi: 10.2196/90458 (PMC13412019; doi:10.2196/90458)
Supplement: Multimedia Appendix 9 [file jmir-v28-e90458-s009.docx]

**Table S1.** Study-level implementation data for mode of delivery, reach, adherence, and feasibility

| **Study (author, year)** | **Mode of delivery** | **Reach information** | **Adherence indicator** | **Feasibility summary** |
| --- | --- | --- | --- | --- |
| **González-Ortega et al (2017) [65]** | Telephone coaching (family physician, twice monthly) | 22,140 → 4,622 CRG → 1,007 sample → 453 CCP → 297 invited → 161 enrolled (54%) | IG: mean 8/11 calls (73%); 5-8 min/call | Retention: 93% at 6mo; 9 died, 2 lost. No major side effects. |
| **Chan et al (2022) [36] (JADE)** | Web portal + Nurse telephone + Face-to-face team | 2,421 screened → 2,393 randomized; 13 centers, 8 Asian regions | Team: 5.7 visits/calls; Empowered: 2.8 calls. Adherence: 75.9% team, 80.2% empowered, 99.5% usual care | 12-month retention: 85.3%-87.4%. High return rate. |
| **Baumeister et al (2021) [48]** | Internet-based IMI (guided, 6 core + 3 optional modules) | 9,213 in-clinic + 18,409 letters → 210 randomized | 87% intro; 55% all 6 core; eCoach 101 min/completer; session 54 min | Retention: 89% at 9wk, 85% at 6mo. CSQ-8 mean 24.1; 87% would recommend. |
| **Liang et al (2021) [57]** | Tele-homecare (24/7 call center, wireless devices) | 200 enrolled (100 IG, 100 CG); LACE ≥7; mean age 80.7, 2.72 chronic conditions | 8,111 alerts (93.2% vital signs); 121 extra home visits | Retention: 83.5% overall (IG 91%, CG 76%); attrition mainly due to death (8 IG, 19 CG). No device-related AEs. |
| **Wakefield et al (2011) [41]** | Home telehealth (device + nurse care mgmt; high/low intensity) | 2,756 → 1,272 contacted → 302 randomized | High: 127/182 days (70%); Low: 125/182 days (69%) | Retention: 85% at 6mo, 81% at 12mo; dropout 9%. |
| **Tchalla et al (2025) [58]** | Home telemonitoring (e-COBAHLT: biometric sensors, geriatrician) | 534 randomized (267 IG, 267 CG); mean age 80.3; post-discharge | 19/267 (7.1%) did not complete; no telehealth side effects | Retention: 92.1% at 12mo (492/534). Feasible and safe. |
| **Yao et al (2021) [31] (mAFA-II)** | mHealth app (mAFA) + integrated ABC pathway | 1,890 with multimorbidity analyzed (833 IG, 1,057 UC); ancillary analysis of mAFA-II trial; mean follow-up 419d IG, 457d UC | Not reported in this ancillary analysis | Not reported in this ancillary analysis |
| **Monreal-Bartolomé et al (2025) [52]** | Blended (2 face-to-face + 6 web modules) | 295 → 183 randomized (93 IG, 90 CG); mean age 51.4 | Median 4/7 modules; 95% first F2F, 63% second | 3-month retention: 49% IG vs 55% CG. High attrition in older/diabetes patients. |
| **Rollman et al (2021) [40]** | Blended collaborative care (telephone nurse + physician) | 7,866 screened → 629 depressed + 127 non-depressed → 756 total | Median 3 care manager contacts/month in both blended and eUC | Follow-up rates similar across arms; 98 deaths (none suicide). |
| **Gustafson et al (2024) [62]** | Web-based ElderTree (discussion, health tracking, clinician report) | 346 randomized (176 IG, 168 CG); mean age 74.8; ≥3 conditions | 94.3% first month; 76.1% at 12mo; 48.95 days first 6mo. 92% weekly tracker. | Retention: 92.4% analyzed; 89.8% completed 12mo. High sustained engagement. |
| **Bernocchi et al (2018) [56]** | Telerehabilitation (remote + weekly nurse/PT calls) | 112 randomized (56 IG, 56 CG); mean age 70; COPD+CHF | 86% completed 4mo; 93% exercised (19% 2.3/wk, 65% 4/wk, 16% 6/wk) | IG lost 20% vs CG 37.5% (P=.036). Satisfaction 22.3/24. No major AEs. |
| **Mihevc et al (2025) [55]** | mHealth telemonitoring (app + BP/BG + teleconsultation) | 128 enrolled (65+ yrs, HTN + T2D); 117 completed 12mo | Mean 2.7 BP/wk, 1.2 BG/wk (exceeded expected) | Retention: 93.8% at 6mo, 91.4% at 12mo. Some tech challenges in elderly. |
| **Yu et al (2020) [37]** | Web-based PtDA (MyDiabetesPlan) in primary care | 10 FHT clusters; 213 enrolled (111 IG, 102 CG) | 52/102 (51%) IG completed ≥2 plans | 29% withdrew/lost. Higher attrition in non-English speakers, lower education. |
| **Jungo et al (2023) [38] (OPTICA)** | eCDSS (STRIPA) + GP review + shared decision | 43 GP clusters; 323 patients (160 IG, 163 CG); median age 77 | 5.4 recs/patient; 1.0 implemented/patient; 58.5% ≥1 rec implemented | EMR export issues caused missing data (~13-18%). |
| **Blum et al (2021) [39] (OPERAM)** | Inpatient STRIPA + GP report | 2,008 randomized; 110 clusters; 4 European countries | 86.1% ≥1 STOPP/START rec (mean 2.75); 62.2% ≥1 implemented at 2mo; 97 min/patient | Retention: 99.5% at 12mo. No detriment; intervention safe. |
| **Clarke et al (2019) [63]** | Web-based CBT (myCompass, self-guided) | 6,145 visits → 3,223 consented → 780 randomized | Mean 6 logins; 0.71 modules started, 0.29 completed. 54.7% found easy. | Post retention: 59.2% IG vs 63.3% CG. Higher attrition with severe symptoms. |
| **Lear et al (2021) [64]** | Internet CDM (symptom reporting + nurse phone + primary care) | 3,438 letters → 456 screened → 230 randomized; mean age 70.5 | Median 2.5 logins/wk; 84.5% ≥1/wk; 96.6% completed; 32,095 alerts | Retention: 94% completed; 1 withdrew. High engagement with primary care integration. |
| **Prabhakaran et al (2019) [32] (mWellcare)** | mHealth EDS (tablet app + nurse + SMS) | 4,270 → 3,698 randomized; 40 CHCs India | 86.9% ≥1 follow-up; 47.6% ≥4 visits; median 3.0. Physician acceptance 68-69% | Retention: 89.9% at 12mo. Main barrier: drug unavailability. |
| **Yoo et al (2009) [53]** | Cellular + Internet (UCDC: auto transmission + SMS feedback) | 123 enrolled (57 IG, 54 CG); mean age ~58 | Compliance: BG 92.2%, BP 86.0%, weight 87.4%. Dropout: 8.1% IG vs 10% CG | Retention: 91.9% IG, 90% CG at 12wk. High satisfaction. |
| **Gellis et al (2014) [59] (I-TEAM)** | Integrated telehealth (daily monitoring + 8 PST sessions by nurse) | 302 → 115 randomized; mean age 79; homebound CHF/COPD+depression | IG: 51/57 (89%) completed. 91% continued monitoring in maintenance | 48 IG, 46 CG completed. High fidelity after 6wk supervision. |
| **Bothelius et al (2024) [47]** | Internet CBT-I (8 modules, therapist async messaging) | 1,359 → 85 randomized; mean age 48; pain+insomnia | Mean 2.0/8 (ICBT-I), 2.4/8 (IAR). 31% no modules; 4.8% all modules | Low engagement linked to low baseline QoL and activity engagement. |
| **Sanabria-Mazo et al (2023) [42]** | Videoconference group (ACT/BATD; 8 weekly 1.5h) | 768 → 234 randomized (78/78/78); CLBP+depression | ACT: 4.65 sessions; BATD: 4.42. 21.8% ACT, 12.8% BATD zero sessions. Wave 3 dropout 55.3% | Post: 66.6% ACT, 53.8% BATD, 82% TAU. 12mo: 56.4%/50%/67.9%. |
| **Or et al (2020) [54]** | Tablet TSN (Bluetooth BP/BG + education + audio reminders) | 299 randomized (151 IG, 148 CG); T2D+HTN | IG: 4.67 BG/wk, 5.26 BP/wk; CG: 4.47 BG/wk, 4.07 BP/wk (P>.05) | Low loss to follow-up. No study-related AEs. |
| **Gasslander et al (2022) [46]** | Tailored ICBT (6-13 modules; clinician-guided) | 933 → 187 randomized; mean age 45.9; pain+distress | Assigned 10.2 modules; completed 5.1 (50.4%); 23.2% all modules. Rated 2.45/4 helpful | 74.9% post retention. Completers had higher baseline pain acceptance, self-efficacy, QoL. |
| **Hwang et al (2025) [51]** | mHealth-based digital health coaching self-management program (DHCSMP-MCC) + individual telephone coaching + 3 in-person group sessions (weeks 1, 4, and 8) | 107 assessed → 49 randomized (25 IG, 24 CG); mean age ~72; living alone, ≥2 chronic conditions | App-based adherence data were used to calculate goal attainment; module completion was not reported. | Overall attrition was 14.3% (16% in the intervention group and 12.5% in the control group); 42 participants completed the 8-week follow-up. |
| **Landucci et al (2025) [43]** | Smart display (Google Nest Hub Max) vs Laptop (ElderTree) | 269 randomized (91 control, 92 laptop, 86 smart display); mean age 69.8; chronic pain + ≥3 conditions | Laptop: mean 28.5 days use in months 1-4 vs Smart display: 19.0 days (P<.001); 39% laptop vs 36% smart display attended ≥1 meetup | Laptop use was associated with more favorable ratings; smart display engagement was hindered by voice dictation errors and difficulty with text-heavy functions (eg, discussion boards and journaling). |
| **Chiang et al (2020) [61]** | Telemedicine-based (heart rate sensing clothes + mobile app + LINE communication) + weekly follow-up | 60 approached → 50 randomized (25 IG, 25 CG); patients with cardiometabolic multimorbidity (≥2 conditions) | 24/25 intervention participants (96%) completed all 36 exercise sessions | 47/50 participants (94%) completed the 12-week study; no adverse events reported |
| **Stewart et al (2021) [60] (TIP)** | Multi-provider case conference (F2F/video) + nurse coordination | 9 sites; 163 randomized (86 IG, 77 CG); ≥3 conditions | ≥3h nurse follow-up or ≥6 providers associated with poorer outcomes | Fidelity variations may have compromised impact. Resource-intensive. |
| **Panagioti et al (2018) [66]** | Telephone health coaching (6 calls + social prescribing + mood support) | 12,989 → 4,377 cohort → 1,306 eligible → 504 selected IG | 41% consented; 38% ≥1 call; 85% of those received 4+ calls. Consent: younger age (OR 1.08), higher education (OR 4.07) | 59% of selected did not take up. TWiCs design showed reach limitations. |
| **Wang et al (2025) [33] (CIC-PDD)** | Community integrated care (specialist + case manager + health communicator) | 8 CHCs; 4,735 DM → 3,759 invited → 630 randomized | Follow-up per care plan; case manager bridged specialist-primary care | Retention: 95.4% at 6mo, 92.9% at 12mo. 12 deaths, none related. |
| **Ye et al (2024) [34]** | WeChat telehealth education (daily posts, Q&A, peer sharing) | 174 → 160 randomized (80 IG, 80 CG); T2D+HTN | IG: 78/80 (97.5%); CG: 77/80 (96.3%) completed | Retention: 96.9% (155/160). WeChat feasible and acceptable. |
| **Araya et al (2021) [35] (CONEMO)** | Smartphone app (18-session BA) + nurse support | São Paulo: 10,688 → 880; Lima: 5,785 → 432 | São Paulo: 18% zero sessions, 65% ≥9, 45% all 18. Lima: 2% zero, 92% ≥9, 78% all 18 | São Paulo 91.7%, Lima 98.6% follow-up. Lima: dedicated nurses enhanced adherence. |
| **O’Moore et al (2018) [50]** | iCBT (Sadness Program; 6 lessons + email support) | 301 → 69 randomized (44 iCBT, 25 TAU); knee OA+MDD | 37/44 (84%) completed all 6 lessons. 95% satisfied, 93% would recommend | 42/44 iCBT, 23/25 TAU completed post. Minimal attrition. |
| **Rifkin et al (2013) [45]** | Bluetooth BP + Home hub (cellular; weekly pharmacist/physician review) | 336 → 47 enrolled → 43 analyzed; CKD+HTN | Median 29 readings/mo; 78% ≥4/mo throughout 6mo. CG: 20% brought logs | 43/47 completed. 96% would continue. Minor tech issues (hub 14%, cuff 29%). |
| **Schuffelen et al (2025) [49]** | Digital CBT-I (somnio; 10 modules; avatar + push notifications) | 533 → 140 randomized (70 IG, 70 WLC); depression+insomnia | 67.1% ≥5/10 modules; 37.1% all 10. 48.6% satisfied; 38.6% expectations fulfilled | Dropout: 16.4% at 12wk, 21.4% at 24wk. No AEs. |
| **Hsu et al (2021) [44]** | Telemedicine dietitian + Home elastic band exercise | 113 → 66 randomized (22 D, 22 E, 22 D+E); obesity + knee OA | Adherence: D=83%, E=90%, D+E=87% | 63/66 (95.5%) completed 12wk. No AEs. Telemedicine follow-up feasible. |

**Abbreviations:** ACT, acceptance and commitment therapy; AE, adverse event; BA, behavioral activation; BATD, behavioral activation therapy for depression; BG, blood glucose; BP, blood pressure; CBT, cognitive behavioral therapy; CBT-I, cognitive behavioral therapy for insomnia; CCP, complex chronic patient; CDM, chronic disease management; CDSS, clinical decision support system; CG, control group; CHC, community health center; CHF, congestive heart failure; CKD, chronic kidney disease; CLBP, chronic low back pain; COPD, chronic obstructive pulmonary disease; CRG, clinical risk group; D, diet control group; DHCSMP-MCC, digital health coaching self-management program for multiple chronic conditions; E, exercise group; eCDSS, electronic clinical decision support system; EDS, electronic decision support; eUC, enhanced usual care; F2F, face-to-face; FHT, family health team; GP, general practitioner; HTN, hypertension; IAR, internet-based applied relaxation; ICBT, internet-delivered cognitive behavioral therapy; IG, intervention group; IMI, internet- and mobile-based intervention; I-TEAM, Integrated Telehealth Education and Activation of Mood; JADE, Joint Asia Diabetes Evaluation; LACE, length of stay, acuity of admission, comorbidity of the patient, and emergency department use; mAFA, mobile Atrial Fibrillation Application; MDD, major depressive disorder; mo, month; OA, osteoarthritis; OPTICA, Optimising PharmacoTherapy In the multimorbid elderly in primary CAre; OPERAM, OPtimising thERapy to prevent Avoidable hospital admissions in Multimorbid older people; PT, physiotherapist; PtDA, patient decision aid; rec, recommendation; SMS, short message service; STRIPA, Systematic Tool to Reduce Inappropriate Prescribing Assistant; T2D, type 2 diabetes; TAU, treatment as usual; TIP, Telemedicine IMPACT Plus; TSN, technological surrogate nursing; TWiCs, Trials within Cohorts; UC, usual care; UCDC, Ubiquitous Chronic Disease Care; WLC, waiting-list control; wk, week.

**Note:** “Reach information” summarizes the most comparable study-reported recruitment or participation information available. Because explicit invitation or screening denominators were often not reported, some entries reflect randomized sample size, completion, or follow-up rather than true reach. “Adherence indicator” summarizes study-reported intervention use, adherence, engagement, retention, or implementation continuity. Feasibility summaries are narrative interpretations based on study-reported findings and should not be interpreted as directly comparable quantitative ratings across trials.
