# Supplementary material for: Effectiveness and Implementation of Digital Health Interventions on Physiological, Psychological, and Functional Outcomes in Adults With Multimorbidity: Systematic Review and Meta-Analysis of Randomized Controlled Trials
Source: J Med Internet Res. 2026 Jul 28;28:e90458. doi: 10.2196/90458 (PMC13412019; doi:10.2196/90458)
Supplement: Multimedia Appendix 10 [file jmir-v28-e90458-s010.docx]

**Table S1**. Coding framework used to classify digital health interventions

| **Study (author, year)** | **Open codes extracted from intervention description** | **Descriptive theme** | **Final category** |
| --- | --- | --- | --- |
| **Wakefield et al (2011) [41]** | daily BP/BG transmission, nurse case management, remote review | Daily monitoring with clinical feedback | **Telemonitoring** |
| **Liang et al (2021) [57]** | 24/7 tele-homecare, wireless devices, alerts, nurse follow-up | Integrated tele-homecare monitoring | **Telemonitoring** |
| **Tchalla et al (2025) [58]** | home sensors, biometric surveillance, telecare review | Home telemonitoring after discharge | **Telemonitoring** |
| **Bernocchi et al (2018) [56]** | remote monitoring, home exercise, weekly nurse/PT calls | Telerehabilitation with remote monitoring | **Telemonitoring** |
| **Mihevc et al (2025) [55]** | mHealth app, BP/BG upload, alerts, teleconsultation | mHealth telemonitoring | **Telemonitoring** |
| **Yoo et al (2009) [53]** | mobile phone plus internet, daily BP/BG/weight upload, automated SMS feedback | Integrated mobile-web monitoring | **Telemonitoring** |
| **Or et al (2020) [54]** | tablet platform, Bluetooth BP/BG devices, reminders, monitoring | Tablet-supported home monitoring | **Telemonitoring** |
| **Rifkin et al (2013) [45]** | Bluetooth BP cuff, home hub, wireless transmission, pharmacist/physician review | Wireless home BP telemonitoring | **Telemonitoring** |
| **Chan et al (2022) [36] (JADE)** | web portal, personalized reports, nurse calls, team review | Digital self-management platform with professional support | **Web/app-based self-management** |
| **Yao et al (2021) [31] (mAFA-II)** | mHealth app, ABC pathway, integrated follow-up, app-based care pathway | App-based integrated self-management | **Web/app-based self-management** |
| **Gustafson et al (2024) [62]** | web platform, symptom tracking, peer discussion, clinician reports | Web-based self-management and peer support | **Web/app-based self-management** |
| **Lear et al (2021) [64]** | internet platform, symptom reporting, nurse support, self-management tools | Internet-based self-management with monitoring | **Web/app-based self-management** |
| **Hwang et al (2025) [51]** | mHealth self-management program, app-based coaching, telephone support, group sessions | App-based self-management with coaching | **Web/app-based self-management** |
| **Landucci et al (2025) [43]** | ElderTree platform via laptop or smart display, self-management content, online meetups | Platform-based self-management support | **Web/app-based self-management** |
| **Chiang et al (2020) [61]** | telemedicine exercise program, app plus messaging support, home physiological feedback | Tele-exercise self-management | **Web/app-based self-management** |
| **Hsu et al (2021) [44]** | telemedicine dietitian follow-up, home exercise support, communication app | Tele-supported lifestyle self-management | **Web/app-based self-management** |
| **Ye et al (2024) [34]** | WeChat education, daily posts, Q&A, peer sharing | App-based education and self-management | **Web/app-based self-management** |
| **González-Ortega et al (2017) [65]** | biweekly telephone counseling, physician review, care coordination | Telephone-based coaching | **Telephone/collaborative care** |
| **Rollman et al (2021) [40]** | telephone collaborative care, nurse management, physician liaison | Telephone collaborative care | **Telephone/collaborative care** |
| **Gellis et al (2014) [59] (I-TEAM)** | telephone problem-solving treatment, nurse support, monitoring | Telephone-supported collaborative care | **Telephone/collaborative care** |
| **Stewart et al (2021) [60] (TIP)** | case conference, nurse coordination, multi-provider follow-up | Collaborative case-management approach | **Telephone/collaborative care** |
| **Panagioti et al (2018) [66]** | telephone health coaching, social prescribing, mood support | Telephone health coaching | **Telephone/collaborative care** |
| **Wang et al (2025) [33] (CIC-PDD)** | case manager, specialist-primary care coordination, integrated community follow-up | Collaborative integrated care | **Telephone/collaborative care** |
| **Baumeister et al (2021) [48]** | guided internet/mobile CBT, eCoach feedback | Guided digital psychological therapy | **Decision-support/psychosocial approaches** |
| **Monreal-Bartolomé et al (2025) [52]** | blended psychological program, face-to-face plus web modules, motivational support | Blended psychosocial intervention | **Decision-support/psychosocial approaches** |
| **Yu et al (2020) [37]** | web-based patient decision aid, goal setting before visits | Patient decision support | **Decision-support/psychosocial approaches** |
| **Jungo et al (2023) [38] (OPTICA)** | electronic CDSS, STOPP/START review, GP shared decision-making | Electronic prescribing decision support | **Decision-support/psychosocial approaches** |
| **Blum et al (2021) [39] (OPERAM)** | web-based medication review, STRIPA, pharmacist-physician optimization | Medication optimization decision support | **Decision-support/psychosocial approaches** |
| **Clarke et al (2019) [63]** | self-guided web CBT, symptom tracking | Unguided digital psychological intervention | **Decision-support/psychosocial approaches** |
| **Prabhakaran et al (2019) [32] (mWellcare)** | mHealth decision support, tablet-based CDSS, SMS reminders | Point-of-care digital decision support | **Decision-support/psychosocial approaches** |
| **Bothelius et al (2024) [47]** | internet CBT-I, therapist messaging | Digital insomnia-focused psychological therapy | **Decision-support/psychosocial approaches** |
| **Sanabria-Mazo et al (2023) [42]** | videoconference ACT/BATD groups | Digital group psychosocial therapy | **Decision-support/psychosocial approaches** |
| **Gasslander et al (2022) [46]** | tailored ICBT, clinician-guided modules | Guided digital psychological therapy | **Decision-support/psychosocial approaches** |
| **Araya et al (2021) [35] (CONEMO)** | smartphone behavioral activation app, nurse support | App-based psychosocial intervention | **Decision-support/psychosocial approaches** |
| **O’Moore et al (2018) [50]** | iCBT with email support | Guided digital psychological therapy | **Decision-support/psychosocial approaches** |
| **Schuffelen et al (2025) [49]** | digital CBT-I, automated modules, notifications | Automated digital psychological therapy | **Decision-support/psychosocial approaches** |

**Note:** Interventions were classified according to their primary function, core components, and dominant mechanism of action. For multicomponent interventions, classification was based on the element judged most central to the intended clinical effect rather than on all components delivered. This framework was developed inductively to support cross-study synthesis and should be interpreted as a descriptive analytic tool rather than a definitive taxonomy. Inter-rater reliability for this classification is reported in **Appendix Table S8**. In the final 36-study sample, several interventions combined digital, telephone-based, and face-to-face elements; these were classified according to the dominant clinical mechanism judged most relevant to the intervention’s intended effect.

**The four categories are defined as follows:**

- **Telemonitoring:** Interventions that use digital devices to remotely collect and transmit physiological or symptom-related data (eg, blood pressure, blood glucose, weight, or other monitored indicators) to health care providers, typically with automated alerts and/or professional review or follow-up. Core components include biometric sensors, data transmission platforms, and remote clinical monitoring.
- **Web/app-based self-management:** Interventions delivered primarily through websites, mobile apps, or patient-facing digital platforms that enable patients to actively manage their own health. Core components include educational content, self-monitoring tools, goal setting, reminders, behavior-change support, and in some cases gamification, peer support, or digital coaching.
- **Telephone/collaborative care:** Interventions delivered primarily through structured or semi-structured telephone contact and care coordination between patients and health care professionals. Core components include regular follow-up calls, collaborative care management, symptom review, medication support, and liaison with primary or specialty care.
- **Decision-support/psychosocial approaches:** Interventions that provide structured support for clinical decision-making or primarily target psychological, behavioral, or social dimensions of care. Core components include electronic clinical decision support systems, patient decision aids, medication review tools, and guided or structured digital psychological therapies (eg, internet-delivered CBT).

**Table S2:** Inter-rater reliability for intervention classification

| Phase | Studies coded | Agreement (%) | Cohen's κ (95% CI) |
| --- | --- | --- | --- |
| Pilot | 3 | 92.3 | 0.88 (0.71–1.00) |
| Full | 36 | 94.2 | 0.91 (0.82–1.00) |

**Notes:** Inter-rater reliability was assessed by two independent reviewers (RQ, HZ). Percentage agreement was calculated as the proportion of studies with concordant classifications. Cohen's κ was computed to adjust for chance agreement, using R (version 4.3.0). According to Landis and Koch [1977], κ values of 0.81–1.00 indicate "almost perfect" agreement.
