# Supplementary material for: Effectiveness and Implementation of Digital Health Interventions on Physiological, Psychological, and Functional Outcomes in Adults With Multimorbidity: Systematic Review and Meta-Analysis of Randomized Controlled Trials
Source: J Med Internet Res. 2026 Jul 28;28:e90458. doi: 10.2196/90458 (PMC13412019; doi:10.2196/90458)
Supplement: Multimedia Appendix 11 [file jmir-v28-e90458-s011.docx]

**Table S1:** Ongoing and planned digital health trials and implementation projects for multimorbidity.

| **Project / acronym** | **Registration / source** | **Country / setting** | **Target population** | **Target sample size** | **Digital health intervention** | **Timeline / latest public status** |
| --- | --- | --- | --- | --- | --- | --- |
| **Protocol- or registry-backed trials** | | | | | | |
| Shanghai proactive health behaviour intervention | ChiCTR2400088374; BMC Primary Care protocol (2026) | China (Shanghai; 14 PHCs) | Older adults with T2DM plus >=1 cardiometabolic comorbidity (hypertension, hyperlipidaemia, ischaemic heart disease) | Minimum 420 | Cluster-RCT using monitoring bracelets + mobile app, short-video education, and GP shared decision-making / goal support | Recruitment began Aug 2024; 24-month follow-up; final follow-up anticipated Jun 2027 |
| MultiLife | CTRI/2024/10/074559; BMC Public Health protocol (2025) | India (Odisha and Jharkhand; 18 PHCs) | Adults with cardiometabolic multimorbidity (diabetes, hypertension, and/or cardiovascular disease risk/history) | 840 | mHealth lifestyle toolkit with daily digital reminders, weekly education broadcasts, and primary-care support | Registered Oct 2024; implementation planned for 2025-2026 |
| VCoP / e-EMPODERAT | NCT06046326; BMJ Open protocol (2024) | Spain (Madrid and Canary Islands) | Adults aged 30-60 years with >=2 chronic conditions | 240 | Two web-based self-management education offers comparing individual online education versus virtual community of practice | Protocol published 2024; 18-month pragmatic RCT |
| ProACT / SEURO | ISRCTN34134007; JMIR Research Protocols protocol (2025) | Ireland, Belgium, Sweden | Adults aged >=65 years with >=2 of diabetes, chronic respiratory disease, chronic heart failure, chronic heart disease | 720 participants; up to 1500 care-network members | Three-arm trial of updated ProACT platform with / without clinical triage and care-network support | Enrollment began Sep 2022; anticipated end Mar 2026; outcomes expected in 2026 |
| METHIS | NCT05593835; BMJ Open protocol (2023) | Portugal (Lisbon and Tagus Valley primary care) | Adults aged >=50 years with complex multimorbidity (>=3 chronic conditions affecting >=3 body systems) | 1380 across ~60 practices | Goal-oriented care supported by the Multimorbidity Management Health Information System (METHIS) plus clinician training | Protocol published 2023; registry listed; trial planned from Jun 2025 |
| VetASSIST | NCT05560451; Contemp Clin Trials protocol (2025) | United States (Veterans Health Administration) | Veterans with >=3 chronic conditions across >=3 body systems | 294 | Video-based peer health coaching (20 visits over 12 months) for self-management, goal-setting, and social support | Protocol published 2025; recruitment / follow-up ongoing |
| gp-multitool.de | NCT06061172; BMJ Open protocol (2025) | Germany (general practice) | Adults aged >=65 years enrolled in a disease management programme plus multimorbidity operationalised by two additional chronic conditions | 660 from 66 GP practices | Web application implementing the German S3 multimorbidity guideline, including digital assessments, medication review, and GP discussion prompts | Protocol published 2025; registry listed for cluster-randomised trial |
| Mobili | NCT07175545; registry + protocol PDF (2025) | Portugal (community pharmacy) | Community-dwelling patients with chronic multimorbidity / polypharmacy | Expected 96 (pilot) | Smart medication dispenser embedded in pharmacist-led medication management service | Not yet recruiting in latest public registry view |
| TCM Constitution Database & AI-HEALS system | ChiCTR2500113269; Chinese registry (2025) | China (Zhejiang) | Patients with comorbid hypertension, hyperglycaemia, and/or hyperlipidaemia | 195 | Registry describes an AI recommendation system integrating a TCM constitution database with multimodal large language models | Registry-only record; not yet recruiting |
| **Funded implementation projects with explicit digital-health components** | | | | | | |
| MULTIPULM | Horizon Europe 101226783; CORDIS + project website | Brazil, Serbia, Turkiye | People with chronic respiratory conditions and multimorbidity | Approx. 2750 in clinical validation | Integrated digital care ecosystem combining clinical tools, patient-engagement platforms, monitoring tools, and implementation pathways | 48-month project (2025-2029); no main outcomes yet |
| EMPOWER | Horizon Europe 101226675; CORDIS | Benin and Togo | People living with multiple long-term NCDs, especially cardiometabolic disease, epilepsy, and common mental disorders | Not publicly reported | CHW and primary-care training plus Android app for screening, monitoring, and provider communication using WHO-PEN / mhGAP | Start Sep 2025; end Aug 2029 |
| HIVE | Horizon Europe 101226666; CORDIS | Kenya, Kazakhstan, Greece, Malta | People living with HIV and multiple long-term conditions | Not publicly reported | mHealth self-management app for PLWH plus psychosocial counselling and NCD-care integration into HIV clinics | Start Jul 2025; end Jun 2028 |
| GACD MC02 digital mobility solution | GACD MC02 project page | Brazil | Older adults with multiple chronic conditions | Not publicly reported | Co-designed digital solution to optimise mobility | Project listed for 2025; detailed protocol not yet public |

**Abbreviations:** GP, general practitioner; PHC, primary health centre; PLWH, people living with HIV; RCT, randomised controlled trial; T2DM, type 2 diabetes mellitus.

**Note:** This table provides a supplementary overview of ongoing or planned randomised controlled trials and large-scale implementation projects evaluating digital health interventions for adults with multimorbidity. Records were identified through supplementary searches of public trial registries, protocol repositories, and major research programme databases conducted in March 2026, after completion of the primary systematic search on June 7, 2025. Studies were included if they explicitly targeted multimorbidity, evaluated a digital health intervention, and had no published results at the time of searching. LMIC trials were defined as studies with at least one implementation site located in a low- or middle-income economy according to the World Bank classification. Given the exploratory nature of this mapping and the dependence on publicly accessible records, this table should be interpreted as indicative rather than exhaustive. Study characteristics, status, and timelines may be subject to change and should be verified against original sources.
